# A Required Ophthalmology Rotation: Providing Medical Students with a Foundation in Eye-Related Diagnoses and Management

**DOI:** 10.15766/mep_2374-8265.11100

**Published:** 2021-02-12

**Authors:** Eve M.R. Bowers, Brittany Perzia, Rikki Enzor, Owen Clinger, Sanya Yadav, Patrick W. Commiskey, Peter Mortensen, Evan Waxman

**Affiliations:** 1 Third-Year Medical Student, University of Pittsburgh School of Medicine; 2 Third-Year Medical Student, Stony Brook School of Medicine; 3 PGY-4 Ophthalmology Resident, Department of Ophthalmology, University of Pittsburgh Medical Center; 4 First-Year Medical Student, University of Pittsburgh School of Medicine; 5 Second-Year Medical Student, University of Pittsburgh School of Medicine; 6 PGY-2 Ophthalmology Resident, Department of Ophthalmology, University of Pittsburgh Medical Center; 7 Residency Program Director and Associate Professor, Department of Ophthalmology, University of Pittsburgh Medical Center

**Keywords:** Ophthalmology, Clerkships, Case-Based Learning, Clinical Skills, Clinical Teaching/Bedside Teaching, Editor's Choice

## Abstract

**Introduction:**

Current ophthalmologic training in medical school is inadequate in preparing medical students to handle basic eye complaints as nonophthalmology residents. Most medical students are uncomfortable performing eye examinations, but increased ophthalmology training improves confidence in this area. The University of Pittsburgh School of Medicine (UPSOM) teaches students the basics of ophthalmology with a required 1-week rotation during the 1-month specialty care clerkship (SCC), providing students with skills to perform rudimentary eye examinations as nonophthalmology providers.

**Methods:**

Within a 1-week ophthalmology rotation, we developed a series of interactive case-based teaching sessions, handouts, and homework that accompanied clinical instruction to familiarize third- and fourth-year medical students with ophthalmic equipment, terminology, diagnosis, and management. Of learners, 67 (roughly 11 per cohort) rotated on six consecutive SCCs beginning in May 2019. All learners completed an in-house exam and received resident clinical evaluations at the end of their rotation.

**Results:**

Of the 64 participants who responded to the survey, 100% rated the quality of teaching sessions *outstanding* or *good*, and 83% of students strongly agreed or agreed with the statement, “I believe the overall teaching in the ophthalmology clinical settings was good quality.” The average clinical and exam score for ophthalmology over 6 months was 4.5 out of 5, and 83% respectively.

**Discussion:**

Generally positive student feedback as well as high clinical and exam scores suggested that the required UPSOM ophthalmology clerkship was both engaging and effective. This course can be easily adapted to teach students at other medical institutions.

## Educational Objectives

By the end of this curriculum, learners will be able to:

1. List common ocular conditions and describe their treatment.
2. Complete basic aspects of the comprehensive ophthalmologic examination including vision and pupil testing, slit lamp examination, and direct ophthalmoscopy.
3. Take a comprehensive ophthalmologic history and present patients using the appropriate format.
4. Describe ophthalmologic emergencies and when to refer to an ophthalmologist.
5. Experience ophthalmic surgery through time spent in the operating room.

## Introduction

Preparing medical students to provide ophthalmologic care is a challenge in medical education. There has been a decades-long documented decrease in instruction.^1^ In 2014, Shah et al.^2^ reported that only 18% of medical schools required a clinical ophthalmology rotation, a decrease from 68% in 2000.^2,3^ An increasingly crowded curricula, heightened competition among medical specialties for teaching time, decreased advocacy by ophthalmology educators, and curriculum committees unconvinced of a required ophthalmology rotation's utility were considered factors in the decline.^4,5^ As a result, medical school graduates are not prepared to address their patients' ophthalmologic complaints.

Stern reported a widespread concern among nonophthalmology residency program directors that residents did not meet the ophthalmology competency standards outlined by the Association of University Professors of Ophthalmology (AUPO).^6^ These standards were most recently revised in 2007 and state that the general physician should be able to recognize, triage, and use ocular signs to complement the diagnosis and management of systemic disease.^7^ The International Council of Ophthalmology (ICO) also recommends a competency-based approach to medical education that emphasizes the medical knowledge of eye diseases, anatomy, and ocular manifestations of systemic diseases, as well as clinical skills needed to test eye visual acuity, visual fields, extraocular movement, and ancillary signs of eye health.^8^ Although these guidelines are in place, many medical students still report discomfort in performing clinical techniques such as direct ophthalmoscopy.^9,10^ A lack of exposure to ophthalmology may result in students discounting ophthalmology as a career. In addition, physicians that graduate from medical school without ophthalmology experience are unlikely to understand the importance of eye health and the scope of practice of ophthalmology.

While there is a clear need for formal ophthalmology training in medical schools, few published educational tools exist to meet this demand. To our knowledge, there are currently no publications in *MedEdPORTAL* detailing ophthalmic curricular content for medical students in their clinical years. Available evidenced-based ophthalmology curricula for preclinical and clinical medical students included use of integrated teaching,^11^ team-based learning modules,^12^ and intensive 1- and 2-day training courses.^13–15^ While we did not find curricular content similar to our own on *MedEdPORTAL*, our literature search yielded one related article. In 1976, Waning et al. presented in the Journal of Medical Education a highly successful 10-day ophthalmology rotation based on pre- and posttest data, which demonstrated the feasibility of an ophthalmology clerkship for clinically rotating students.^16^

To address the paucity of ophthalmic educational content, we presented the ophthalmology curriculum at the University of Pittsburgh School of Medicine (UPSOM) as a model to meet the current challenges and goals of ophthalmology education. The comprehensive ophthalmology curriculum included interactive teaching sessions and provided students with the clinical skills and knowledge necessary to recognize ophthalmologic emergencies and perform eye examinations as nonophthalmologists in accordance with AUPO and ICO recommendations. While the teaching materials have been in use in our required ophthalmology clerkship for over 20 years, it occurred to the authors only recently that other programs might benefit from their use. Our primary goal in presenting this work was to share the teaching materials in use in our required ophthalmology clerkship with the hope that they may be beneficial to others.

## Methods

### Curriculum Development and Setting

One University of Pittsburgh Medical Center (UPMC) ophthalmology faculty member developed the original ophthalmology rotation curriculum in 1999. Minor course updates have been made since then, including the revision of course material to reflect changes in current practice and prescribing patterns. Some of these revisions, especially around the treatments and technology related to macular degeneration, have altered group discussion over the years. However, the current 2019 syllabus and overall structure of the 1-week clerkship and educational materials are largely the same today. The materials included have been modified to ensure that they are appropriate for wider use. The substitution of photos and creation of an instructor's guide (Appendix A) and answer keys served to improve the materials.

The 1-week ophthalmology rotation was part of the larger 1-month SCC that also covered 1 week of otolaryngology and 2 weeks of emergency medicine. Course content accommodated clinically rotating third- and fourth-year medical students with a wide range of previous ophthalmologic knowledge and was created with the education of a future nonophthalmologist in mind.

### Implementation

The required UPSOM ophthalmology clerkship consisted of interactive case-based teaching sessions, handouts, and homework as well as full-time clinical duties in outpatient settings. We gave students a course syllabus containing a clerkship overview, duty expectations, and the course director's contact information, as well as an ophthalmology-made-easy handout (Appendix B) with basic ophthalmology-specific advice and terminology. We emphasized AUPO-recommended clinical competency standards through four, 1-hour, in-person teaching sessions where attendance was highly encouraged.

The first teaching session included a case-based overview of minimum physician competency standards outlined by the AUPO and also covered basic science and societal implications of disease and management (Appendix C). This overview highlighted the classic presentations and causes of red eyes, traumatized eyes, abnormal eye movements, and abnormal pupils, while reviewing when to refer to an ophthalmologist. The remaining case-based teaching sessions relied on class participation to explore common ophthalmic pathologies including macular degeneration, cataracts, glaucoma, acute vision loss, diabetic eye disease, and amblyopia and strabismus (Appendix D). These cases walked through common eye conditions and contained questions meant to generate thoughtful differentials and stimulate class discussion about diagnosis and management. The acute vision loss teaching session required students to complete a chart with questions corresponding to nine photos of the retina prior to attending this unique session (Appendix F). Students brought this completed chart to class and participated in a preceptor-led conversation about non-red eye, acute vision loss emergencies, emphasizing the urgency, treatment, and common outcomes of each condition. These teaching sessions generated group discussion, reviewed common eye pathology, highlighted ophthalmic emergencies, and taught students when to refer to an ophthalmologist. Students had access to an online teaching session answer key (Appendix E), and they received a handout that supplemented each teaching session (Appendix F). This handout provided information about epidemiology, etiology, signs, symptoms, and treatment of conditions discussed in the cases and reviewed important definitions. Students were also given a primary text, *Basic Ophthalmology: Essentials for Medical Students*,^17^ which they were encouraged to read before each teaching session. Teaching session presentations, answer keys, handouts, and other course resources could be accessed at any time by students on UPSOM's online learning management system.

In clinic, students worked one-on-one with ophthalmology residents during the 40-hour work week where students learned the basics of ophthalmic equipment, terminology, and differential diagnoses. Students practiced basic eye examination skills such as vision and pupil assessment, extraocular movements, confrontation visual fields, and intraocular pressure. They took complete histories, performed parts of the physical exam, administered eye drops, and presented to attendings. Residents answered questions, received and provided feedback, and taught students about common ocular conditions and management throughout the week. Students recorded their patient encounters through an online learning log.

Students also spent a half-day observing in the OR with attending ophthalmologists, took call with residents, and attended ophthalmology grand rounds as mandatory clerkship activities.

### Curriculum Assessment

We used quantitative examination and clinical grading methods to assess students' comprehension following course completion. The written in-house examination consisted of 10 questions created by the ophthalmology course director which were based on the suggested reading and session materials (Appendix G). Most questions contained two to three subquestions, were short answer, and were associated with photographs and illustrations. The test was graded out of 10, but students could earn partial credit on each subquestion. The ophthalmology questions contributed to one-third of the total SCC final exam score, with otolaryngology and emergency medicine questions comprising the remaining two-thirds. The otolaryngology and emergency medicine examination portions were multiple choice and were created by both rotations' respective course directors. Residents graded students with whom they directly worked on a 5-point scale in four categories: clinical knowledge, clinical skills and data reporting, clinical reasoning and problem solving, and professionalism. The students' clinical ophthalmology grades were averaged and reported out of 5 points. This clinical grade contributed to one-fourth of their overall SCC clinical score, and these quantitative data were valuable for assessing the effectiveness of the curriculum.

The UPSOM Office of Medical Education administered a postrotation survey (Appendix H) consisting of five questions gauging the quality, strengths, and weaknesses of the course. The first question used a 5-point Likert scale to evaluate the quality of teaching sessions (5 = *outstanding*, 4 = *good*, 3 = *satisfactory*, 2 = *fair*, 1 = *poor*), while the second question used a 5-point Likert scale to evaluate students' agreement with the statement, “I believe the overall teaching in the ophthalmology clinical settings was good quality” (5 = *strongly agree*, 4 = *agree*, 3 = *neutral*, 2 = *disagree*, 1 = *strongly disagree*). The remaining three questions consisted of open-ended feedback prompts. Finally, students completed a minimum requirement of 100 learning logs for the entire SCC clerkship, without specialty minimums. Students logged meaningful patient interactions that provided information about category and quantity of patient diagnoses seen.

## Results

Sixty -seven UPSOM third- and fourth-year medical students on clinical clerkships participated in the required ophthalmology rotation at one of three UPMC clinical sites over 6 months during the first half of the 2019–2020 academic year (May – October 2019).

Postrotation survey responses revealed that the 64 students who responded to the survey found the ophthalmology teaching sessions to be of *outstanding* or *good* quality. Of students, 83% strongly agreed or agreed that the clinical teaching was good quality, and no students strongly disagreed.

In response to the three open-ended questions, students felt strongly that the case-based teaching sessions were particularly beneficial to their learning and identified resident teaching, physical exam practice, team community, the operating room, and course organization as positive aspects of the course. Of students who responded, 19% (12 of 64) believed that the ophthalmology course involved too much time spent shadowing. Three students requested online lectures and modules.

We analyzed the ophthalmology clinical grades and ophthalmology exam scores of each cohort consisting of eight to 13 third- and fourth-year medical students. Students' individual clinical scores ranged from 3.0 (*n* = 3) to 5.0 (*n* = 18) out of 5, and the average individual clinical score was 4.5 (*SD* = 0.5). The average clinical score by clerkship cohort ranged from 4.2 (*SD* = 0.8) to 4.8 (*SD* = 0.3). Medical students' ophthalmology exam scores ranged from 39% correct (*n* = 1) to 100% correct (*n* = 3). Over two-thirds (70%) of students scored at least 80% on the exam, and almost two-fifths (39%) of students scored at least a 90%. The average ophthalmology exam score by clerkship cohort ranged from 77.1 (*SD* = 12.3) to 89.1 (*SD* = 9.0), and the average individual ophthalmology score was 83 (*SD* = 13.8).

## Discussion

The required ophthalmology rotation at UPSOM fills a gap in medical school training. Most medical students lack the confidence to perform eye examinations,^9,10^ yet competency in the basics of ophthalmology is essential to safely evaluate patients with eye complaints in primary care, emergency room, neurology, and other nonophthalmologic settings.^18,19^ Above-average clinical grades, high exam scores, and completed learning logs show that the UPSOM clerkship effectively familiarized students with ophthalmic equipment, diagnosis, and management. Other institutions may experience similar success by adopting the modular and disseminatable components of our course; most notably, the teaching sessions and student handouts. Through interactive, case-based sessions, our students learned how to evaluate red eyes, traumatized eyes, abnormal pupillary responses, and abnormal eye movements, all of which are AUPO standard competencies for medical school graduates.^6^ Herein, we provided the material needed to replicate these educational experiences to offer effective, ready-to-use content for ophthalmology educators at other institutions.

Based on objective and open-ended student feedback, the teaching sessions and handouts were the most well received facet of our course. Students specifically commented that the ophthalmology-made-easy handout and case-based teaching sessions were positive aspects of the course, and noted these materials to be superior to the ear, nose, and throat and emergency medicine presentations. Faculty presented ophthalmology teaching sessions in a case-based format, as this teaching style is evidenced to enhance knowledge,^20,21^ cultivate teamwork,^22^ and improve patient care.^23^ Data also suggests that case-based learning is more effective than conventional lecture-based methods at teaching students content,^24^ and we believe that this format contributed to students' overwhelmingly positive reception and high examination scores.

In the clinical setting, most students agreed that the teaching was high quality, and multiple students remarked that residents taught at an appropriate level and provided active involvement. Overall, student attitudes toward the clinical portion of the ophthalmology rotation varied, and clinical experiences were somewhat dependent on resident instructors. While most students mentioned that their assigned resident gave them interactive, autonomous, and hands-on experiences, some students complained about a shadowing-only experience. Parenthetically, clinical feedback was used by the residency program to inform their professionalism and practice-based learning competencies. Assigned clinic site and grand rounds did not seem to affect student experience, and interactions with attendings had only neutral or positive impacts.

We collected 6 months of data from a course that has been developed, implemented, and improved upon for almost 20 years. While the relatively brief study period in which we collected data on medical student experiences and performance may offer an incomplete assessment of our curriculum, our primary goal in presenting this work was to share the materials in use for our required ophthalmology clerkship with the hope that they may be useful to others, rather than to evaluate student satisfaction and performance over time. We provided student satisfaction data knowing that the likely consumer of the materials—an instructor interested in adapting them to the unique needs of their own medical school—would likely be interested in knowing that the materials were well received by our most recent students. The short duration of the course and flexibility afforded by teaching sessions and other ancillary activities permits adaptation of this model to other schools. The teaching sessions and session handouts can be readily implemented at other institutions, as only a small lecture space, facilitator, and electronic or printed handouts are required.

Medical schools with smaller ophthalmology departments lacking the faculty to deliver in-person teaching sessions might consider using real time, online lectures or even recorded talks supplemented with online modules. Notably, feedback from students welcomed online teaching materials, but this poses limitations, especially to students at programs without ophthalmology departments, which would likely rely on recorded lectures. Case-based teaching sessions are dependent on student participation and class discussion to facilitate learning, making recorded lectures less effective. This drawback may be overcome by the recruitment of community ophthalmologists or faculty at other institutions to facilitate live online Zoom or in-person sessions for students at medical schools with limited faculty. If this is not feasible, teaching sessions could also be led by primary care physicians, though ophthalmologist facilitators are preferable since ophthalmologists are more effective at teaching students ophthalmic content than nonophthalmologist providers.^14^ As a last resort, teaching sessions could be formatted into traditional lectures and recorded.

Students' clinic duties, night call, and half-day OR shadowing were highly successful components of the course, evidenced by high clinical scores and positive feedback. UPMC's busy outpatient clinics and 18-person resident physician team afforded students ample patient exposure and one-on-one teaching time, which undoubtedly contributed to students' clinical accomplishments. Replicating this clinical experience in schools with smaller ophthalmology departments may be more challenging. With fewer residents and attendings, schools may find it difficult to assign a single teacher to every medical student, compromising individualized clinical instruction. If clinic volume is lower, this may result in decreased opportunity for medical students to engage in history taking, exam practice, and presenting. While our program required a sizable commitment of resources that not all institutions have available, we recognized it as having several strengths over alternative approaches such as computer-based instruction and 1-day curricula where working with patients and skill reinforcement may be unachievable.^14,25^ However, we acknowledge that alternative methods have merit in situations where a lack of resources does not permit our approach. Medical schools with a smaller ophthalmology faculty or without an ophthalmology department may consider creating partnerships with local ophthalmologists in private practices or unaffiliated hospitals to ameliorate the challenge of limited faculty.

Regardless of program size, a required ophthalmology rotation could also be impeded by institution-dependent medical school curricula and tight time constraints of third-year clinical rotations. Nonetheless, the general flexibility of fourth-year schedules may provide freedom for medical schools to offer this course as a required or at least an elective course. In a 2015 survey of 1,367 fourth-year US medical students, 34% of students applying to primary care specialties and 20% of those applying to nonprimary care specialties reported that the fourth year's main purpose is to gain broad educational experiences,^26^ which would prepare them well for residency training and future independent practice. This rotation provided a worthwhile means of broadening students' educational experiences with the end goal of preventing future residents from missing sight- or life-threatening pathology.

A final limitation worth noting was that this study assessed short-term retention of highly technical and specialized skills and material. Future studies may consider evaluating how effectively a required ophthalmology rotation of this duration propagates long-term ophthalmologic capabilities. It may also be useful to administer similar surveys and exams at other institutions with required ophthalmology rotations in order to achieve more generalizable results.

The AUPO specifies which ophthalmologic competencies all general physicians should fulfill in its policy statement on medical student education.^6^ Clinical evaluations and exam scores demonstrated that students mastered eye conditions far beyond AUPO stipulations, and learning logs showed they observed a wide range of diagnoses. Our approach complemented preclinical and elective training, which prevents the attrition of clinical skills that has been reported in the literature.^27,28^ For these reasons, we believe the UPSOM-required ophthalmology clerkship surpassed the minimum competency standards outlined by the AUPO and ICO and effectively positioned students to address patients' basic ophthalmic complaints as nonophthalmology providers. We presented our course design to provide a framework that other institutions may adapt in order to offer a similar learning experience through a required or elective rotation. We hope that such courses will reverse the current decline in required ophthalmology instruction, address the insufficiencies in ophthalmologic training in medical schools, and increase student confidence and competency in this area.

## Appendices

Ophthalmology Slides Instructors Guide.docxOphthalmology Handout.docxOphthalmology Slides.pptxOphthalmology Sessions.docxOphthalmology Sessions Answer Key.docxOphthalmology Sessions Student Handouts.docxOphthalmology Final Examination.docxStudent Postrotation Feedback Form.docx
All appendices are peer reviewed as integral parts of the Original Publication.
