# Supplementary material for: A Required Ophthalmology Rotation: Providing Medical Students with a Foundation in Eye-Related Diagnoses and Management
Source: MedEdPORTAL. 2021 Feb 12;17:11100. doi: 10.15766/mep_2374-8265.11100 (PMC7880261; doi:10.15766/mep_2374-8265.11100)
Supplement: Supplementary file 1 — Ophthalmology Slides Instructors Guide.docxOphthalmology Handout.docxOphthalmology Slides.pptxOphthalmology Sessions.docxOphthalmology Sessions Answer Key.docxOphthalmology Sessions Student Handouts.docxOphthalmology Final Examination.docxStudent Postrotation Feedback Form.docx [file mep_2374-8265.11100-s001.zip › A. Ophthalmology Slides Instructors Guide.docx]

This manual is the script for the Ophthalmology Made Easy PowerPoint. It serves to guide whoever is presenting this lecture.

Slide Number:

1. Introduction
2. The objectives of this presentation include: Identifying the Association of University Professors of Ophthalmology (AUPO) Goals, reviewing cases and management of red and traumatized eyes, reviewing causes and treatment of abnormal eye movements and pupils, and recognizing and describe when to refer to an ophthalmologist.
3. The Association of University Professors in Ophthalmology has taken it upon itself to list set of goals it believes all physicians should be able to perform. Essentially, these are the things they feel you need to know to know when and how to treat, when and how urgently to refer.
4. In case anyone is feeling particularly insecure that you’re the only medical school students to feel a little shaky on this stuff you can take some comfort in the following statistics taken from a survey of residency program directors by George Stern in 1995. Only 33% of program directors of internal medicine residency programs believe their graduates meet these goals. 90% believe that fewer than 50% of incoming residents meet these standards. There’s little similar data regarding Emergency Medicine (EM) residencies in the U.S, but Tan et al in 1997 in a study of UK EM house officers found that 26% had no training in eye emergencies, 68% had little or no confidence in dealing with the eye, and 42% worked in an Emergency Department without a slitlamp. When you consider that most medical students get a total of 5-10 working days (or less) of clinical ophthalmology training during their third and fourth year this is not actually that surprising.
5. With that introduction, let’s run through these goals and try to make you more comfortable with each of them. For the most part, this presentation will consist slide caselets, a little history and a photo. As medical students, your skill in taking a focused history gives you a big advantage in nailing a diagnosis quickly and in distinguishing an emergency from an urgency from a routine trouble. Along the way, we will go over do’s and don’ts and clear up some areas of confusion shared by patients and non-ophthalmologic physicians. So, let’s look at goal 1 -- measure & record visual acuity.
6. The first two cases definitely stretch the definition of the word emergency, but this happens. A 24-year-old man who recently moved from Manhattan, went to the Department of Motor Vehicles (DMV) to get his first driver’s license and failed the eye test. He has no significant past medical or eye history. He thinks his eyesight is great and proceeds to demonstrate this by reading the 20/20 line on your pocket eye chart. The DMV form he’s waving around says he has 20/50 vision in either eye. This one’s a little too easy, and I’m sure you recognized quickly that this fellow is nearsighted. But the case raises some important points. First, it shows you one of two easy ways to distinguish eye disease from a refractive problem, i.e., the patient needs glasses. If a person’s vision measures differently at different distances, then you can be sure that the patient needs glasses. The other way to quickly screen if a patient’s vision problem is just refractive is to have them look through a pinhole. If the vision improves, then they’ll improve with glasses. The optical principles involved are beyond the scope of this talk, but it also explains why people who turn out to be nearsighted often walk into a doctor’s office complaining that they’re blind at night. In the dark, the pupil dilates largely giving them the opposite of a pinhole effect.
7. The other question the case raises is, “What is the guy’s visual acuity?” Is it 20/50, or 20/20? The answer is both. Everyone, at some point, gets taught a definition like this one for acuity. The top number refers to distance measured, and the bottom number refers to a distance that a “normal” eye could read the same figures. But no one thinks about it this way, and it doesn’t explain how one can be 20/20 at near.
8. Don’t think about it that way. Instead, think: 20/50 at far means that the person can read the 20/50 line at 20 ft and 20/50 at near means the person can read the 20/50 line at 14 inches with a near card. The point is, one must note how the vision was taken in order to properly record and communicate the patient’s vision.
9. Before going on to the only other refraction case, it’s useful to go over some milestones in the vision (va) chart. 20/20 is normal vision, 20/15 is supernormal, and most young, healthy people can be corrected to 20/15. 20/40 is the DMV’s minimum for driving. Patients need to see at least this well from their better eye to pass the driving test. 20/200 is the big E on most hanging eye charts. Patients with 20/200 but won’t stumble into chairs. 20/200 and even 20/400 is still excellent walking around vision. Once the patient can’t read the eye chart, patients’ vision is recorded as “CF” or “count fingers” at a particular distance. The next step down is “hand motion” detection, then light perception only, and finally NLP, or no light perception. (Legal blindness is defined by the federal government, based on the vision in the better seeing eye. To be legally blind, the better eye must have either distance visual acuity of 20/200 or worse, or a visual field measuring 20 degrees or less. Both eyes must have poor vision for a patient to qualify as legally blind. The driving requirement varies by state).
10. This is another refractive case. A 42-year-old (yo) woman pilot complains that she can’t read the forms she needs to fill out her flight logs. You check her at the acuity chart and her visual acuity is 20/15 at far. When you check her with a near card, she only gets down to the 20/50 line. So, we know that this woman’s problem is refractive. Her vision is different at different distances. If nearsighted people have trouble seeing faraway, then farsighted or hyperopic person must have trouble seeing close-up, so this woman must be farsighted. Wrong! Far-sighted people actually don’t usually complain of blurry vision at near distances. Most people under the age of 40 have enough accommodation (near focusing power) to overcome any farsightedness they have. Instead, farsighted people present with a complaint of headache or tired eyes when reading for any extended period of time. You can think of this as muscle fatigue of muscles of accommodation. This woman has passed the magic age of 40, at which people’s lenses begin to stiffen, and they lose their ability to accommodate. This is called presbyopia, and we can save this patient a lot of time and money by directing them to try out the over the counter reading glasses at the local pharmacy or grocery store.
11. To sum up the first section, here are the important points for measuring and recording visual acuity. 1. Always record how the vision was taken. Near or far, with or without glasses. 2. Always use the patients’ correction if they have it (meaning have them wear their glasses or contact lenses while they read the eye chart). Blurry vision without my glasses on is a frequent patient complaint, but it is not eye disease. 3. Always measure each eye separately. 4.If a patient complains of decreased vision, detect refractive problems by either checking at different distances or using a pinhole.
12. Next, the red eye.
13. This is a pretty nasty looking red eye and is supposed to be an example of acute bacterial conjunctivitis. Conjunctivitis usually isn’t this ugly, so we will concentrate on some of the more routine red eyes.
14. This is a 28 yo man with a history of red, itchy eyes for the past few days. Both eyes are involved. He has no past medical history (PMH) except for some seasonal allergies. His vision is 20/20 at near in both eyes. When you flip up his upper eyelid, there is a red and bumpy conjunctiva. This is known as a papillary reaction and it’s a hallmark of allergic conjunctivitis, but this is not necessary to make the diagnosis. Conjunctivitis should be thought of like rhinitis and pharyngitis. The tissues are similar, the location is similar, and, not surprisingly, the processes are similar. In this case, the patient’s allergic history, the bilaterality, and the chief complaint of itching should make you immediately think of allergy just as it would if the patient came in with a runny stuffed up nose instead of a red eye. The majority of patients will do well with cool compresses and even a systemic antihistamine. Topical vasoconstrictors like naphazoline-pheniramine (Naphcon) work but just like with oxymetazoline (Afrin) for rhinitis, patients will often notice a bounce-back phenomenon when they try to come off them. For cases you feel you need to treat, there are three ways to go. Over the past few years, a number of antihistamine and mast cell degranulation inhibitors have become available. Combination drops utilizing both mechanisms are safe, effective and have both quick onset of action and long-lasting effects for chronic atopies. The names of these medications, in no particular order, are olapatadine (Patanol), nedochromil (Alocril), ketotifen (Zaditor), and azelastine (Optivar). If a patient’s allergic symptoms are bad enough to consider topical steroids, they’re generally bad enough that you want an eye consult. This is a case of allergic conjunctivitis.
15. This is a 32 yo woman whose troubles began with a mildly irritated red left eye 5 days ago. Two days later, it spread to the right eye as well. Her eyes feel sandy, gritty, and sore. She says her vision is sometimes blurry but clears when she blinks. Her 5-year-old son had the same thing recently. Once again, think about this the way you’d think about a sore throat. You’ve got a red eye that’s spread from son to mother and now from eye to eye. On exam, both eyes are injected, which means that the surface blood vessels are dilated. Also, the patient has a little tender swelling in the preauricular area. Infectious etiology should be at the top of the differential here. The organisms to think about are pretty much the same as for pharyngitis. Adenovirus, Staph Aureus, Pneumococcus, and H Flu. Just as in pharyngitis, note the discharge and decide whether you’ve got a bacterial infection or a viral infection, with purulent discharges pushing you toward bacterial. Just as in pharyngitis, you often can’t tell and it’s often a matter of style as to whether to send the patient home on supportive therapy such as cool compresses and artificial tears or to treat them with antibiotics. It’s useful to tell patients with suspected viral conjunctivitis that it often follows a 2-week course and may get a little worse before it gets better. A reminder to the patient that increased hygiene (i.e., handwashing and separate towels) may stop the spread to the rest of the family. School-age children and healthcare workers should be advised to stay home until the eye is no longer red. This is a case of viral conjunctivitis.
16. A week later, the 32 yo woman returns. Being of the “treat it if you can’t tell” persuasion, you had asked her to use gentamicin eye drops, and initially she felt somewhat better. After a few days, she began to notice that her eye was getting redder, and the drops burned when she put them in her eye. On exam, you see marked conjunctival swelling known as chemosis. Exam of the conjunctiva in the lower fornix shows a red lumpy appearance. This is drug toxicity. The physical findings are typical, but you should be able to make the diagnosis based on history. In the absence of purulent discharge, you can be pretty sure this is not an infection resistant to the gentamicin. Instead, its the medication itself causing trouble. For some reason, gentamicin seems particularly prone to do this. gentamicin is a good drug, and it covers all the bacteria we talked about. But erythromycin or bacitracin ointment have pretty good coverage without the toxicity and with the benefit of lubricating the eye. If your patient won't tolerate an ointment because it blurs vision for a few minutes, sulfacetamide drops or polymyxin/trimethoprim drops have excellent coverage and surprisingly fewer toxic reactions than gentamicin. The treatment for this woman now is like an allergic or viral conjunctivitis. Cool compresses, artificial tears and time. This is medication-induced allergic conjunctivitis.
17. This is a 22 yo college student who woke up this morning and became frightened when he saw his eye in the mirror. He has no pain and no change in vision. He is quite anxious. On review of systems, you discover he threw up once after drinking last night, but he is otherwise well. This is a subconjunctival hemorrhage. Notice that rather than swollen conjunctiva with dilated blood vessels there’s a well outlined red area that’s really just some blood trapped under the conjunctiva. Patients have no pain or loss of vision if this is the only problem, but they’re usually frightened that they have something horrible. Most of the time etiology, when it’s not trauma, is unclear. Uncontrolled hypertension or diabetes can do this. Occasionally, you’ll find that the patient is Valsalva-ing from constipation or coughing a lot. Air flights can occasionally do this. Treatment is strictly reassurance that the redness will clear in two or three weeks. Sub-conjunctiva hemorrhage that doesn't clear or that returns over and over is an indication for a coagulation workup and an eye consult.
18. This time, the patient is a colleague. A 25 yo resident woke up post call with an angry eye. It’s red, it stings, and her vision is blurry. She’s an extended-wear contact lens user and frequently sleeps with her lenses in. On exam, you see a conjunctivitis, but with a lot of discharge and with a fuzzy white spot on the cornea. This is a corneal ulcer, an infiltrate in the stroma of the cornea with an overlying corneal epithelial defect. The history tips you off immediately that this is not just a conjunctivitis. It hurts too much, and the vision is reduced. Contact lens wear is a big risk factor for corneal ulcer. Extended/overnight wear raises that risk 10-to 20-fold. Extended, overnight contact lenses are a myth. No lens should be worn when sleeping. Typical organisms in corneal ulcers from contact lens wearers include staph aureus and pseudomonas. This is a potentially eye threatening condition and should be seen by an ophthalmologist the same day. These get cultured and begun on fortified broad-spectrum eye drops every hour. The contact lens, case and all solutions, need to be thrown away after they too have been cultured.
19. This is a 40 yo man who began to notice a moderately painful and reddened eye last night a little while after smoking methamphetamine (meth). The eye hurts much worse today, and he is light sensitive. On exam, his vision is 20/400 with a near card. He has a diffusely injected red eye, purulent discharge, and a sizable cloudy area on the cornea. Once again, this is a corneal ulcer, an infiltrate of the corneal stroma and an overlying corneal epithelial defect. Many corneal ulcers are associated with contact lens use. Other causes are associated with the use of smoked meth. As yet, we don’t know whether this is because of corneal anesthesia or a direct toxic effect of the smoke or a locally induced immune deficiency, but we sure do see a lot of these. Likely organisms include Staph, Strep, Pseudomonas, but H. flu, Moraxella and Enterococcus have also not infrequently been grown on cultures. Once again, these need same day ophtho referral and treatment with fortified broad-spectrum topical antibiotics. There’s a real danger of perforation of the cornea here, and these patients will occasionally lose not only corneal clarity, but the whole eye. In cases of poor compliance, these patients should be admitted. By the way, closely related to meth-induced ulcers are proparacaine induced ulcers. Patient have been known to melt their cornea by swiping the topical anesthetic from an ER.
20. This is a 60 yo woman status post cerebrovascular accident with a fluctuating mental status transferred over from a nursing home. As you assess her eyes, you notice that her they are red, and, despite the fact that she’s apparently asleep, her left eye is open about 5mm. There is a big, cloudy defect in the cornea. This is an exposure-related corneal ulcer and is a result of the eye not being lubricated and debrided by the normal blink reflex. Corneal exposure is a very common inpatient call, and it is a significant cause of ICU-related morbidity. These can be impossibly difficult to treat once they’ve begun because of the absence of the eye’s normal protective barriers. As with any ulcer, these need same day referral, culture and antibiotics. Many of these will turn out to be sterile, but they can be secondarily infected. Eventually, once sterile, they need some sort of occlusion. This can be with generous application of ointment, with a protective moisture chamber, or with a surgical tarsorraphy. (Or all of the above.) The best way to treat these is preventatively. If your ICU or chronic care patient is asleep with his or her eyes open, order a little Lacrilube ointment and apply generously to both eyes qid (four times a day). If the eyes are wide open or already starting to lose corneal luster, get an eye consult before there’s a frank ulcer. Temporary surgical closure of the lids can be discussed.
21. This is a 55 yo custodian who was splashed in the face with cleaning fluid. Both eyes hurt, and he complains of blurry vision. On exam he’s 20/70 in both eyes, and both eyes are red. The conjunctiva and sclera around the cornea in the left eye are white and avascular, and that’s a sign of a bad alkali burn. The key to managing these is copious irrigation first. Hook up a bag of normal saline to IV tubing and irrigate with 1 to 2L immediately. Lift up the lids and irrigate under them to wash out residual particles. Obvious particles that don’t wash away should be swept with a cotton applicator. After about 30 minutes, wait about 5 minutes for equilibration and check the pH of the tear fluid in the lower fornix. Urine dipsticks are perfectly OK for checking pH. If the pH isn’t 7, you need to continue irrigation for as long as it takes. Alkali is the worst thing to get splashed with, but it usually takes hours to figure out what was in the stuff the patient was splashed with. Irrigate first, ask questions later. Once pH is neutral, these get patients cyclopleged with an agent like scopolamine and lubricated with a topical antibiotic like erythromycin ointment. In cases like this, where the entire corneal epithelium is missing and the damage is extensive, the patient will need an eye doctor to see them right away. These patients typically have a long haul ahead of them and it can often take weeks before we know if and how well the cornea will heal.
22. This is a 32 yo man with a painful, watery, red right eye for the past few days. The patient tells you that light hurts his eye, and vision is decreased. The left eye is completely unbothered. Further history reveals that the patient gets cold sores when stressed. On exam, you see a red right eye and some cloudiness of the cornea. You find a fluorescein strip and dip it into the lower lid sac. When you look with a blue light, you see a branching lesion in the central cornea, with rounded bulbs on the end of the branches. This is a typical herpes simplex virus (HSV) lesion. They can occur with primary herpes infection, but they more typically show up with reactivation, and there often isn’t much of a skin rash to help out. Without a fluorescein strip, all you have to go on is the history. From the fact that there is a cloudy cornea and decreased vision, this isn’t just conjunctivitis. Treatment is with oral or topical antivirals and a cycloplegic agent. If you suspect this is primary HSV (i.e., if the patient has the typical skin rash, adenopathy and flu symptoms), they should definitely get a full course of oral acyclovir or valacyclovir. Undiagnosed HSV is one of the big reasons non-ophthalmologists should avoid prescribing topical steroids. If HSV is like fire, steroid eye drops are like gasoline.

1. This 65 yo woman has had a worsening red left eye and left sided facial rash over the past few days. Her vision is decreased, and she complains of terrible light sensitivity. This is the typical history and appearance of HZO, or herpes zoster ophthalmicus. Generally, eye symptoms are accompanied by a unilateral cranial nerve 5 dermatomal rash. Patients are usually over 60, and if they’re under 40, they should receive a workup for immunocompromise. Zoster can do almost anything to the eye. Affecting every structure from lid, to cornea, to extraocular muscles, to retina. Intraocular involvement can lead to secondary increases in intraocular pressure and glaucoma. Patients with zoster lesions get high dose oral valacyclovir or acyclovir, erythromycin or bacitracin ointment, and cool compresses to the skin lesions. Ocular involvement frequently requires topical steroids, which should be ordered by their eye doctor.
2. This is a 45 yo woman complaining of bilateral photophobia and decreased vision in both eyes for about a week. She doesn’t recall a previous episode. She’s had increasing shortness of breath and fatigue recently, and her PCP has her scheduled for an outpatient chest X-ray. On eye exam, you notice that the eye is injected. The cornea has big brown dots on it. This is an eye with sarcoid. 25% of sarcoid cases involve the eye in some way, and a fair amount of otherwise asymptomatic sarcoid presents as eye disease. Specifically, sarcoid causes a granulomatous inflammation of the iris, ciliary body and the choroid (i.e., the pigmented tissues making up the middle layer or uvea of the eye). This is an iridocyclitis or uveitis. Ocular sarcoid is difficult to treat and patients not infrequently go blind from the secondary increase in eye pressure and glaucoma. The mainstay of treatment is topical, periocular and systemic steroids. Ocular tuberculosis and ocular syphilis can also look just like this. The workup for this finding usually includes a PPD, RPR, chest X-ray and a medicine or rheum consult.
3. This is a young man who presents with unilateral photosensitivity for the past two days. Vision is uncompromised. Review of systems elucidates back stiffness. On exam, the eye is diffusely injected with dilated blood vessels coming right up to the corneal margin. There is no discharge or corneal cloudiness, and the conjunctiva inside the lids don’t seem especially inflamed. This is an iritis, or an inflammation of the iris and surrounding tissues. The majority of times these are unilateral, nonrecurrent and idiopathic. The critical signs are red eye and photophobia (exquisite light sensitivity) without signs of infection. Topical anesthetic does not relieve the discomfort the way it would in a conjunctivitis or corneal abrasion. These get treated with topical steroids beginning at every one or two hours and tapering from there. Recurrences need to get worked up. In particular, history of rashes, arthritis, back pain, or bowel disease should be taken as strong reason to suggest HLA-B27 related diseases such as ankylosing spondylitis, Reiter’s syndrome, or inflammatory bowel disease. Get a sacroiliac film and a medicine consult after a second attack, as early physical therapy can often prevent disability. This is uveitis.
4. This is a 60 yo woman with a recent complaint of increasing pain in her eye that wakes her from sleep. The pain is boring and worse with eye movement or if the eye is touched. She has a history of sinus trouble, and recently, she’s been having increasing cough and shortness of breath. On exam you see an eye with large and deep injection. Areas underlying the conjunctiva look bright red, slate gray, or black. This is a scleritis in a patient with Wegener’s granulomatosis. 60% of Wegener’s pts will have eye involvement, and a fair amount of Wegener’s presents with eye complaints or isn’t recognized until the eye symptoms occur. Scleritis is an inflammation of the sclera, the tough outer coating of the eye. Signs & symptoms include severe pain, decreased vision, large dilated blood vessels that are deep to the conjunctiva and so don’t move with a cotton swab, and a thinning of the sclera resulting in a blue-gray appearance. 50% of patients with scleritis have a serious associated systemic disease such as Wegener’s, lupus, rheumatoid arthritis, syphilis, tuberculosis or sarcoid. The five-year survival after scleritis diagnosis is 30%. Treatment usually involves treatment of the underlying condition and immunomodulation with systemic NSAIDS, steroids or cyclophosphamide.
5. This is a 52 yo woman with a sandy/gritty/burning feeling in both eyes for months. Her vision is unaffected. On exam you notice a rash on the cheeks, forehead and nose, with some pustules and lots of fine broken blood vessels. Her conjunctiva is mildly injected as well. This is a patient with acne rosacea, a skin condition which probably presents as eye irritation more than skin complaints because most patients just assume that they’re skin is supposed to look that way. The telangiectatic rash in this distribution is typical. Pathophysiology is unclear. The eye complaints are related to plugging and inflammation of the oil glands at the margins of the eyelids. This causes both direct eye irritation as well as dry eye from tear deficiency. When asked, patients will often admit to facial flushing with spicy foods. The mainstay of treatment for the eye complaints are warm compresses and lid scrubs to unplug the oil glands. Oral doxycycline starting at 100 mg twice a day and tapering to the lowest dose necessary will change oil composition and can relieve both eye and skin symptoms.
6. This is a 36 yo woman complaining of a sandy, gritty, burning sensation in both eyes for months. She sees a something growing in her eye, and she’s afraid she has cancer. Her vision is normal. On exam, you see a wedge or wing shaped area of injected vessels that appears to be growing toward and onto the cornea. This is a pterygium, or a benign conjunctival degeneration of the conjunctiva. It’s called a pterygium because of its characteristic winged appearance. They’re usually temporal and bilateral, but people can have any combination of nasal and temporal lesions. They seem to be more common in Hispanic and Asian populations and seem also to be related to sun exposure. Occasionally, they become inflamed by smoke or allergens. The treatment is identical to allergic conjunctivitis: cool compresses, artificial tears and perhaps topical NSAIDS like ketorolac (Acular) if necessary. These can be removed surgically, but because of a high regrowth rate (about 30%), they are typically left alone unless they’re encroaching on the visual axis or stretching the eye.
7. This is a fairly rare cause of red eye, but it is commonly board-tested. This is a 40 yo woman with severe unilateral eye pain since this morning. Her vision has been getting progressively worse over the morning and she sees halos around lights. She admits to occasional bouts with similar pain while driving at night or in a movie theater. On exam, you see diffuse injection of both deep and superficial blood vessels and a diffusely cloudy cornea. This is acute glaucoma. It is easily distinguished from benign causes of red eye by the severe discomfort and decreased vision. It is caused by anatomic changes in the eye that block the outflow of aqueous humor and usually occurs in patients with a narrow (or acute) angle between the cornea and the iris where the drainage system lives. Initial treatment involves lowering the pressure by whatever means possible. Throw on a beta blocker eye drop, some Brimonidine (Alphagan), and give some oral (or IV) acetazolamide (Diamox). If after 20 minutes the pressure is still high, consider oral glycerin (Osmoglyn) or IV mannitol. When these work, they’ll buy you up to half a day. Whether or not you manage to break the attack medically, you are going to need find an eye doctor who can perform an iridotomy (create a hole in the iris) to re-establish flow from the posterior chamber to the anterior chamber of the eye. It is worth mentioning that it’s OK to give glaucoma patients all those meds like decongestants that carry a warning about glaucoma. The warning only concerns un-recognized acute angle glaucoma. Anyone who knows they have glaucoma either has the usual kind (primary open angle glaucoma) or they’ve generally been treated with laser and are safe.
8. A 70 yo woman presents to the ED with pressure to the frontal region of her head and states she's also had a runny nose with tearing and redness of her left eye. She denies any eye pain or visual changes. Her vision is 20/400 but her baseline is unknown. She has a history of macular degeneration of the left eye and states she had a shot in this eye last week. This is an infection secondary to Avastin injection. Patients are told to call for RSVP – Redness, Sensitivity to light, Vision loss, or Pain – after injections. On the one hand, this is a very rare cause of intraocular infection with an incidence of about 1:1000 or less. On the other hand, injections are very common. This is endophthalmitis with hypopyon.
9. Before moving away from red eye, here are some do’s and dont’s. Topical anesthetic, like proparacaine, is useful in the office, both to cool off a hot eye to allow you to examine it and to help distinguish surface irritation from a deeper problem, but don’t ever give it to a patient for use at home. Used chronically, its directly toxic to the corneal epithelium and actually prevents healing. Topical steroids are extremely effective for treating some types of ocular inflammation. Routine lid and conjunctival disease do not require topical steroids, which will put the patient at risk for a serious eye infection. If you think your patient needs topical steroids, give the ophthalmologist a call. A lot of patients and some doctors feel that an eye patch provides good symptomatic relief for eye irritation. It’s still the treatment of choice for large, clean corneal abrasions. But never patch any eye you suspect is infected. The patch will turn the eye into a culture medium. If your conjunctivitis antibiotic doesn’t appear to be working, you might have a resistant bug. More likely, though, your patient has developed a drug reaction. If there are no signs of infection, try stopping the drug. Finally, the examples demonstrate the few things that distinguish benign conditions like conjunctivitis from serious conditions that require urgent referral are severe pain, decreased vision, and cloudy eyes.
10. Goal number three: evaluation of the traumatized eye. There are just a few guiding principles here.
11. The first and most important thing to remember is do not press on the eye!! If a patient has a corneal or scleral laceration and you press on the eye, you cannot charge them for a cataract procedure just because you’ve watched the lens dribble down the face. The things that will probably tell you most quickly how serious things are likely to be are: 1) the history (i.e., mechanism of injury) and 2) how bad the patient’s vision is. The EOM exam will quite frequently show deficits. It’s often difficult to know, however, whether those deficits are paretic or restrictive. A through & through lid laceration, cornea or conjunctival laceration, blood in the anterior chamber, or a distorted/teardrop-shaped pupil are all good reasons to suspect a perforated eye, or ruptured globe. If you have any suspicion you see these then don't press on the eye!
12. A 64 yo man who was playing with his grandkid was poked in the eye. The eye is red, stings, and is mildly photophobic. Near vision is down to 20/30 in the affected eye. On exam, the eye is mildly injected but doesn’t appear to have any lacerations. Anesthetizing the eye with proparacaine (Ophthetic) relieves the patient’s pain, quickly demonstrating that this pain localizes to the ocular surface. Fluorescein dipped gently into the inferior lid sac and exam with a blue light demonstrates a sharply defined are of blue-green fluorescence. This is a corneal abrasion. The fluorescent area corresponds to the area where the corneal epithelium has been scraped off and the fluorescein is adhering to the exposed collagen. Treatment consists of cycloplegia with a medication like cyclopentolate or scopolamine and lubrication with an antibiotic ointment such as erythromycin four times a day for about 5 days. Patching doesn’t make the eye heal more quickly, and a recent study showed that most patients are more comfortable without the patch. Abrasions are usually completely healed in 24 to 48 hours.
13. A 35 yo construction worker, filing some metal without goggles on, felt something fly into his eye. 2 days later, the eye still feels like there’s something in it, and the vision is a little blurry. On exam, the vision is 20/30 with a near card. The eye is mildly injected and there’s a 2mm brown spot off center on the cornea. This is the appearance of a metallic foreign body. Unless you catch them in the first few hours, you’re as likely to see this rust ring as you are to see something shiny and metal. Relief and rapid healing follow removal of the rusty metal. If something is really superficial, you may be able to wipe it off the cornea with a cotton swab. More than likely, though, removal will require magnification and either a spatula, a burr, or a bent 25-gauge needle. Once the majority of the metal and rust are removed, the eye is treated like a corneal abrasion, with an antibiotic ointment such as bacitracin or erythromycin ointment four times a day for a few days. A Tetanus booster should be given if indicated.
14. This is an 18 yo man whose friend got wild with a BB gun. The BB ricocheted off a truck and then off the patient’s left eye. The patient complains of a sore eye and decreased vision. On exam, vision is decreased to 20/400 at near. You see areas of subconjunctival hemorrhage and what appears to be blood behind the cornea in the anterior chamber. This is an example of a hyphema, blood in the anterior chamber of the eye. It’s also an example of an eye you really don’t want to be the one to press on. An injury like this could easily hide a small corneal or scleral laceration or an entrance wound for an intraocular foreign body. This patient needs a careful pupil exam to check for an afferent pupillary defect (APD). He needs a careful slit lamp exam, a gentle intraocular pressure check, and a dilated eye exam. If it turns out the patient’s eye is intact, treatment consists of bed rest with elevated head to let the blood settle and careful daily monitoring for an increase in intraocular pressure due to blood products clogging the aqueous drainage system. Watch out for the patients with sickle-cell because they are at greater risk from elevated eye pressure.
15. To round off the trauma section, this is a 45 yo homeless man who was minding his own business when he was kicked in the face with a steel-tipped boot. The eye is quite painful, and vision is down. On exam you find vision to be LP, light perception only, and there appears to be stuff coming out of the eye. This is a ruptured globe. The stuff coming out of the eye is iris and/or retina and this gentleman needs to go to the OR for surgical repair. Visual prognosis in a case that looks this bad from the start is probably pretty poor but occasionally an eye like this maintains useful vision. This is another, more subtle example, showing the classic “peaked” pupil that is seen when the iris decides to plug a full-thickness corneal laceration. This patient, like the previous patient, is at risk for a devastating infection inside the eye, if its structural integrity is not restored emergently. If you have the misfortune to see one of these, please remember the next slide:
16. Don’t press on the eye. Call for help. Don't patch the eye. Positive pressure from the patch may be all that's needed to extrude the remainder of the eye contents. Instead, use a Fox shield, one of those metal shields you sometimes see over an eye patch. If there isn’t a shield around, use a plastic or paper drinking cup. If there’s any suspicion of a foreign body, you can speed things up enormously by ordering a CT of the orbits. The surgical plan for an eye with an intraocular foreign body is drastically different, requires a different set of instruments and usually a different surgeon. Feel free to start an IV fluoroquinolone if feasible, but not at the expense of delaying the patient’s arrival to the hospital where the repair will be done. Reminding the patient to stay NPO is of course helpful, as is updating his tetanus shot. And finally, don’t press on the eye!
17. OK, onto the next goal: detecting abnormal eye movements
18. This is a 28 yo man in for a routine checkup. He has no eye complaints. On exam he seems healthy, but his vision is 20/20 in the left eye and 20/200 in the right eye, and his eyes, while they appear to have full range of movement, are crossed when he looks straight ahead. This is a man with an uncorrected congenital esotropia. That is, his eyes have been crossed pretty much from the first year of his life. There are quite a few possible etiologies including a big difference in refractive error between the two eyes or even just very high farsightedness in both eyes, but the result is that the patient begins to favor one eye over the other. Eventually neural connections are formed preferentially in one eye, and the other eye becomes lazy or amblyopic despite being anatomically healthy. In most cases, amblyopia not corrected by age 7-9 is uncorrectable. Treatment requires early eyeglass correction, often diligent patching, and occasionally even surgical correction of the misalignment. This young man does not have double vision as his brain essentially turns the left eye off as long as the right eye is looking at something. While we can’t correct this patient’s visual acuity at this age, it is however feasible to surgically correct the crossing for psychosocial reasons.
19. This is a 63 yo diabetic, hypertensive gentleman who reports sudden onset of droopy right eyelid and double vision whenever he picks the lid up. The vision from either eye appears normal. On exam you note complete ptosis of the right upper lid. Pupil exam is normal, but extraocular movement (EOM) exam shows that the right eye cannot turn up, down, or in. Lateral movement in that eye is normal, and there are no deficits in the right eye. This is, of course, a third nerve palsy. You’ll remember that cranial nerve (CN)III controls the inferior, medial, and superior recti as well as the levator muscle of the upper eyelid. CNIII also carries pupillary fibers over part of its path. The majority of isolated CNIII palsies that spare the pupil are simply due to microvascular disease. CNIII function usually recovers spontaneously over a period of three to six months and the patient can use a patch over one eye if the double vision is bothersome. In this very specific circumstance, no further workup is needed (except maybe an ESR and CRP to rule out temporal arteritis). But since an MRI is so easy to get these days, one is often ordered for CNIII palsy.
20. The majority of pupil-involving CNIII palsies represent something more serious, such as an aneurysm or a pituitary mass. Any presentation of an eye movement disorder not attributable to an isolated, pupil-sparing third nerve palsy also needs more extensive workup. Our workup in the setting of sudden EOM paresis involves careful exam of all other cranial nerves. MRI is urgently indicated if the pupil is involved, the age is less than fifty, there are other CN anomalies, or the paresis is incomplete—that is, some muscle function is spared. Always consider an ESR and CRP in an older patient with sudden onset of EOM deficit. Temporal arteritis can as easily affect the vessels supplying the extraocular muscle cranial nerves as it can the vessels supplying the optic nerve.
21. This is a 54 yo woman with gradually worsening double and blurry vision over the past few months. She’s been feeling rather anxious and tremulous lately. On exam you find her vision is down to 20/100 in the right eye and 20/30 in the left. Her pupil exam demonstrates an afferent pupillary defect (APD) or Marcus-Gunn pupil in the right eye. Her EOM exam shows restriction of medial and downward gaze in the right eye and bilateral limitation of upgaze. She has a palpable rubbery neck mass. This is of course thyroid ophthalmopathy. It’s easy to remember that Graves’ disease causes this typical bug-eyed appearance, as the extraocular muscles are involved. It’s also important to remember that the extraocular muscle enlargement can cause terrible restriction of eye movement and double vision. The tissue in the orbit can actually become enlarged enough to press on the optic nerve and strangle it and cause decreased vision, as in this case. The often referred to and misunderstood lagophthalmos in Graves’ is seen by asking the patient to close their eyes lightly as if they were sleeping. The lid lag is the measurement of the amount the eyelids remain open. A way to quickly recognize the proptosis, or exophthalmos, in this patient is your ability to see the sclera above the cornea when the patient looks straight ahead. This is Graves’ Ophthalmopathy.
22. Last in the eye movement gallery, this is a 63 yo woman with gradually worsening double vision and no other associated symptoms. On exam, you find vision slightly down on the left. Pupil reaction seems normal but there’s significant restriction of lateral and downward gaze. In addition, the eye looks red with big blood vessels coming right up to the cornea. Notice the little bit of sclera showing superiorly telling you that the eye is proptotic. This is a carotid artery-cavernous sinus fistula. These occur in young folks secondary to trauma or in older folks, usually women, for no good reason. The spontaneous fistulae in the older population tend to be communication of dural vessels with the sinus and less severe than the fistulas in trauma cases. The danger in either case is that the pressure on the eye from behind can raise intraocular pressure causing glaucomatous nerve damage and choking circulation to the eye. Workup includes a carotid angiogram which is sometimes sufficient to cause closure of the communications.
23. Next, detection of abnormal pupils.
24. Before looking at two scenarios, here’s a few pupil pointers. The most important thing to look for in the pupil exam is a Marcus-Gunn pupil, or afferent pupillary defect (APD). It’s much more important than knowing that the pupils are equal and constrict equally to light. It tells you about the health of the eye’s retina and optic nerve. Anisocoria of less than 1mm is present in up to 20% of the population at any given time. Post-surgical pupils and pupils of diabetics may not constrict at all, but the eye may see perfectly well. Pupils are best examined in a slightly dimmed room. If you really don’t have time to examine the pupils, then don’t write PERRLA. An abnormal pupil exam becomes much scarier when earlier documentation says the pupils were normal.
25. This is the first of two abnormal pupil schematics. In both cases, the scenario is a young person hit in the face with a fist. There’s no change in mental status. In this case, pupils are 5mm in a dim room. A penlight shown in the right eye causes constriction of the right eye but no apparent consensual response. A penlight swing over to the left eye shows no subsequent change in the pupils. How serious an eye injury is this? The answer is, probably not very. The key here is that the pupil on the right does not come back open when the light is swing over to the left. That is the direct & consensual responses of both eyes are equal. Both eyes are detecting light equally well. The lack of constriction of the pupil in the left eye could easily be due to a temporary paresis of the iris sphincter muscles. But it doesn't indicate that the seeing part of the eye—the retina and optic nerve—are damaged.
26. Here’s the contrasting situation. Again, both eyes are 5mm in a dim room. When light shines in the right eye, both pupils constrict to 2mm. When the light is swung quickly to the left eye both pupils now dilate to 3mm. How serious is this injury? Pretty bad. Both the direct and consensual response is weaker when light is shown in the left eye than when it’s shown in the right. This is a Marcus-Gunn pupil or afferent pupillary defect and tells you that the retina or optic nerve of the left eye in this case is very sick. This patient needs an eye exam and probably an orbital CT now. By the way, you can’t get an APD from eye opacities such as cloudy corneas, cataracts or blood in the eye. The reason for this is debatable, but you can’t.
27. I have no caselets to show you for the perform direct ophthalmoscopy goal, but keep in mind just a few things.
28. Direct ophthalmoscopy is the one place in the whole body where you can directly observe your patients’ arteries, veins, and nerves without cutting them open. But it’s a difficult thing to do in an undilated eye, especially if you haven’t gotten practice by starting with dilated eyes. It’s OK to dilate. Keep a bottle of 2.5% phenylephrine and put the drops in right after checking pupils. The eyes will be dilated by the end of the exam. Some doctors express concern that they’re afraid of accidentally dilating a patient with glaucoma, but dilation can only induce a pressure attack in patients with previously undiagnosed acute angle glaucoma. This is somewhat less that 1 in 1000 patients. Honestly, the more practice attained through a dilated pupil the easier it will be to find landmarks and really seeing just how badly the patients’ diabetes or hypertension is affecting their microvasculature.
29. Finally, the AUPO wants you to be proficient in is initiating management or referral. As we’ve just spent the last 50 slides discussing management, just a few words about referral. Please, please remember the difference between an optometrist and an ophthalmologist. An optometrist’s primary training is in the prescription of corrective lenses. Due to extensive lobbying efforts optometrists now have the right to prescribe certain medications but optometrists have not gone to medical school, been through a residency, and are not MDs. If your patient has eye disease, please for your sake and theirs, please refer them to an ophthalmologist.
30. Thanks for your attention in covering this presentation’s objectives. Hopefully you have learned something about ophthalmology that will make you feel more comfortable handling ophthalmologic complaints. Do not hesitate to refer to an ophthalmologist for management of eye disease or for eye emergencies. Any questions?
