# Supplementary material for: A Required Ophthalmology Rotation: Providing Medical Students with a Foundation in Eye-Related Diagnoses and Management
Source: MedEdPORTAL. 2021 Feb 12;17:11100. doi: 10.15766/mep_2374-8265.11100 (PMC7880261; doi:10.15766/mep_2374-8265.11100)
Supplement: Supplementary file 1 — Ophthalmology Slides Instructors Guide.docxOphthalmology Handout.docxOphthalmology Slides.pptxOphthalmology Sessions.docxOphthalmology Sessions Answer Key.docxOphthalmology Sessions Student Handouts.docxOphthalmology Final Examination.docxStudent Postrotation Feedback Form.docx [file mep_2374-8265.11100-s001.zip › B. Ophthalmology Handout.docx]

**Ophthalmology (Eye Stuff) Made Easy**

***1) Measure and Record Visual Acuity***

**Visual Acuity** - Top number refers to distance measured in feet. Bottom number refers to distance at which “normal” eye could make out same figures.

**Landmarks on Visual Acuity Chart**

20/15 Supernormal

20/20 Normal

20/40 DMV’s* minimum for driving

20/200 Big E on chart, legal blindness

CF Can count fingers

LP Can detect light

NLP Cannot detect light

**Visual Acuity Tips and Tricks**

- Always record how taken - near or far
- Always use patient’s correction, if possible
- Always measure each eye separately
- Check for refractive problem by checking at different distances or by rechecking with pinhole

**Visual Acuity Terms to Know**

- Nearsighted, Myopic
- Farsighted, Hyperopic
- Emmetropic
- Astigmatism
- Presbyopic
- Legally Blind
- Telangiectasia
- Infiltrate
- Epithelial Defect
- Dendrite
- Synechia
- Keratic Precipitate

***2) Evaluate a Red Eye***

**Red Eye Signs**

**Red Eye Do’s and Don’ts**

- Injection
- Chemosis
- Papillary Reaction
- Preauricular Node
- Ciliary Flush
- Cell and Flare
- Never prescribe topical anesthetic
- Leave topical steroids to ophthalmologists
- Never patch pus
- If red eye persists or returns after Rx, consider drug reaction
- Refer cases with decreased vision
- Refer cases with cloudy eyes
- Refer cases with pain

**Selected Red Eye Diagnoses**

- Acne Rosacea
- Iritis, Iridocyclitis, Uveitis
- Scleritis
- Narrow angle glaucoma
- Corneal Ulcer
- Alkali Burn
- Herpes Simplex
- Herpes Zoster (Shingles)
- Pterygium
- Conjunctivitis
  - Allergic
  - Bacterial
  - Viral
- Subconjunctival Hemorrhage

1. ***Evaluate a Traumatized Eye***

**Ruptured Globe Do’s and Don’ts**

**Trauma Do’s and Don’ts**

- Don’t press on the eye!
- Evaluate mechanism
- Check vision
- Fox shield, don’t patch
- Look at lids, conjunctiva, cornea, anterior chamber
- Look closely at pupils
- Don’t press on the eye!
- Don’t press on the eye!
- Emergent eye consult
- Fox shield, don’t patch
- CT orbit, if suspect intraocular foreign body
- IV antibiotics, if feasible
- NPO
- Don’t press on the eye!

*Department of Motor Vehicles

1. ***Evaluate a Traumatized Eye (continued)* Selected Trauma Diagnoses**

- Corneal Abrasion
- Corneal Foreign Body
- Hyphema
- Ruptured Globe
- Intraocular Foreign Body

***4) Evaluate abnormal eye movements***

**Eye Movement Terms to Know**

**Selected Eye Movement Diagnoses**

- Strabismus
- Esotropia
- Exotropia
- Hypertropia
- Proptosis
- Diplopia
- Graves’ disease
- Cavernous-carotid fistula
- Myasthenia Gravis
- Multiple Sclerosis
- Congenital Exotropia or Esotropia
- Amblyopia (“lazy eye”)
- Third nerve paresis
- Sixth nerve paresis
- Fourth nerve paresis

**Pupil Terms and Diagnoses**

***4) Evaluate pupils***

**Pupil Do’s and Don’ts**

- Light-near dissociation
- Horner’s syndrome
- Marcus Gunn pupil
- Argyll Robinson pupil
- Third nerve palsy
- Most important sign is an APD
- 1 mm anisocoria or less may be normal
- Use a dim room
- Don’t write PERRLA if it ain’t so!


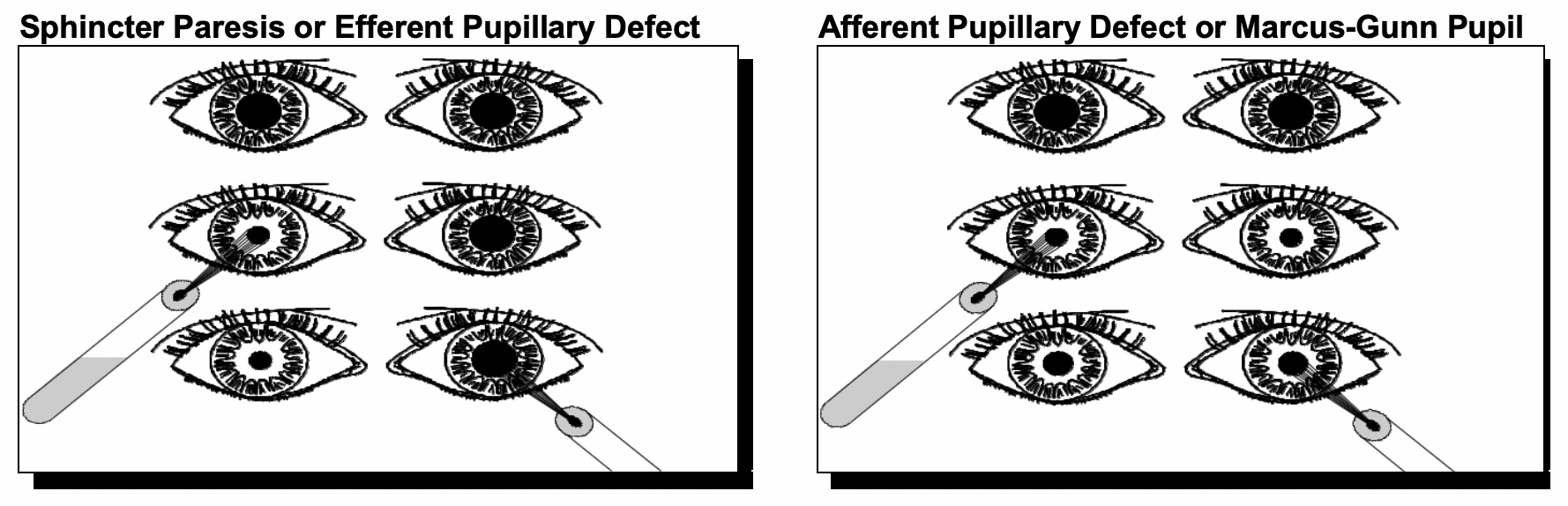


***7) Initiate Referral and Management***

***6) Perform Direct Ophthalmoscopy***

- Ophthalmologist - physician (MD) who specializes in medical and surgical care of the eyes and visual system. Training - college, med school, residency
- Optometrist - (OD) determines visual acuity, prescribes spectacles & contact lenses. Some states permit optometrists to give limited treatments of some eye conditions. Training - college, optometry school
- Optician - technician who makes, verifies and delivers lenses, frames, and contact lenses upon prescription.
- It’s okay to dilate
- Only untreated angle closure glaucoma patients are at risk for acute glaucoma attack (<1 in 1000)
- Use 2.5% phenylephrine (Neosynephrine) to avoid affecting accommodation
- Never use atropine!
- Put drops in during your HEENT exam. They’ll have worked by the time you’re done with the rest of the exam.
