# Supplementary material for: A Required Ophthalmology Rotation: Providing Medical Students with a Foundation in Eye-Related Diagnoses and Management
Source: MedEdPORTAL. 2021 Feb 12;17:11100. doi: 10.15766/mep_2374-8265.11100 (PMC7880261; doi:10.15766/mep_2374-8265.11100)
Supplement: Supplementary file 1 — Ophthalmology Slides Instructors Guide.docxOphthalmology Handout.docxOphthalmology Slides.pptxOphthalmology Sessions.docxOphthalmology Sessions Answer Key.docxOphthalmology Sessions Student Handouts.docxOphthalmology Final Examination.docxStudent Postrotation Feedback Form.docx [file mep_2374-8265.11100-s001.zip › C. Ophthalmology Slides.pptx]

## Slide 1
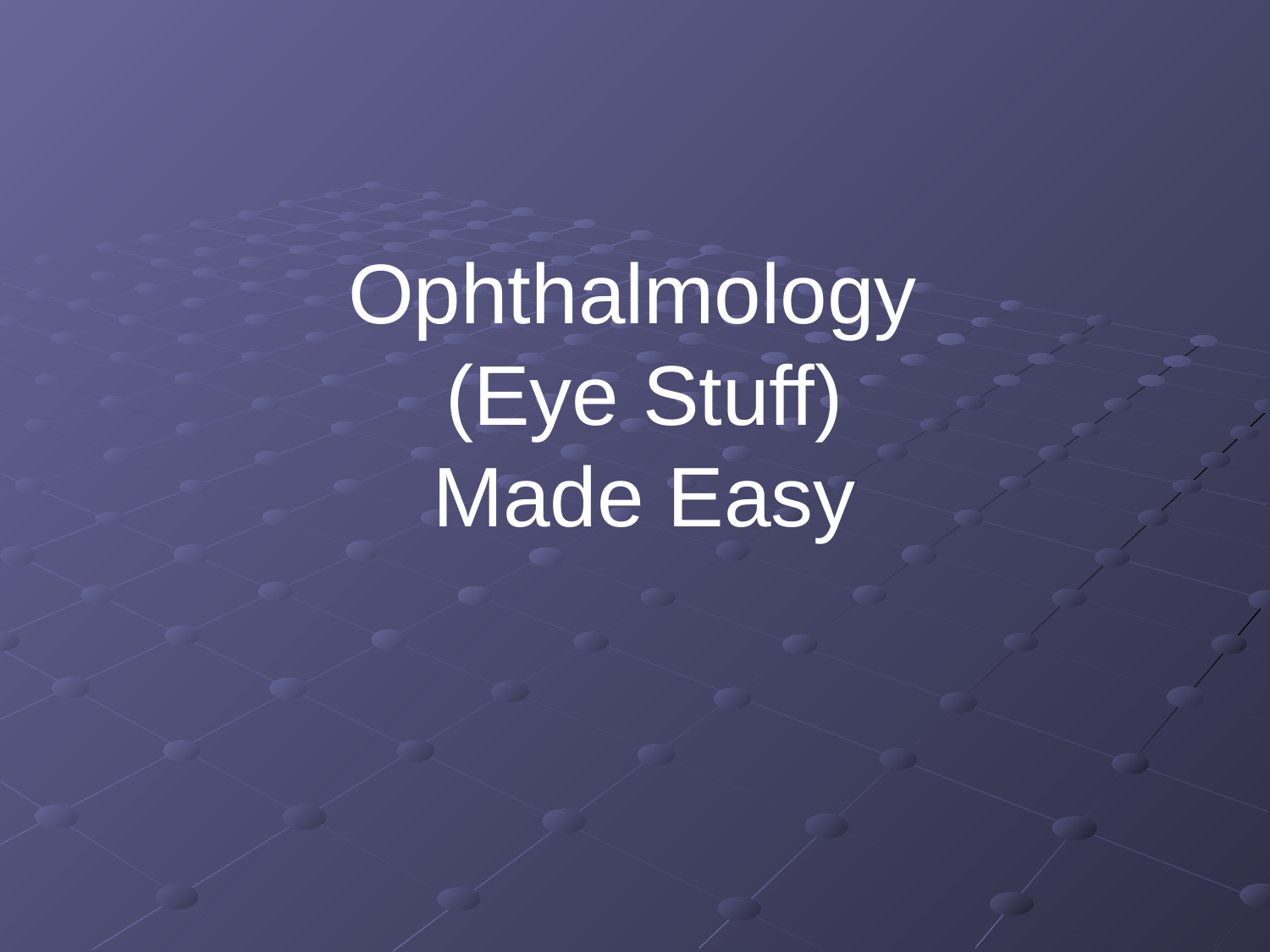

Ophthalmology
(Eye Stuff)
Made Easy

## Slide 2
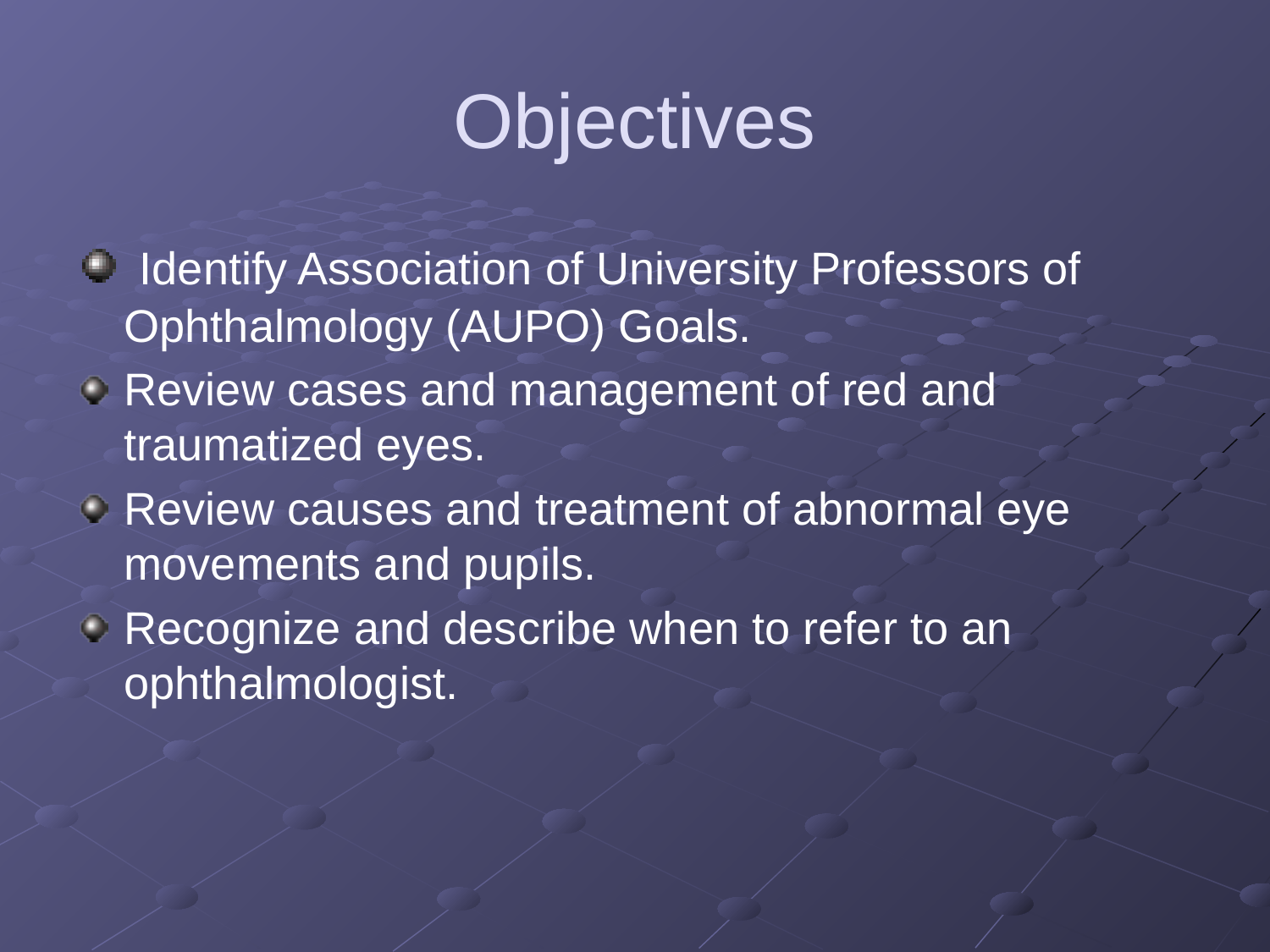

# Objectives
 Identify Association of University Professors of Ophthalmology (AUPO) Goals.
Review cases and management of red and traumatized eyes.
Review causes and treatment of abnormal eye movements and pupils.
Recognize and describe when to refer to an ophthalmologist.

## Slide 3
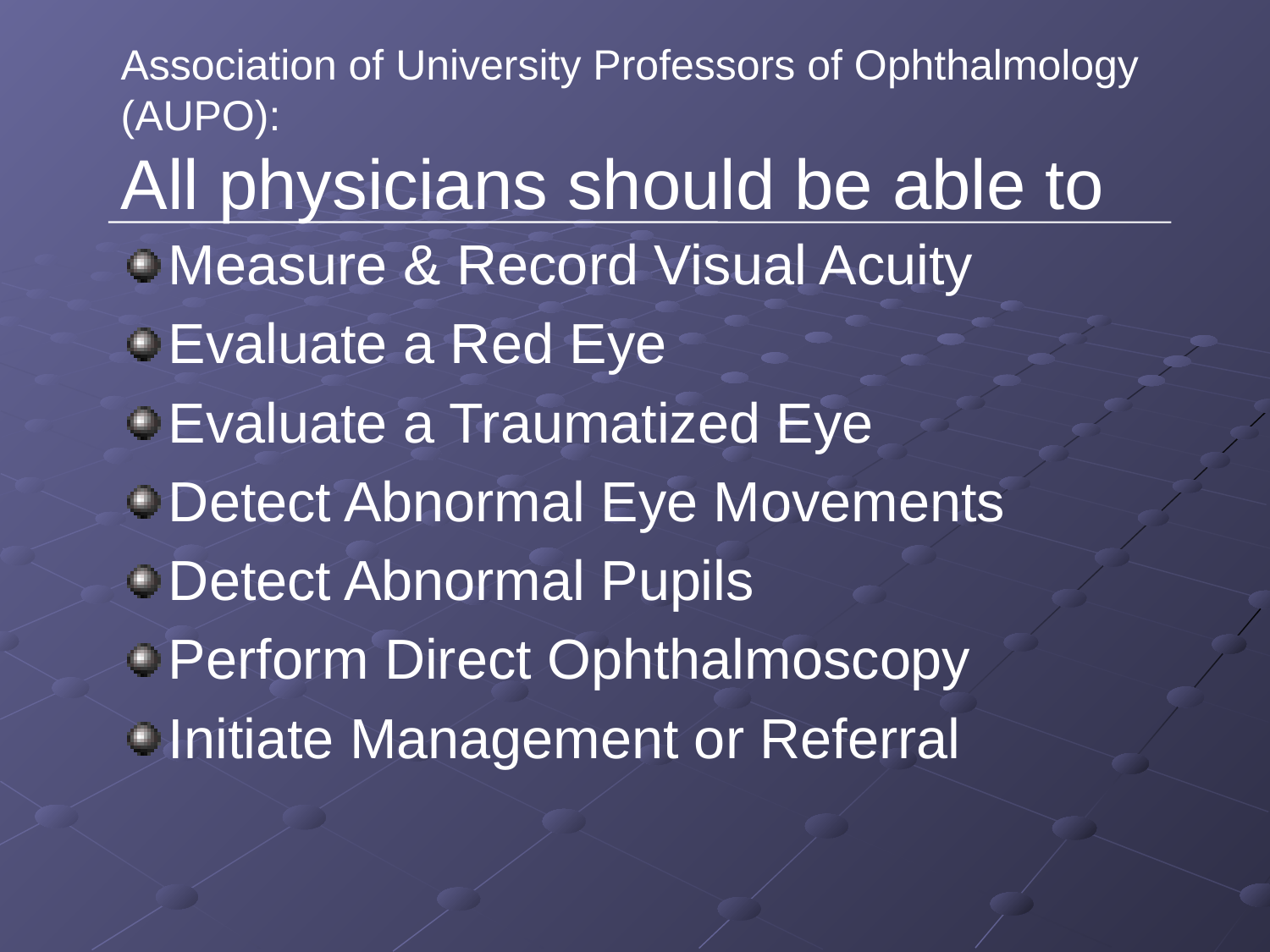

# Association of University Professors of Ophthalmology (AUPO): All physicians should be able to
Measure & Record Visual Acuity
Evaluate a Red Eye
Evaluate a Traumatized Eye
Detect Abnormal Eye Movements
Detect Abnormal Pupils
Perform Direct Ophthalmoscopy
Initiate Management or Referral

## Slide 4
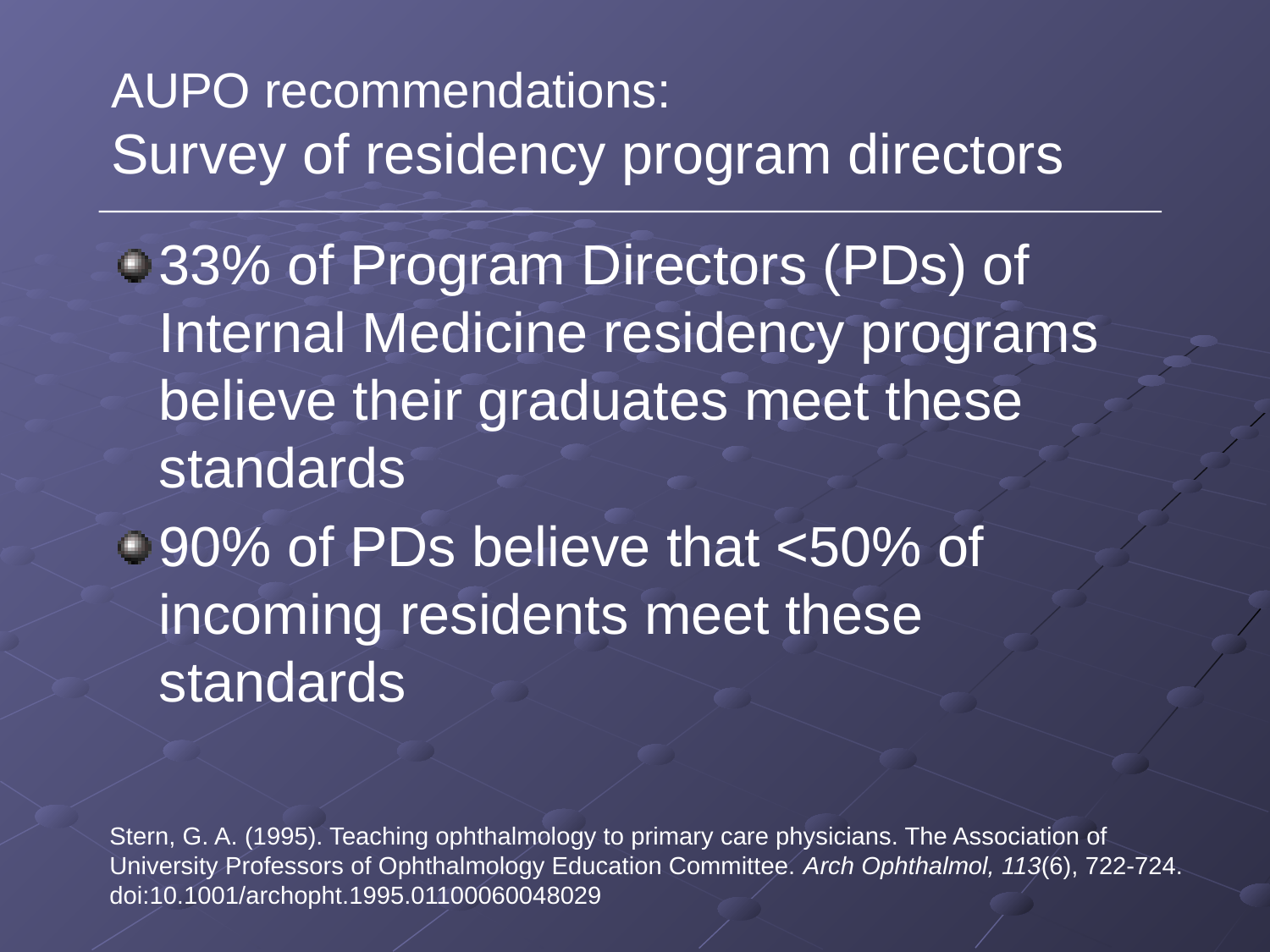

# AUPO recommendations: Survey of residency program directors
33% of Program Directors (PDs) of Internal Medicine residency programs believe their graduates meet these standards
90% of PDs believe that <50% of incoming residents meet these standards
Stern, G. A. (1995). Teaching ophthalmology to primary care physicians. The Association of University Professors of Ophthalmology Education Committee. Arch Ophthalmol, 113(6), 722-724. doi:10.1001/archopht.1995.01100060048029

## Slide 5
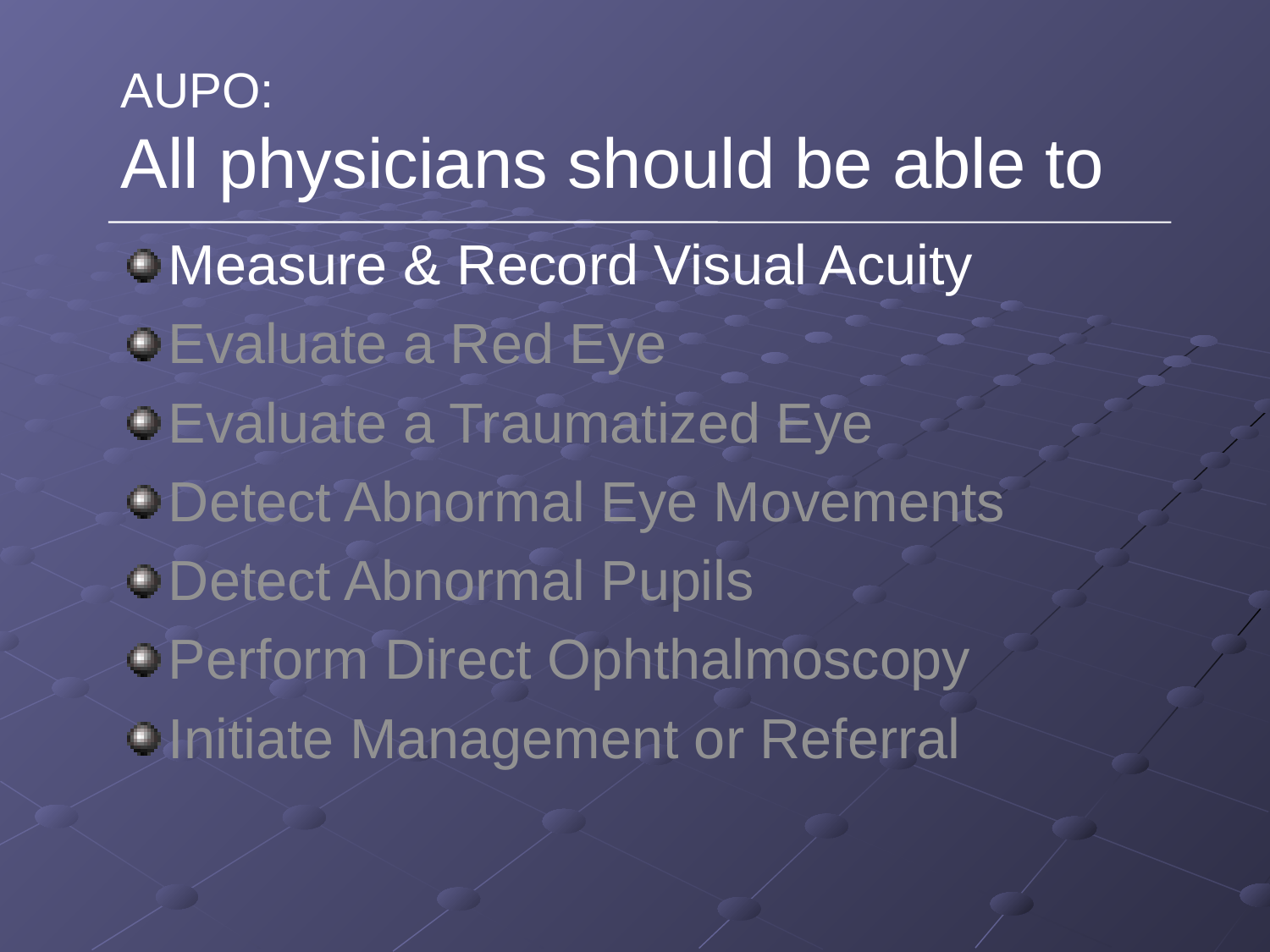

# AUPO: All physicians should be able to
Measure & Record Visual Acuity
Evaluate a Red Eye
Evaluate a Traumatized Eye
Detect Abnormal Eye Movements
Detect Abnormal Pupils
Perform Direct Ophthalmoscopy
Initiate Management or Referral

## Slide 6
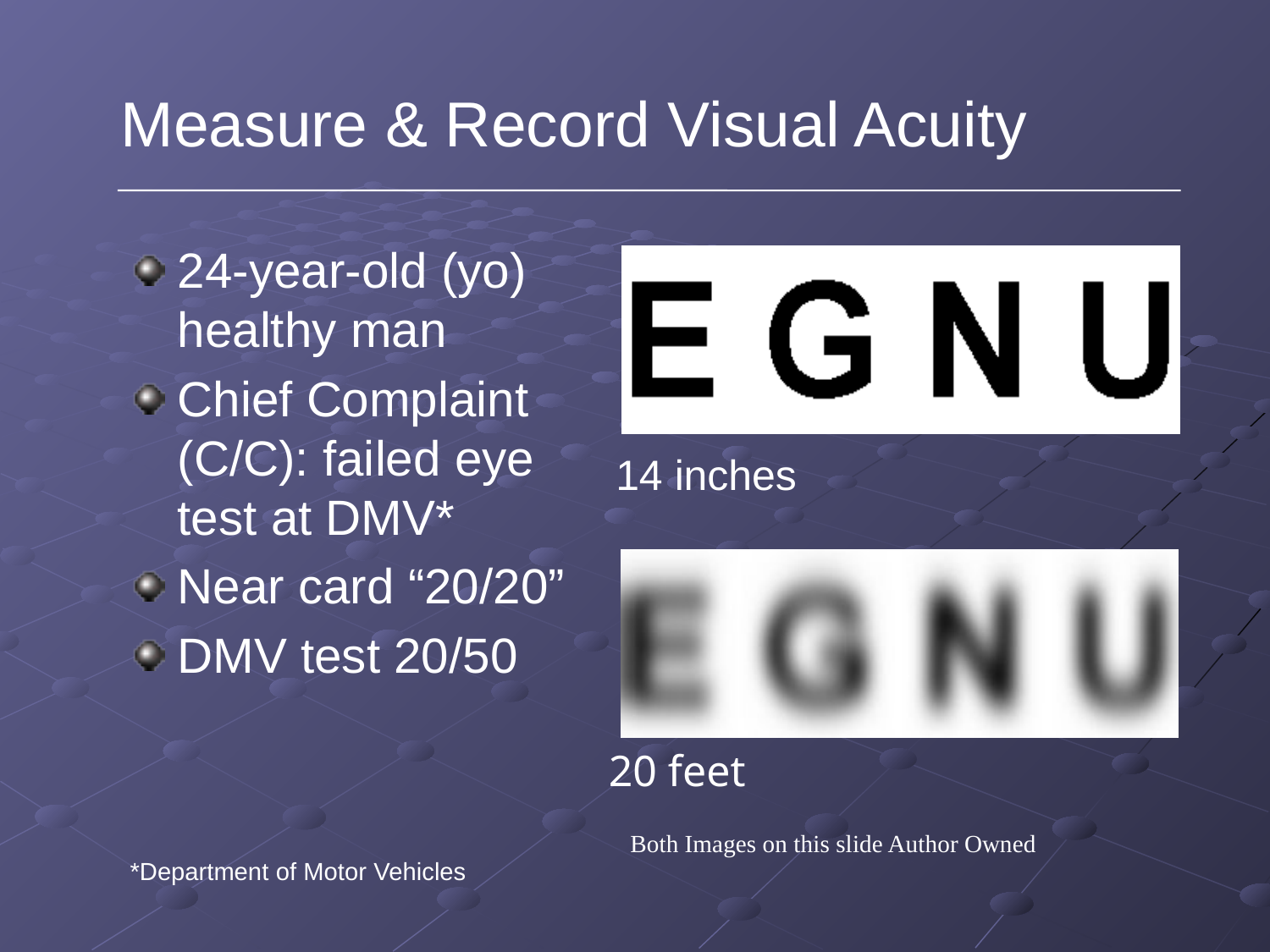

# Measure & Record Visual Acuity
24-year-old (yo)
healthy man
Chief Complaint (C/C): failed eye test at DMV*
Near card “20/20”
DMV test 20/50
14 inches
20 feet
Both Images on this slide Author Owned
*Department of Motor Vehicles

## Slide 7
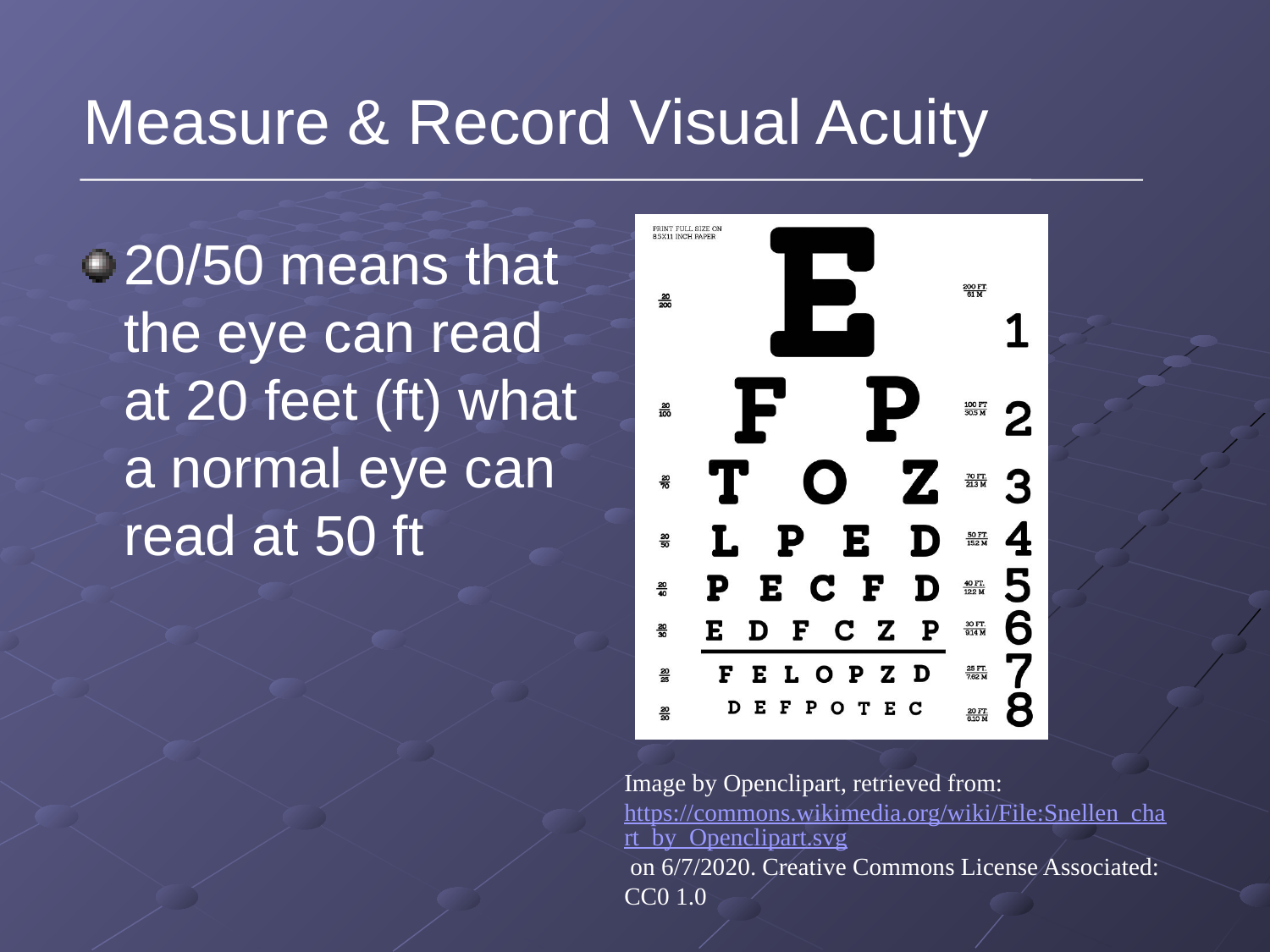

# Measure & Record Visual Acuity
20/50 means that the eye can read
at 20 feet (ft) what a normal eye can read at 50 ft
Image by Openclipart, retrieved from: https://commons.wikimedia.org/wiki/File:Snellen_chart_by_Openclipart.svg on 6/7/2020. Creative Commons License Associated: CC0 1.0

## Slide 8
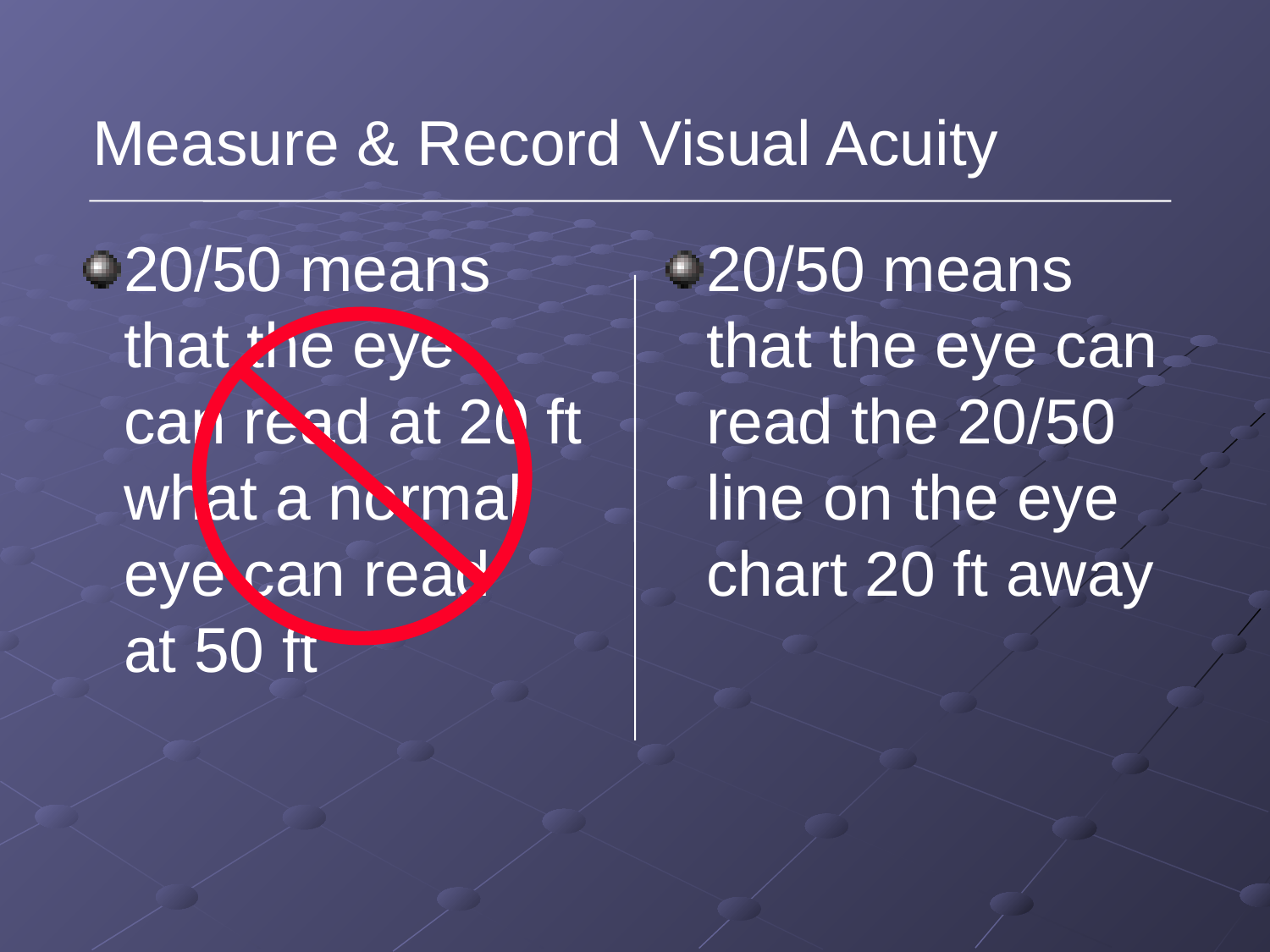

# Measure & Record Visual Acuity
20/50 means that the eye
can read at 20 ft what a normal eye can read
at 50 ft
20/50 means that the eye can read the 20/50 line on the eye chart 20 ft away

## Slide 9
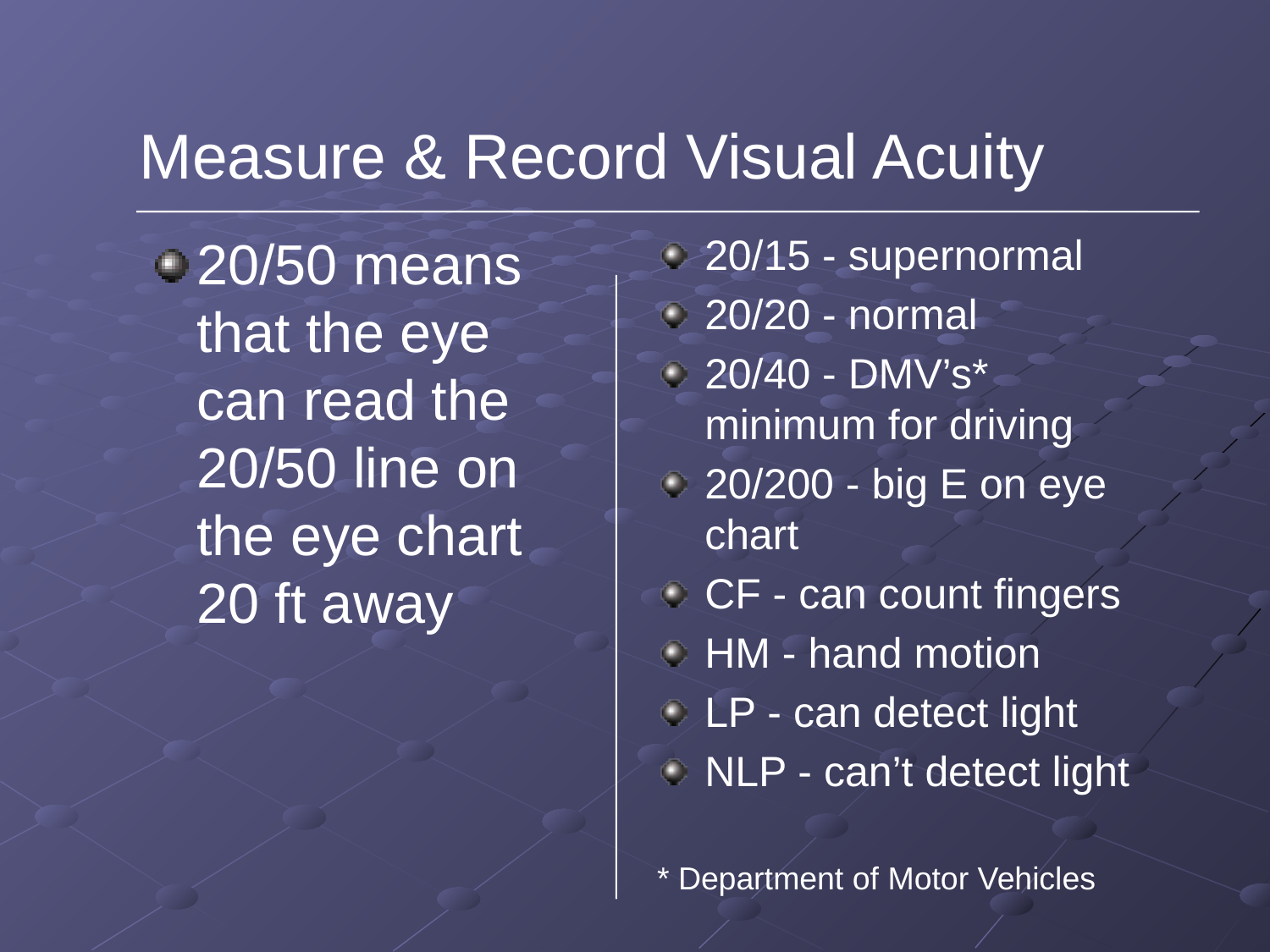

# Measure & Record Visual Acuity
20/50 means that the eye can read the 20/50 line on the eye chart 20 ft away
20/15 - supernormal
20/20 - normal
20/40 - DMV’s* minimum for driving
20/200 - big E on eye chart
CF - can count fingers
HM - hand motion
LP - can detect light
NLP - can’t detect light
* Department of Motor Vehicles

## Slide 10
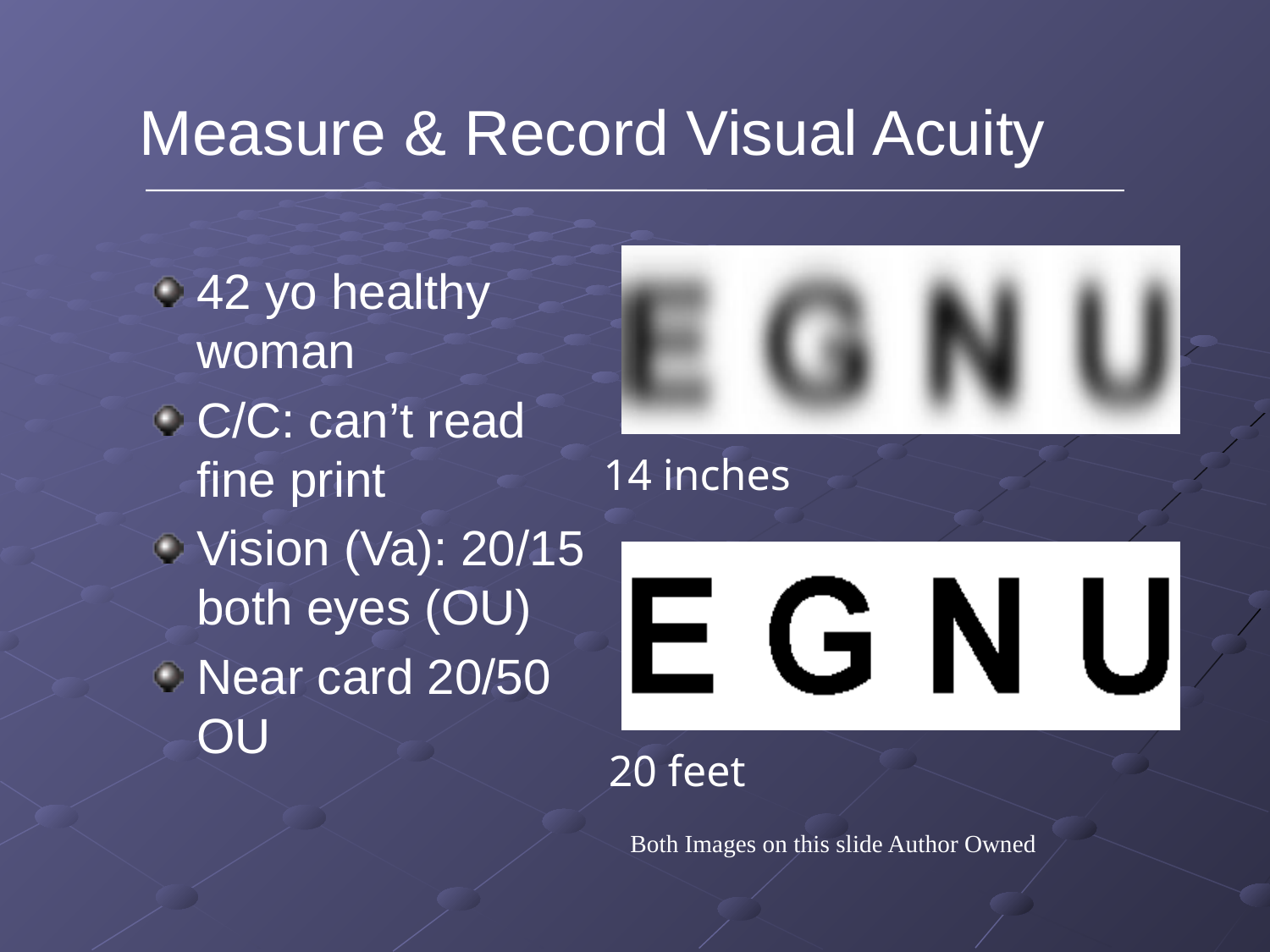

# Measure & Record Visual Acuity
42 yo healthy woman
C/C: can’t read fine print
Vision (Va): 20/15 both eyes (OU)
Near card 20/50 OU
14 inches
20 feet
Both Images on this slide Author Owned

## Slide 11
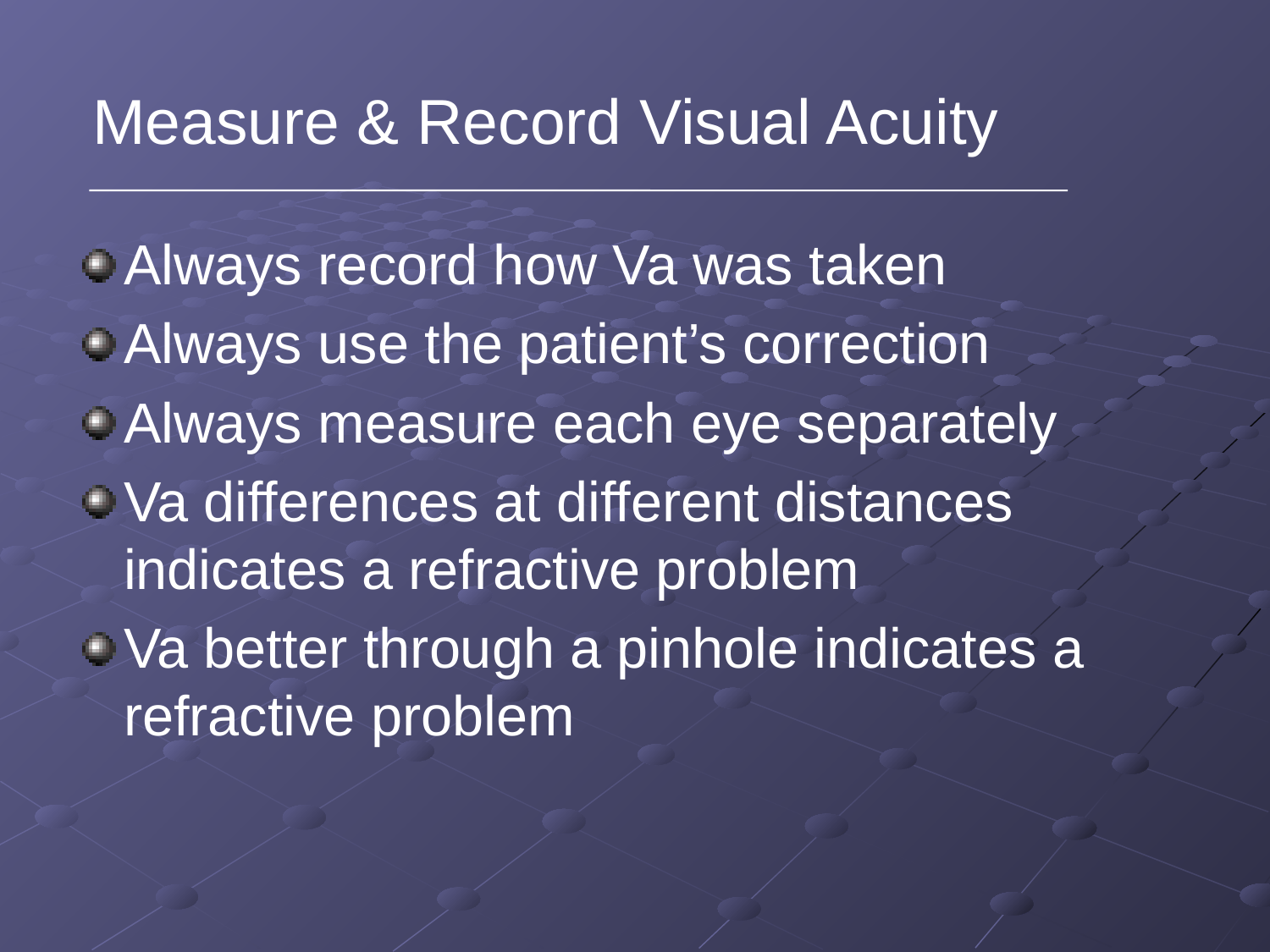

# Measure & Record Visual Acuity
Always record how Va was taken
Always use the patient’s correction
Always measure each eye separately
Va differences at different distances indicates a refractive problem
Va better through a pinhole indicates a refractive problem

## Slide 12
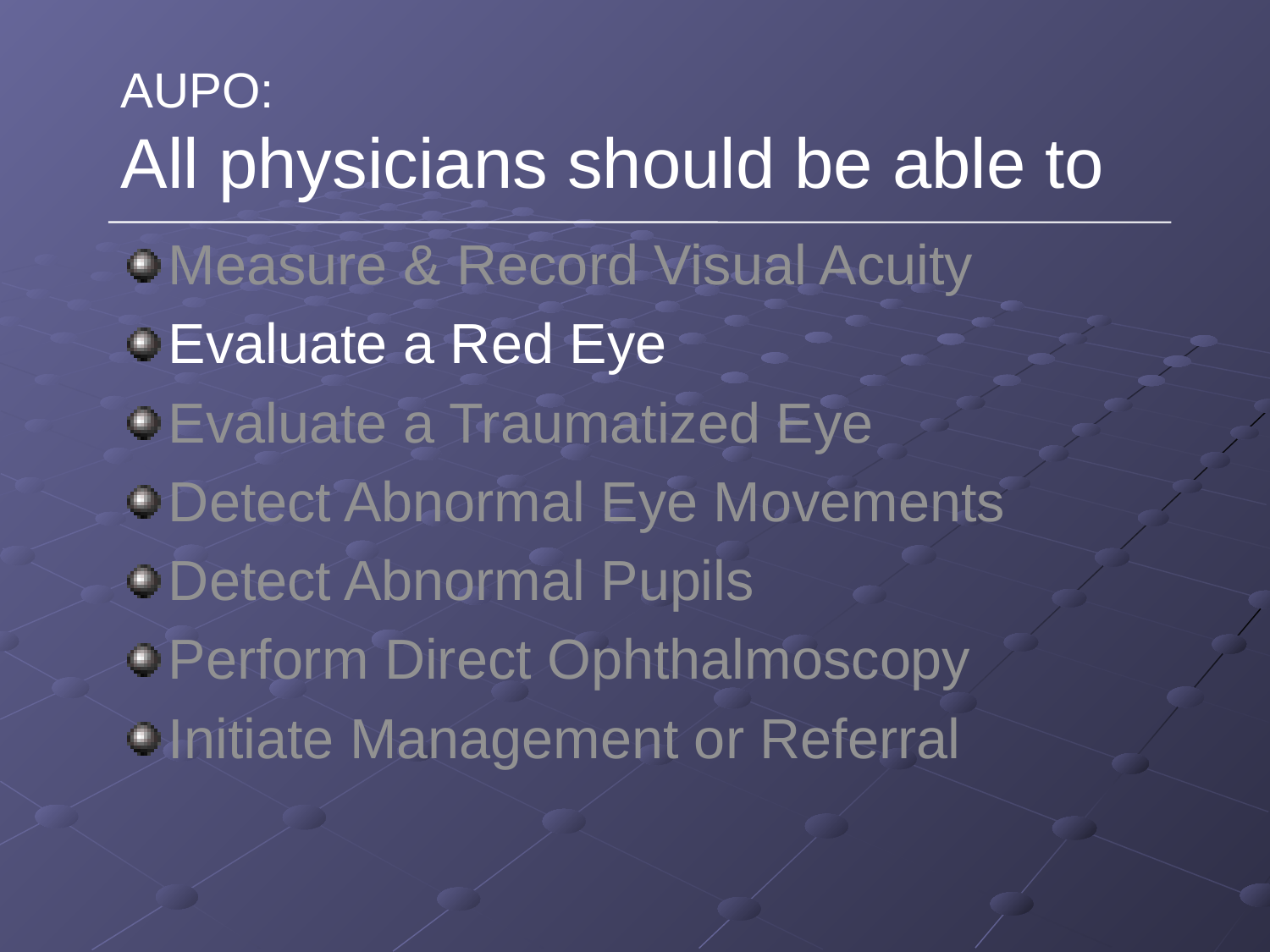

# AUPO: All physicians should be able to
Measure & Record Visual Acuity
Evaluate a Red Eye
Evaluate a Traumatized Eye
Detect Abnormal Eye Movements
Detect Abnormal Pupils
Perform Direct Ophthalmoscopy
Initiate Management or Referral

## Slide 13
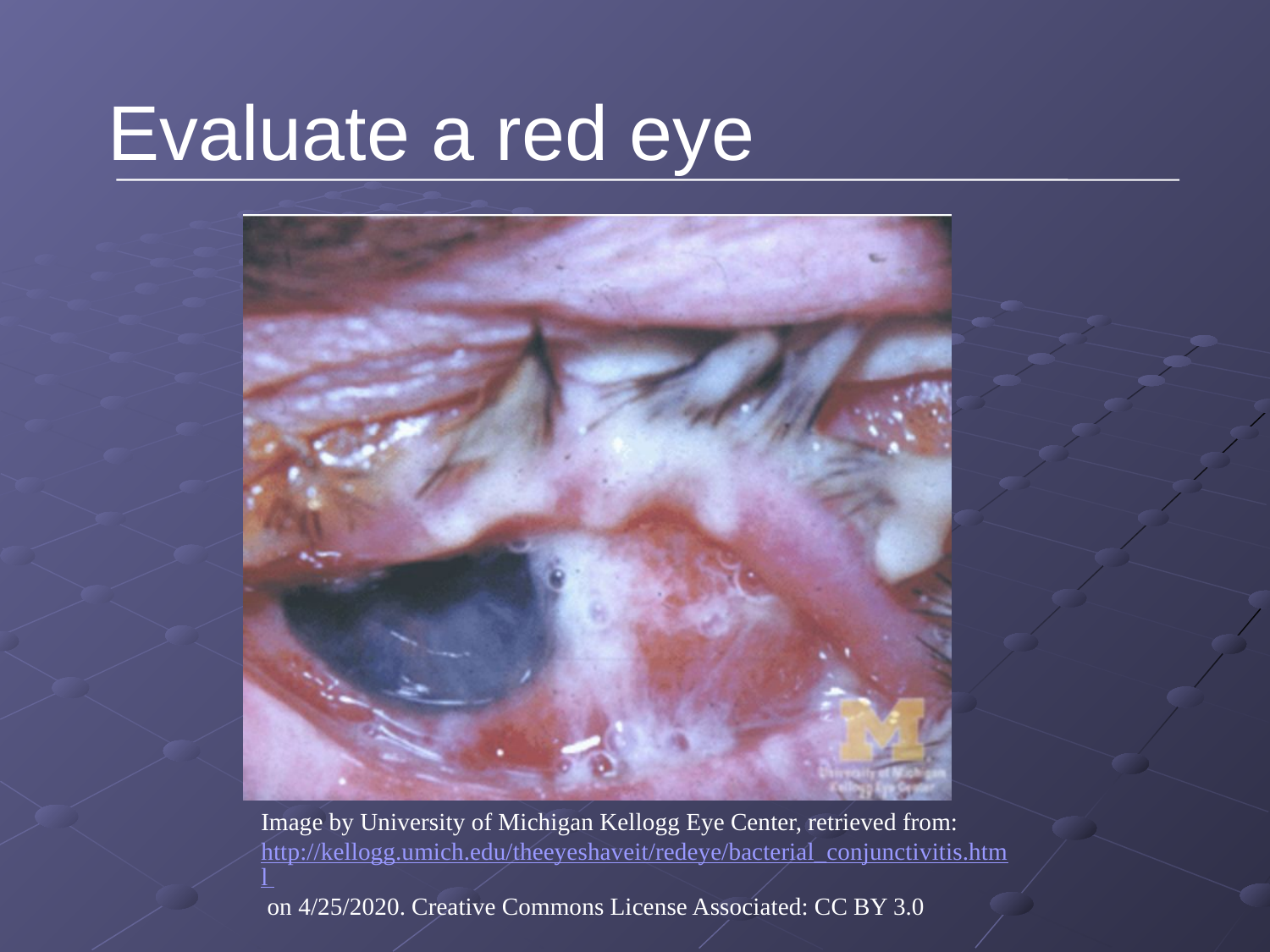

# Evaluate a red eye
Image by University of Michigan Kellogg Eye Center, retrieved from: http://kellogg.umich.edu/theeyeshaveit/redeye/bacterial_conjunctivitis.html on 4/25/2020. Creative Commons License Associated: CC BY 3.0

## Slide 14
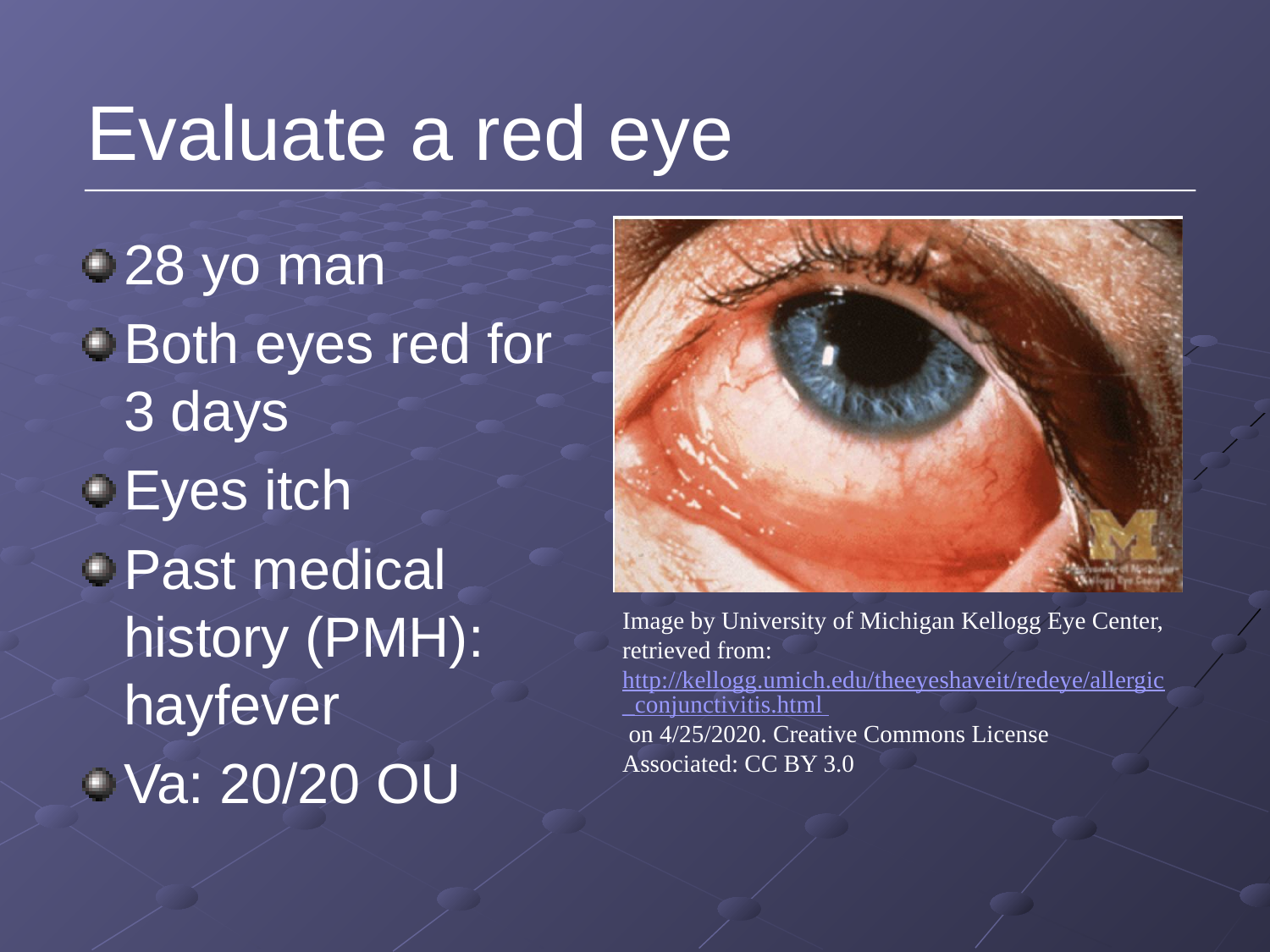

# Evaluate a red eye
28 yo man
Both eyes red for
3 days
Eyes itch
Past medical history (PMH): hayfever
Va: 20/20 OU
Image by University of Michigan Kellogg Eye Center, retrieved from: http://kellogg.umich.edu/theeyeshaveit/redeye/allergic_conjunctivitis.html on 4/25/2020. Creative Commons License Associated: CC BY 3.0

## Slide 15
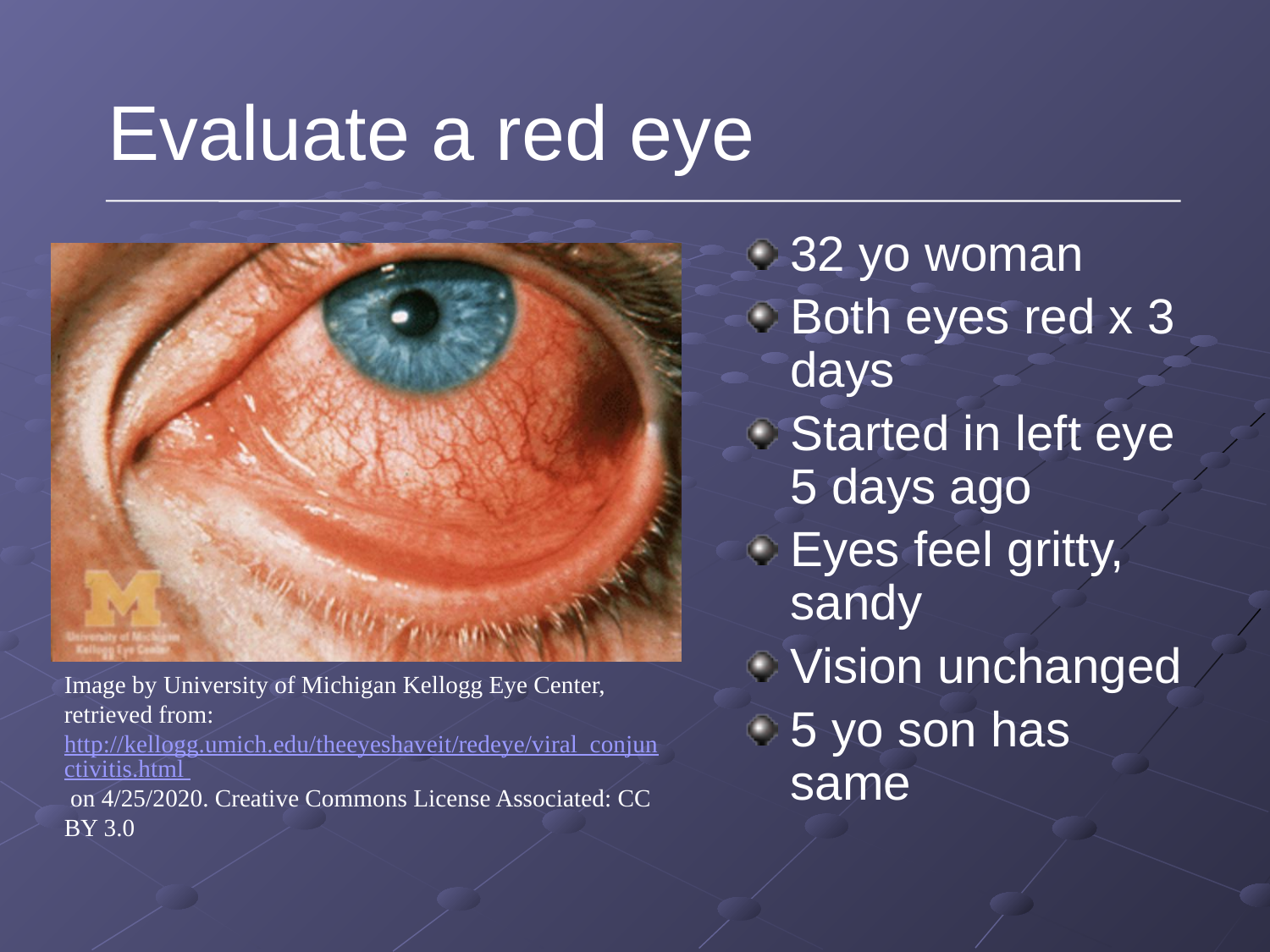

# Evaluate a red eye
32 yo woman
Both eyes red x 3 days
Started in left eye 5 days ago
Eyes feel gritty, sandy
Vision unchanged
5 yo son has same
Image by University of Michigan Kellogg Eye Center, retrieved from: http://kellogg.umich.edu/theeyeshaveit/redeye/viral_conjunctivitis.html on 4/25/2020. Creative Commons License Associated: CC BY 3.0

## Slide 16
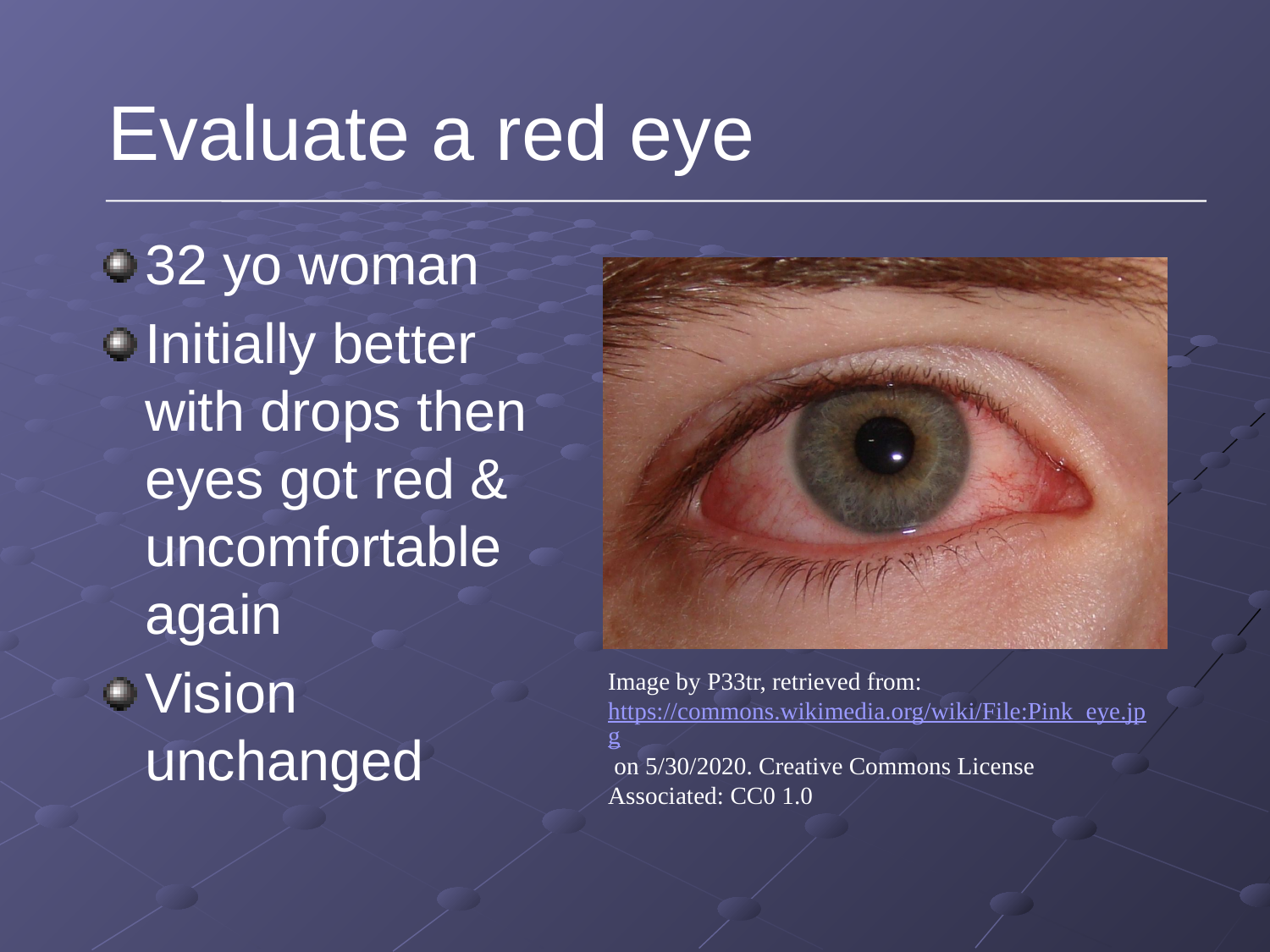

# Evaluate a red eye
32 yo woman
Initially better with drops then eyes got red & uncomfortable again
Vision unchanged
Image by P33tr, retrieved from: https://commons.wikimedia.org/wiki/File:Pink_eye.jpg on 5/30/2020. Creative Commons License Associated: CC0 1.0

## Slide 17
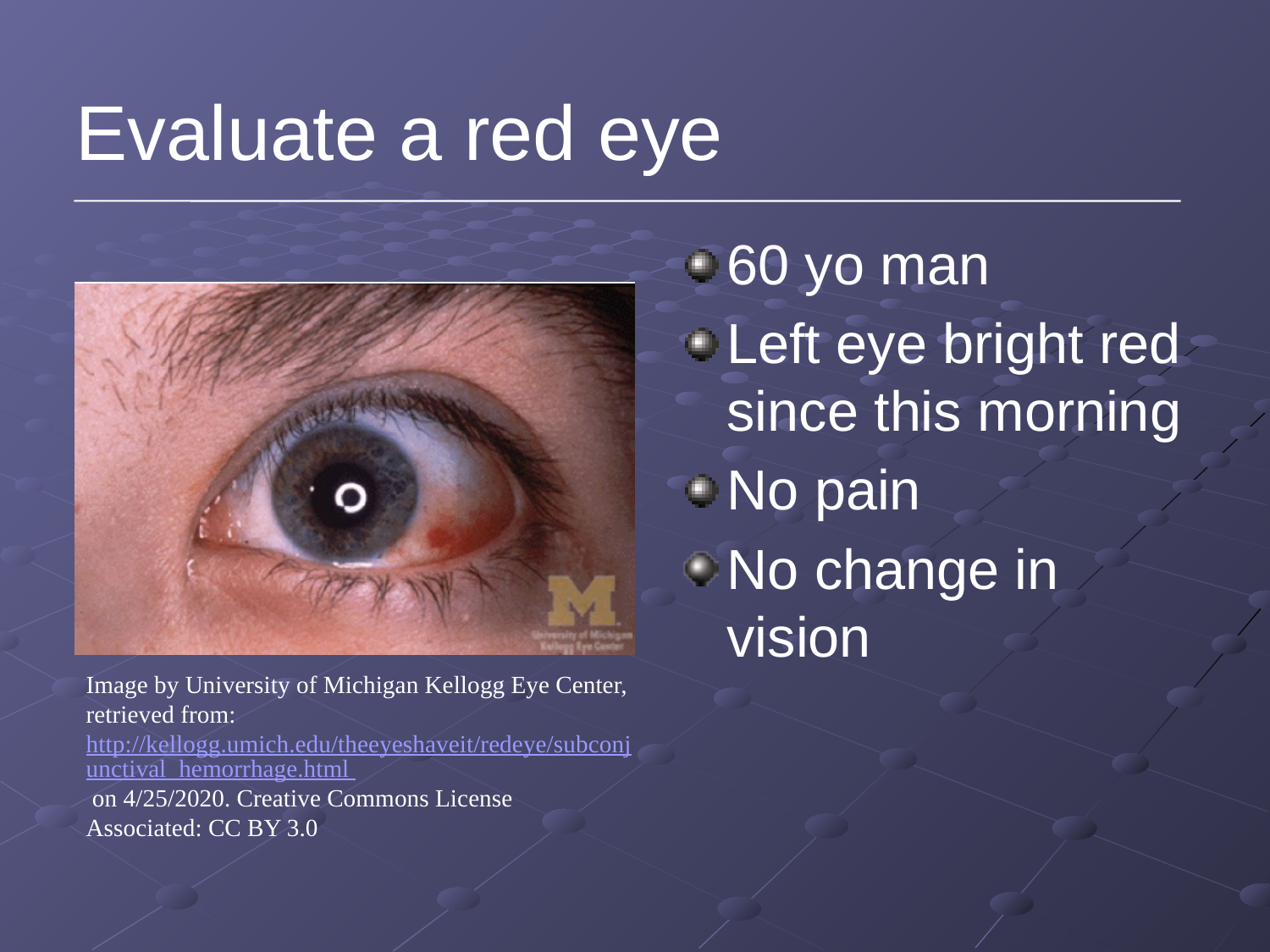

# Evaluate a red eye
60 yo man
Left eye bright red since this morning
No pain
No change in vision
Image by University of Michigan Kellogg Eye Center, retrieved from: http://kellogg.umich.edu/theeyeshaveit/redeye/subconjunctival_hemorrhage.html on 4/25/2020. Creative Commons License Associated: CC BY 3.0

## Slide 18
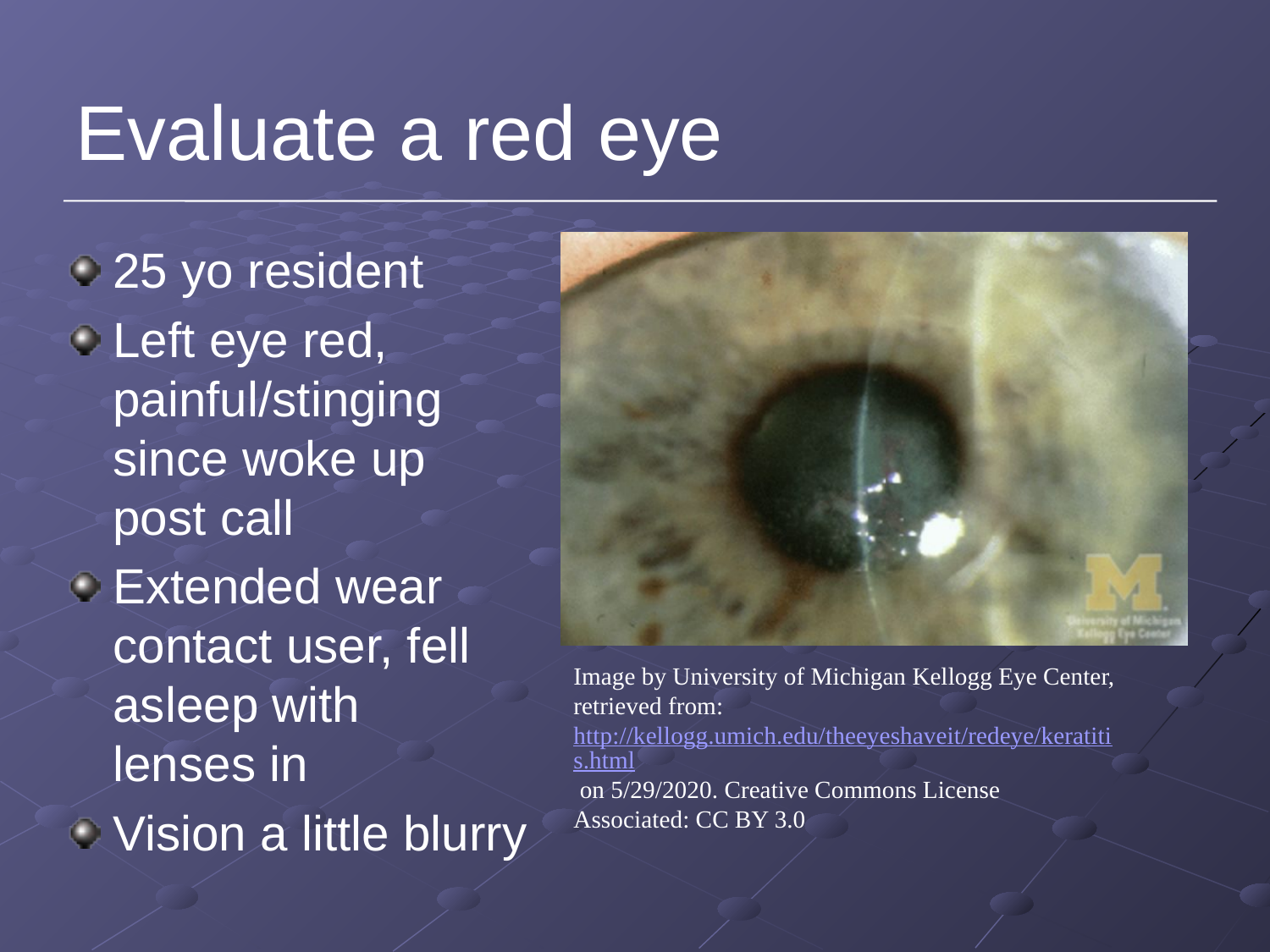

# Evaluate a red eye
25 yo resident
Left eye red, painful/stinging since woke up post call
Extended wear contact user, fell asleep with
lenses in
Vision a little blurry
Image by University of Michigan Kellogg Eye Center, retrieved from: http://kellogg.umich.edu/theeyeshaveit/redeye/keratitis.html on 5/29/2020. Creative Commons License Associated: CC BY 3.0

## Slide 19
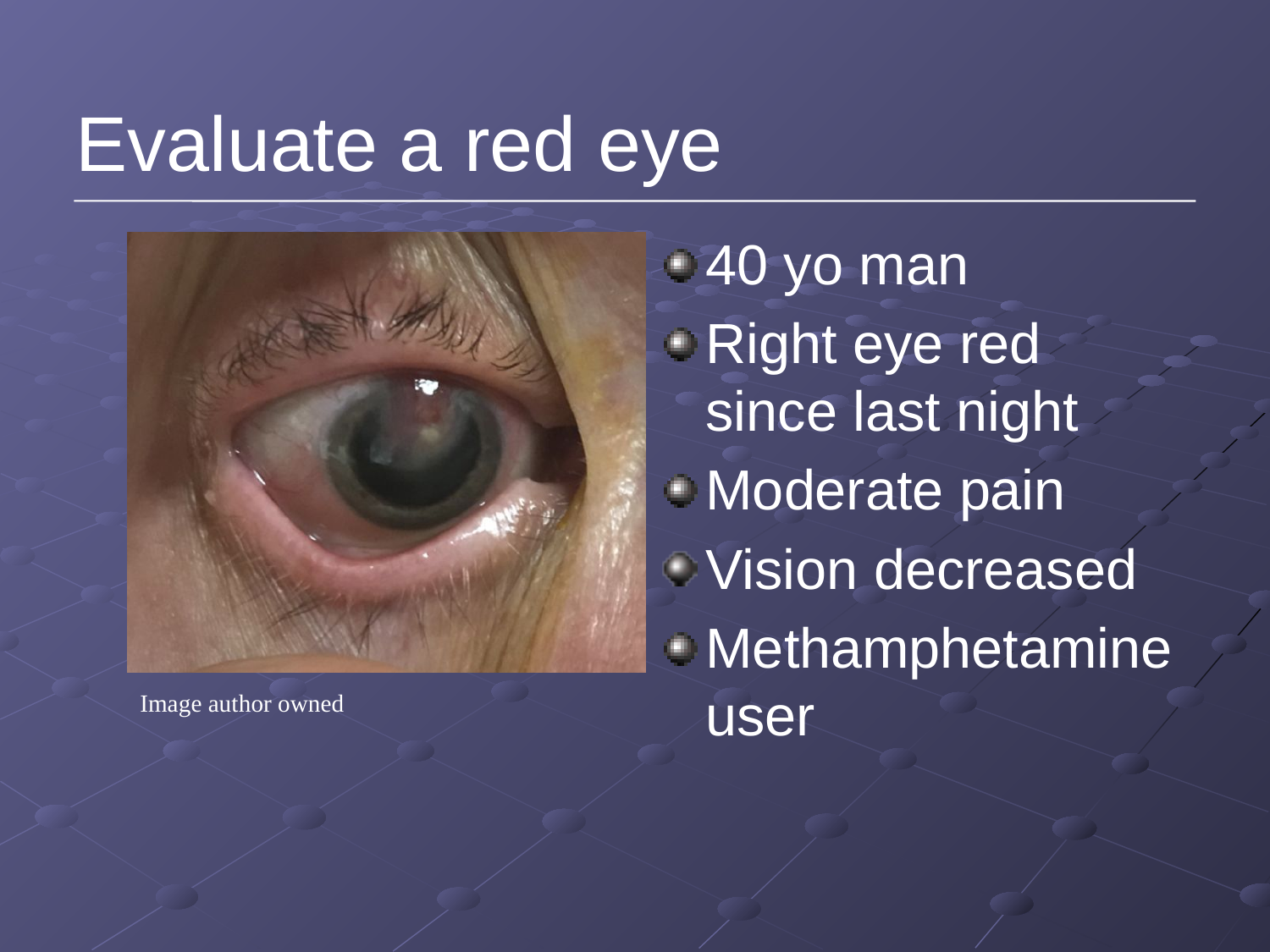

# Evaluate a red eye
40 yo man
Right eye red since last night
Moderate pain
Vision decreased
Methamphetamine user
Image author owned

## Slide 20
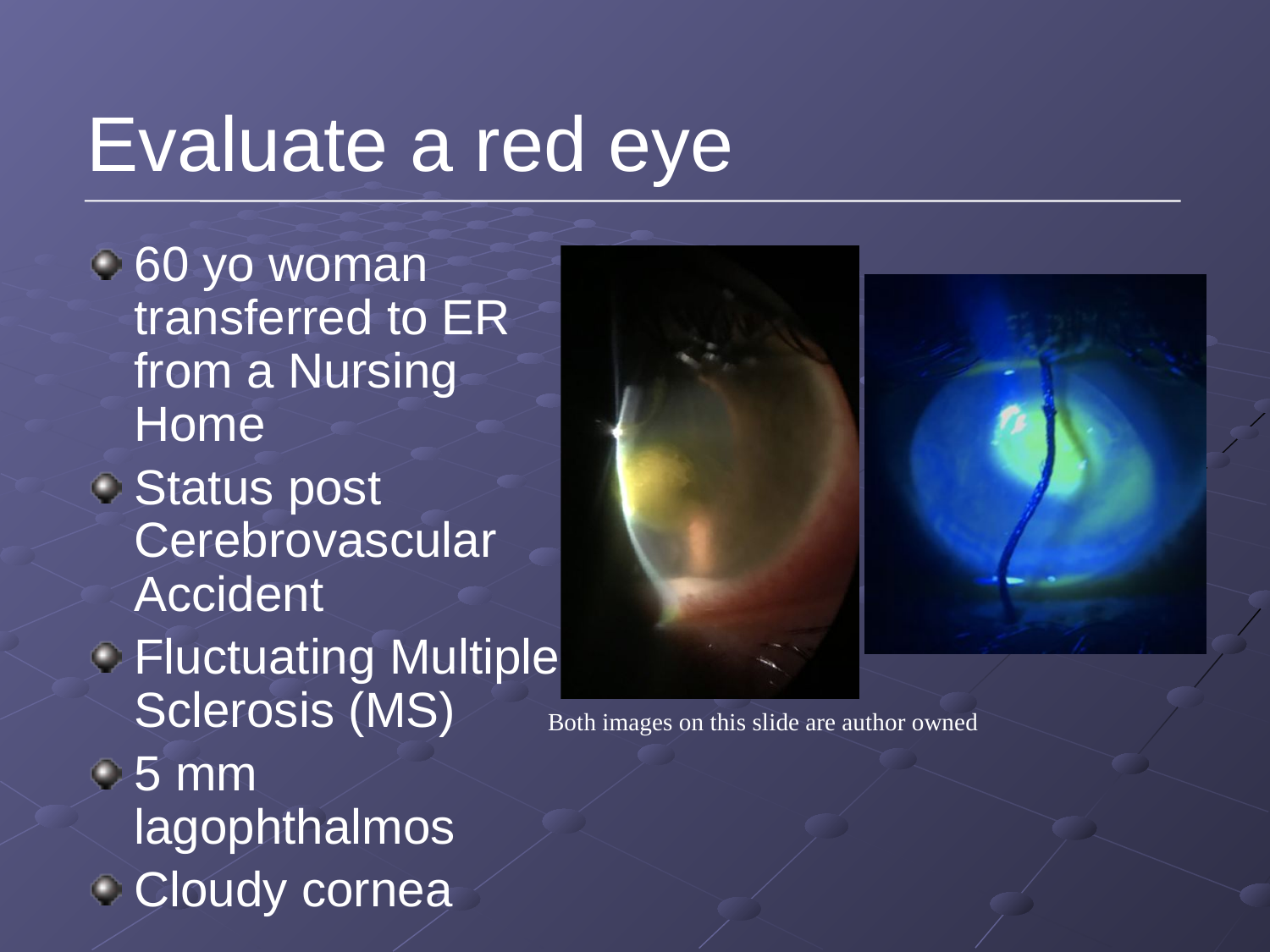

# Evaluate a red eye
60 yo woman transferred to ER from a Nursing Home
Status post Cerebrovascular Accident
Fluctuating Multiple Sclerosis (MS)
5 mm lagophthalmos
Cloudy cornea
Both images on this slide are author owned

## Slide 21
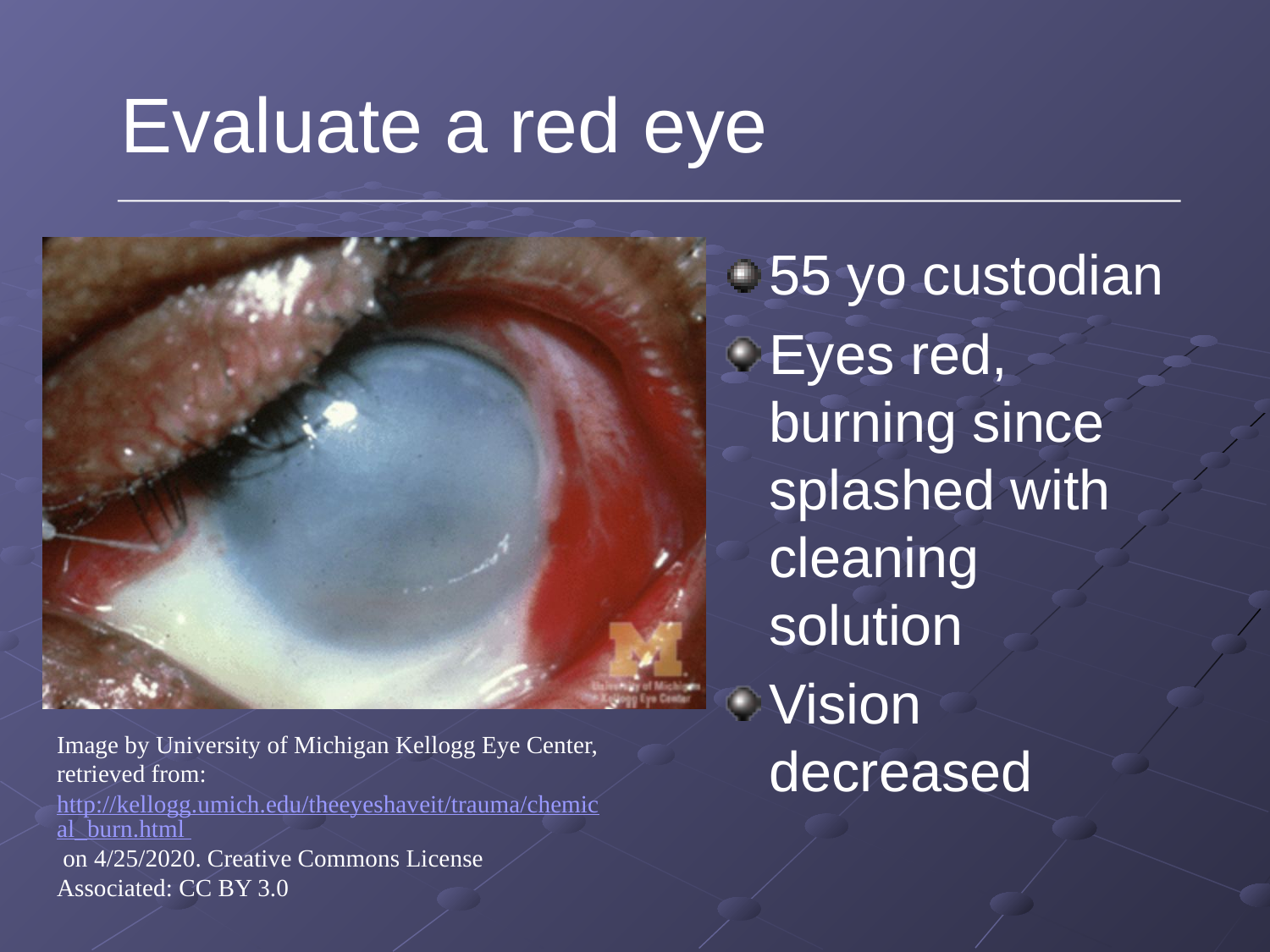

# Evaluate a red eye
55 yo custodian
Eyes red, burning since splashed with cleaning solution
Vision decreased
Image by University of Michigan Kellogg Eye Center, retrieved from: http://kellogg.umich.edu/theeyeshaveit/trauma/chemical_burn.html on 4/25/2020. Creative Commons License Associated: CC BY 3.0

## Slide 22
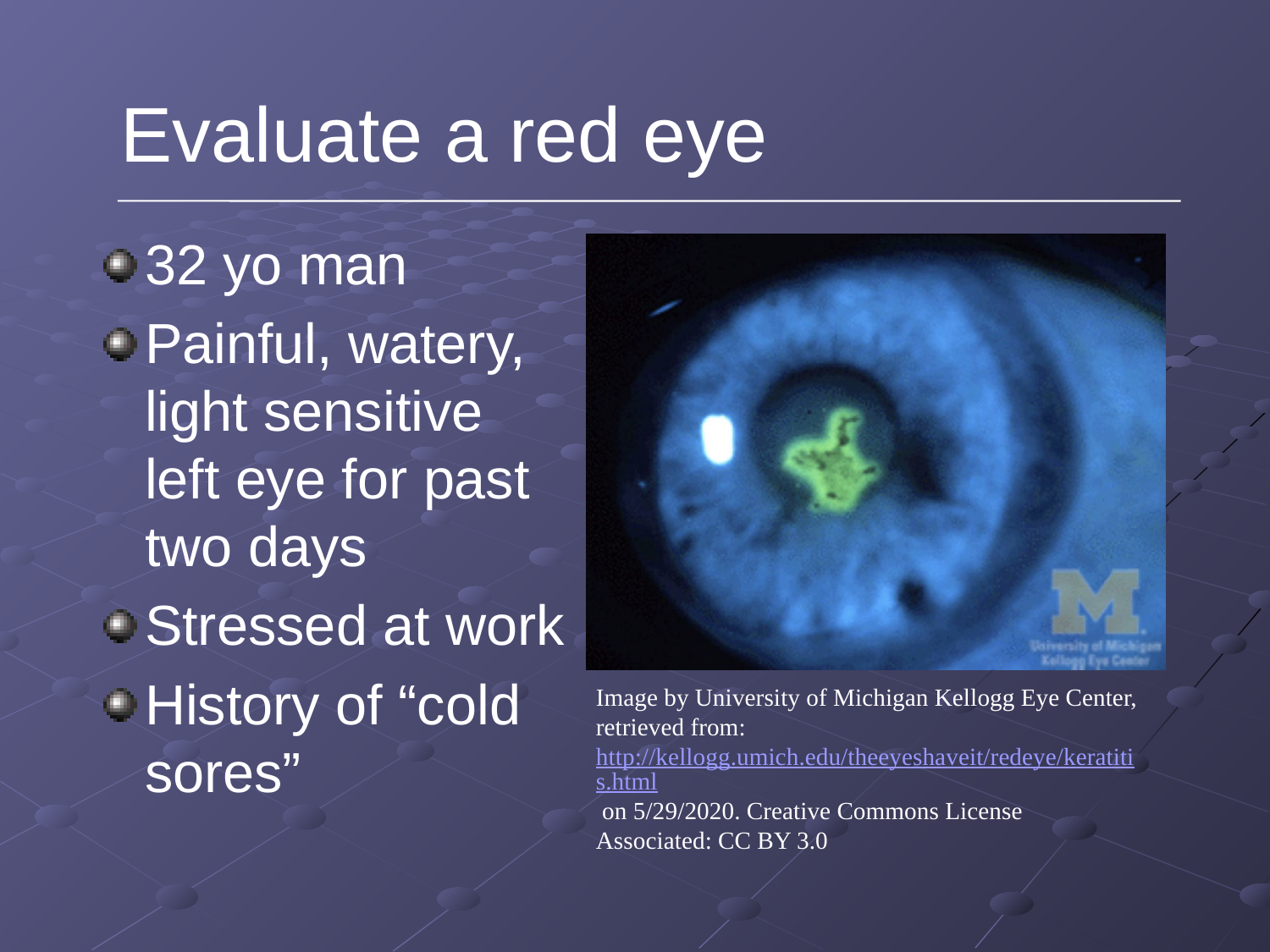

# Evaluate a red eye
32 yo man
Painful, watery, light sensitive left eye for past two days
Stressed at work
History of “cold sores”
Image by University of Michigan Kellogg Eye Center, retrieved from: http://kellogg.umich.edu/theeyeshaveit/redeye/keratitis.html on 5/29/2020. Creative Commons License Associated: CC BY 3.0

## Slide 23
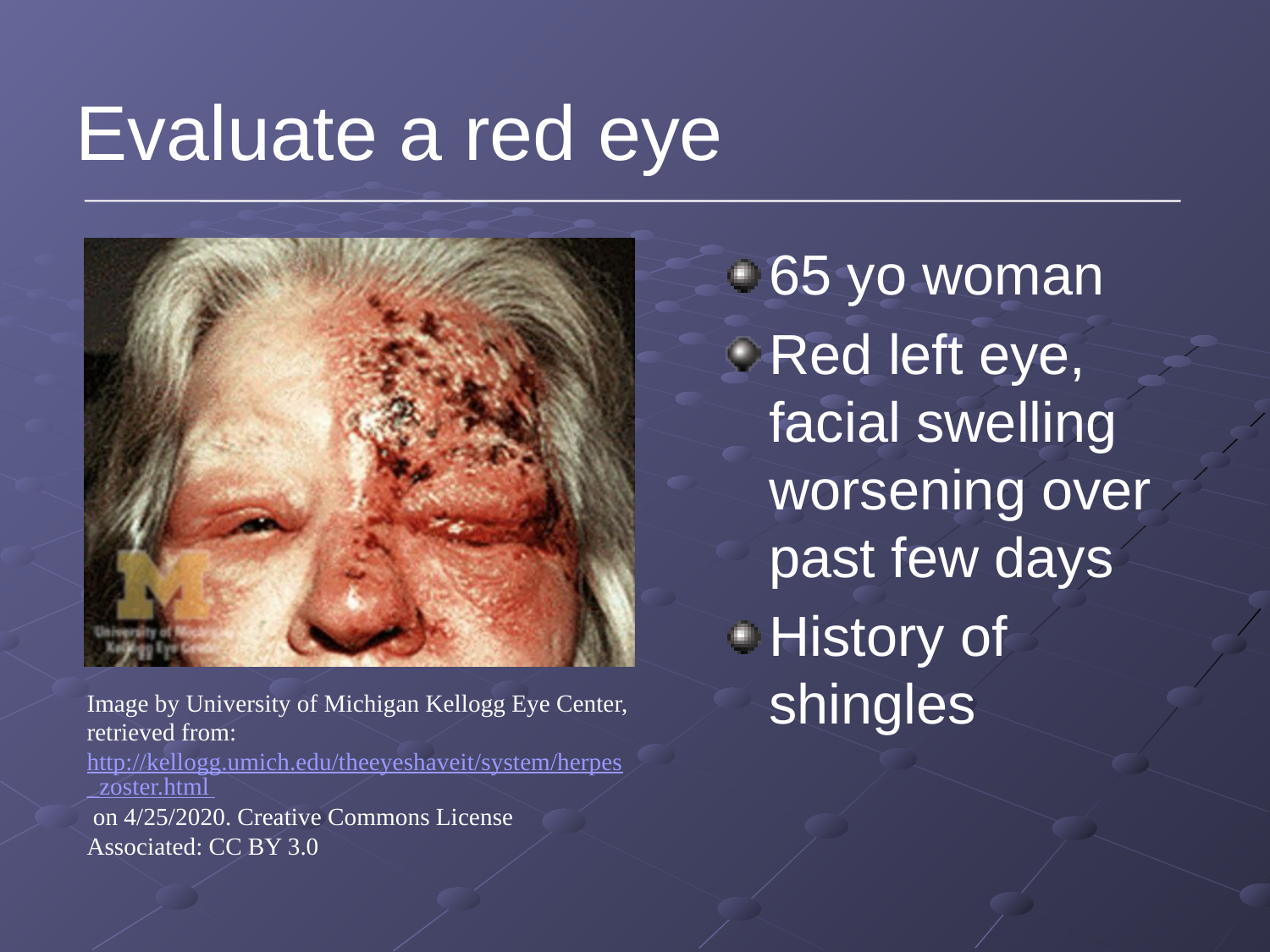

# Evaluate a red eye
65 yo woman
Red left eye, facial swelling worsening over past few days
History of shingles
Image by University of Michigan Kellogg Eye Center, retrieved from: http://kellogg.umich.edu/theeyeshaveit/system/herpes_zoster.html on 4/25/2020. Creative Commons License Associated: CC BY 3.0

## Slide 24
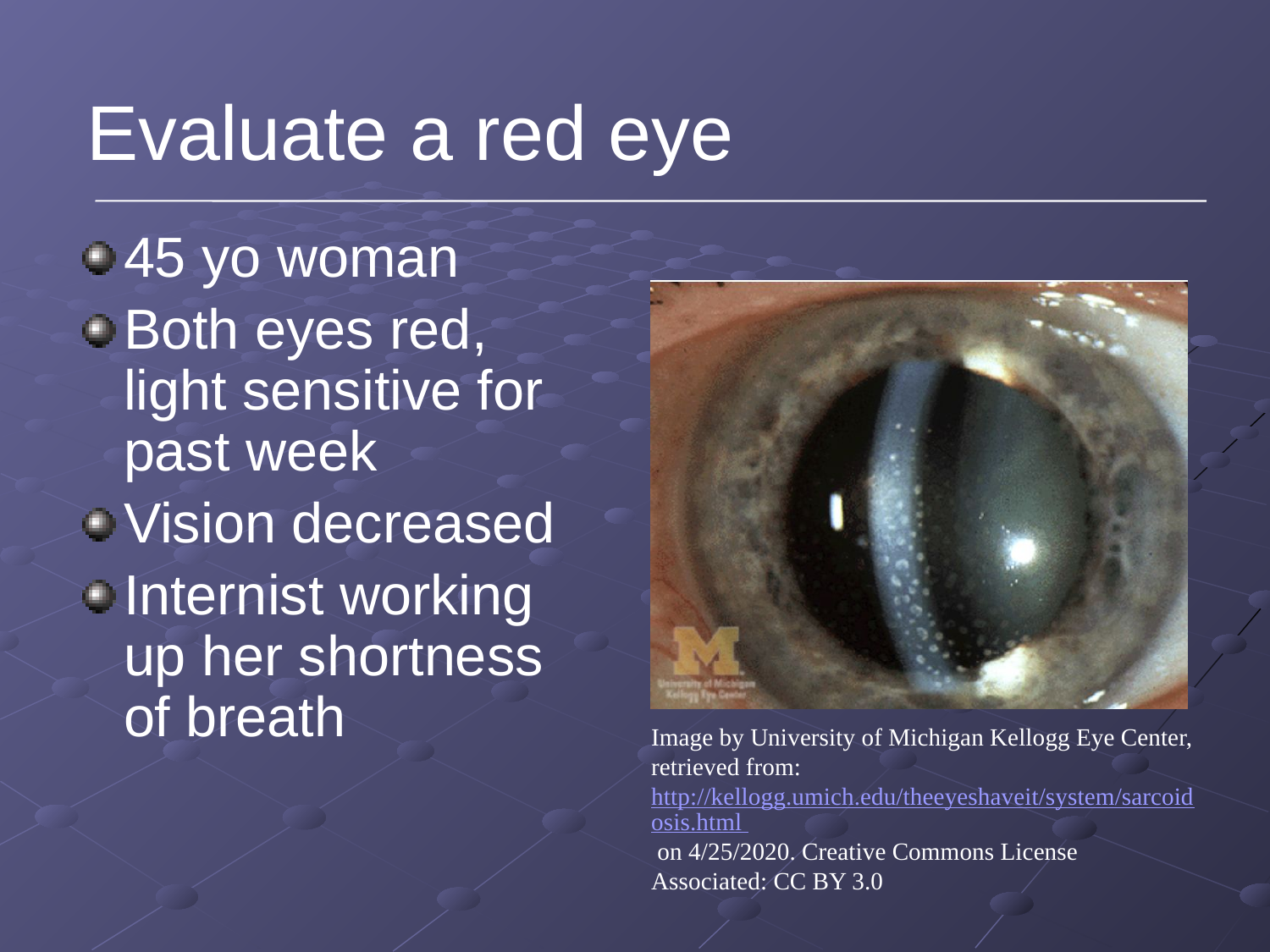

# Evaluate a red eye
45 yo woman
Both eyes red, light sensitive for past week
Vision decreased
Internist working up her shortness of breath
Image by University of Michigan Kellogg Eye Center, retrieved from: http://kellogg.umich.edu/theeyeshaveit/system/sarcoidosis.html on 4/25/2020. Creative Commons License Associated: CC BY 3.0

## Slide 25
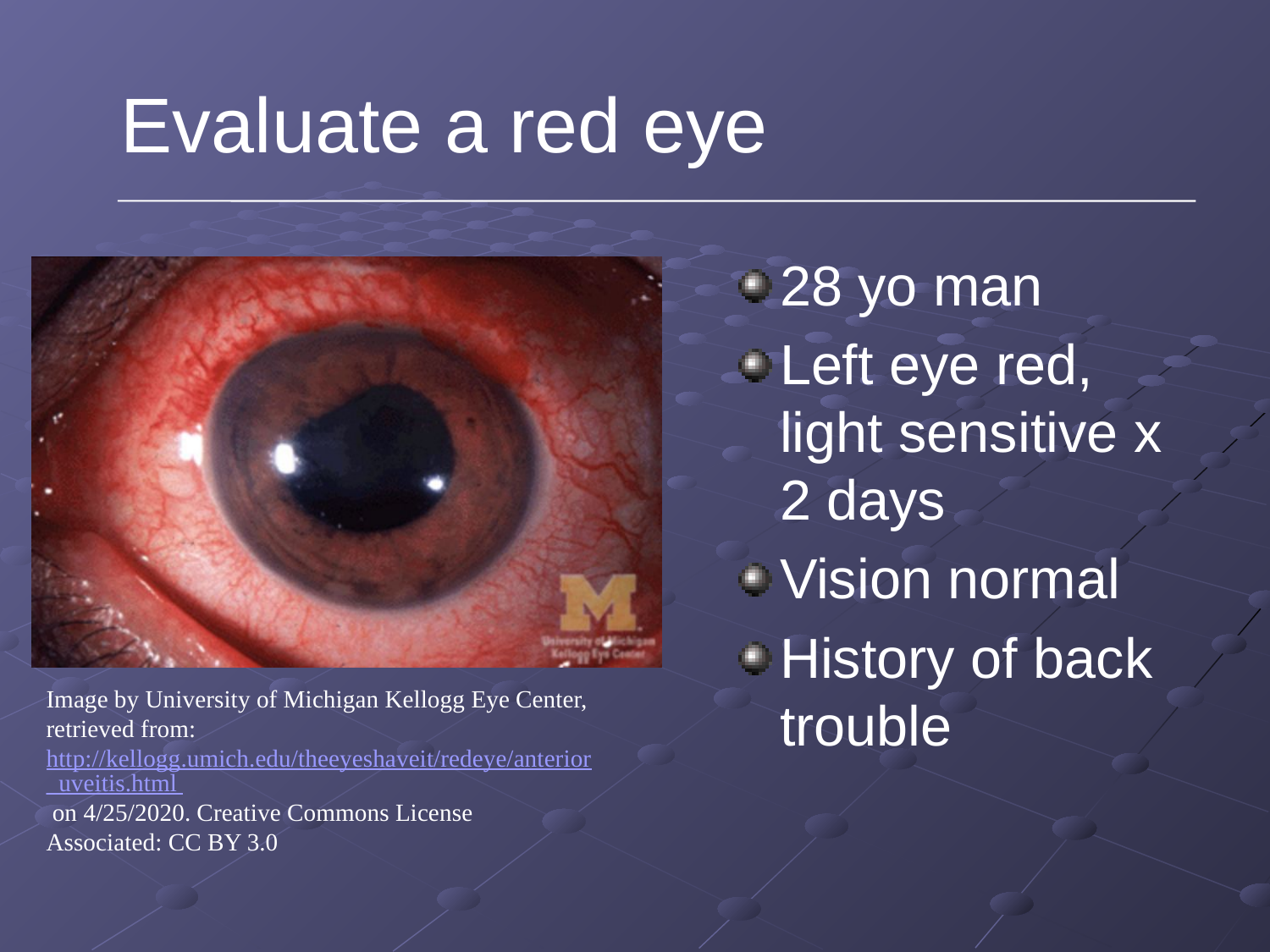

# Evaluate a red eye
28 yo man
Left eye red, light sensitive x 2 days
Vision normal
History of back trouble
Image by University of Michigan Kellogg Eye Center, retrieved from: http://kellogg.umich.edu/theeyeshaveit/redeye/anterior_uveitis.html on 4/25/2020. Creative Commons License Associated: CC BY 3.0

## Slide 26
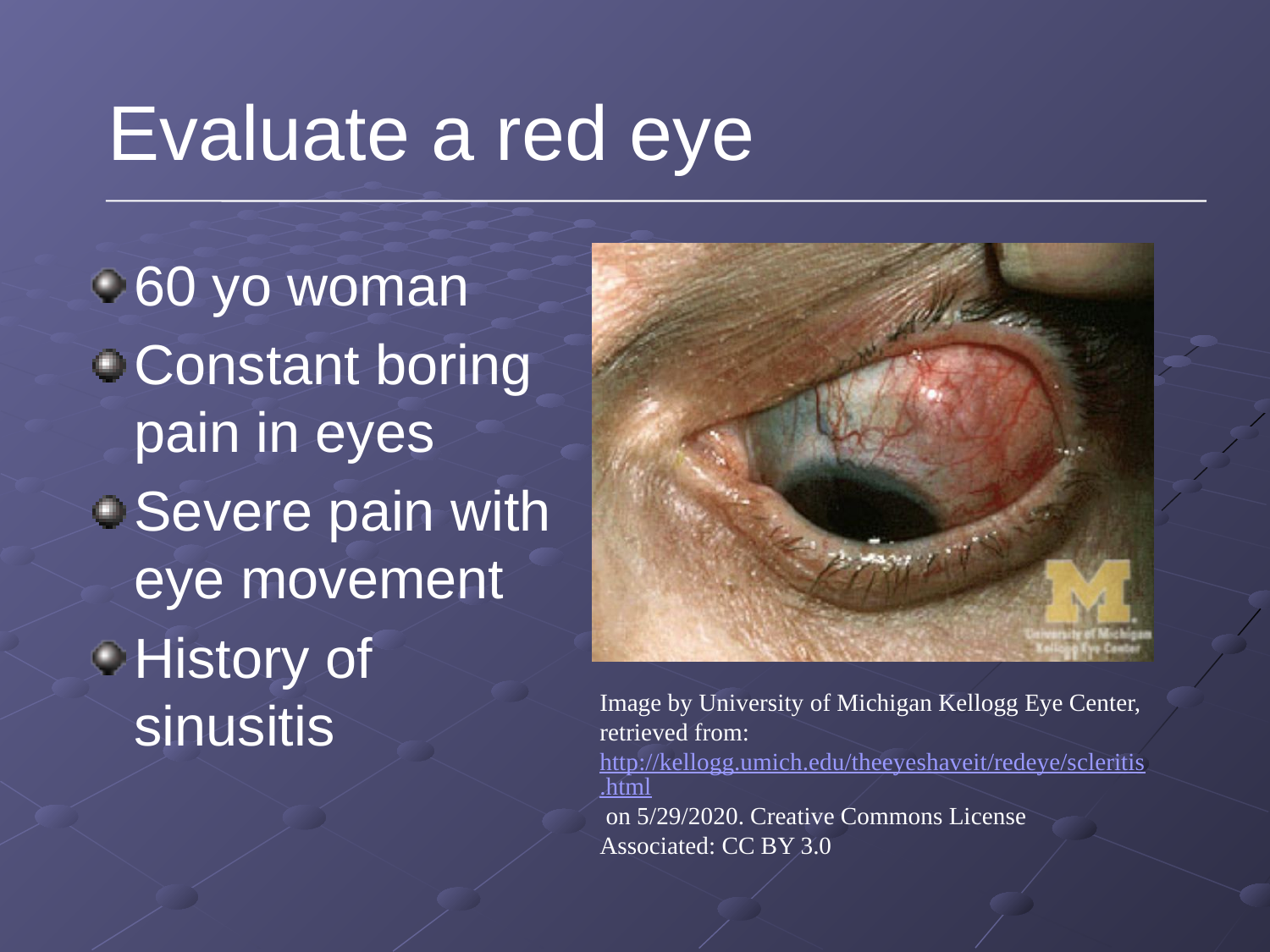

# Evaluate a red eye
60 yo woman
Constant boring pain in eyes
Severe pain with eye movement
History of sinusitis
Image by University of Michigan Kellogg Eye Center, retrieved from: http://kellogg.umich.edu/theeyeshaveit/redeye/scleritis.html on 5/29/2020. Creative Commons License Associated: CC BY 3.0

## Slide 27
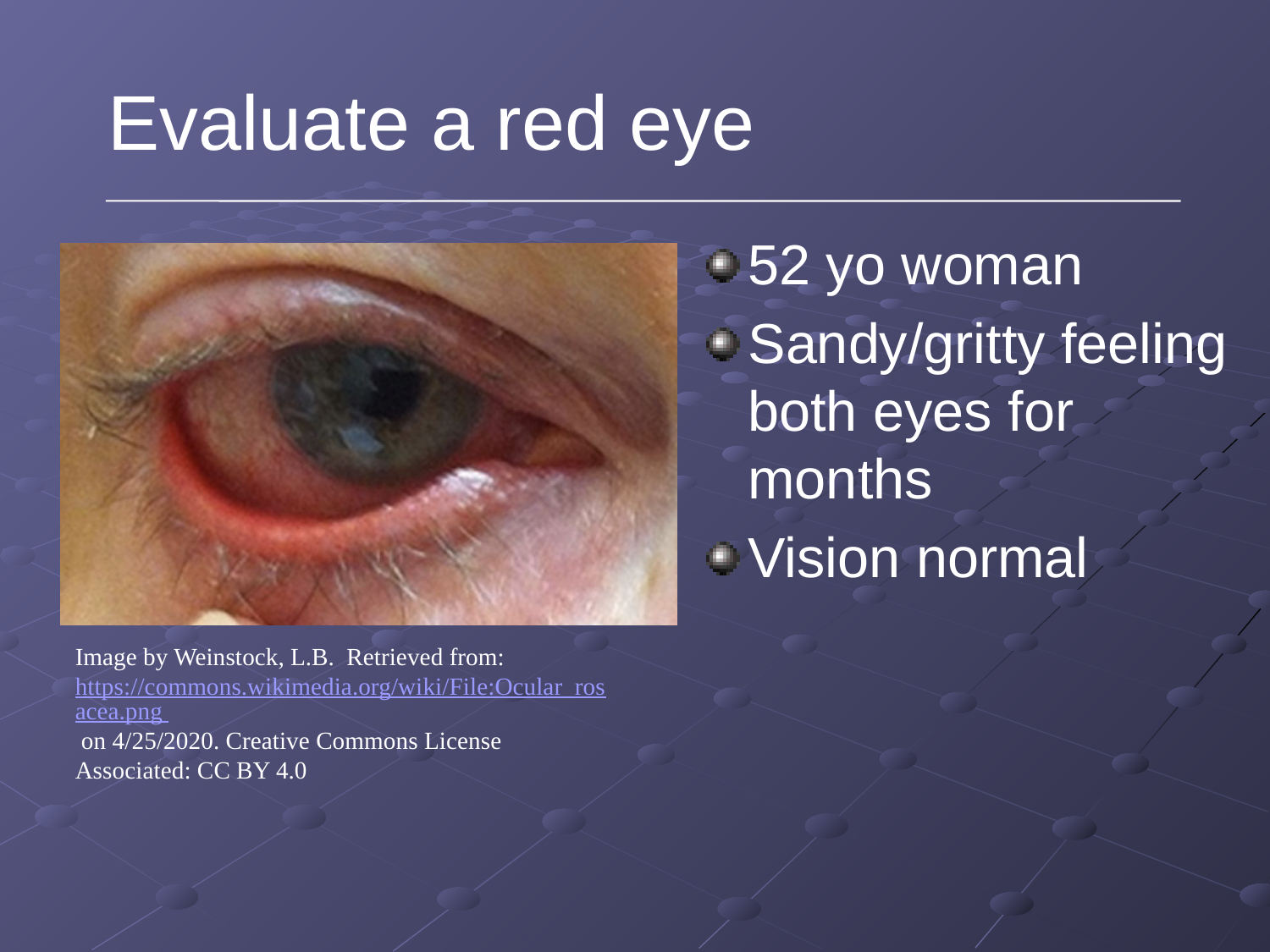

# Evaluate a red eye
52 yo woman
Sandy/gritty feeling both eyes for months
Vision normal
Image by Weinstock, L.B. Retrieved from: https://commons.wikimedia.org/wiki/File:Ocular_rosacea.png on 4/25/2020. Creative Commons License Associated: CC BY 4.0

## Slide 28
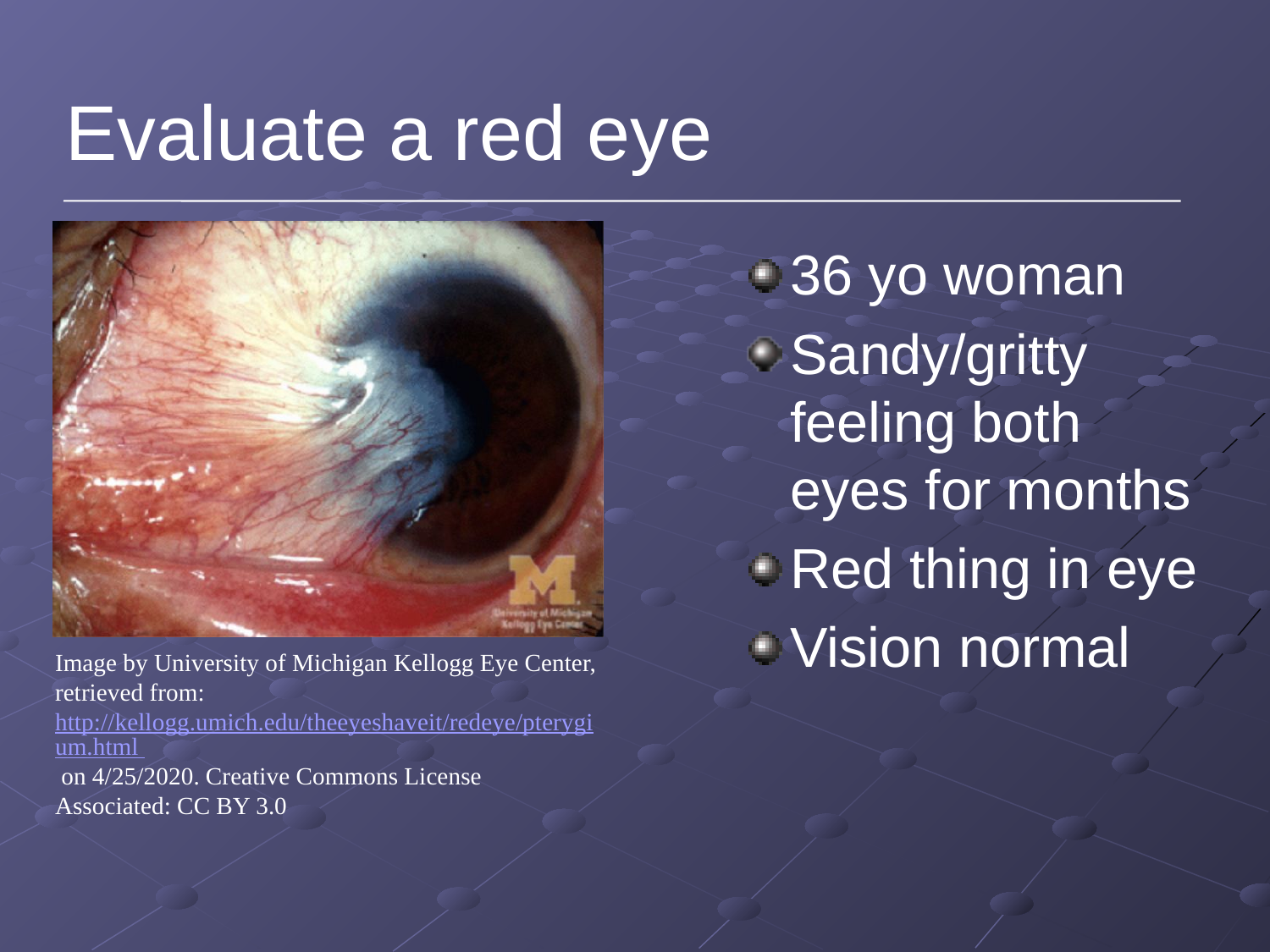

# Evaluate a red eye
36 yo woman
Sandy/gritty feeling both eyes for months
Red thing in eye
Vision normal
Image by University of Michigan Kellogg Eye Center, retrieved from: http://kellogg.umich.edu/theeyeshaveit/redeye/pterygium.html on 4/25/2020. Creative Commons License Associated: CC BY 3.0

## Slide 29
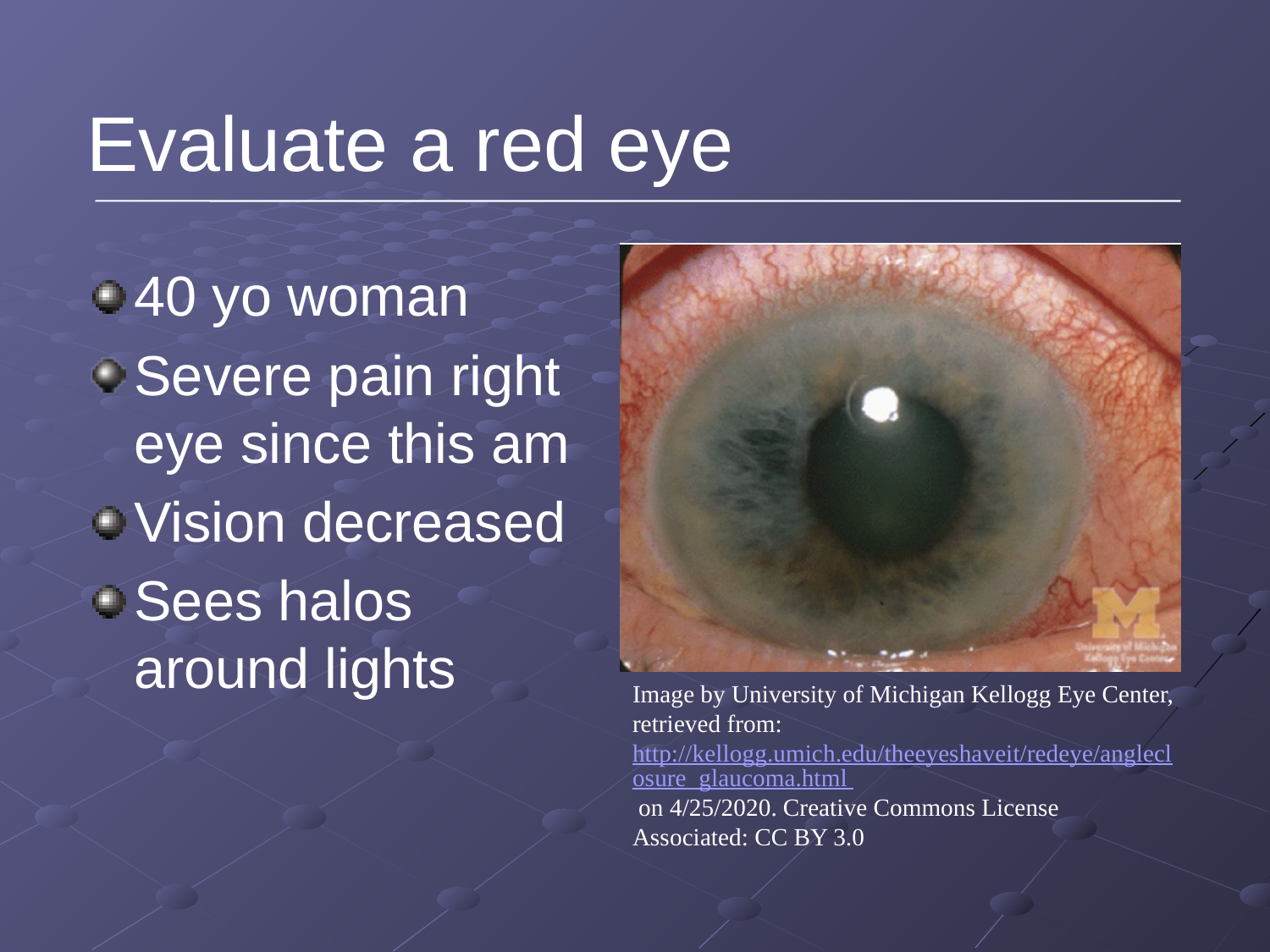

# Evaluate a red eye
40 yo woman
Severe pain right eye since this am
Vision decreased
Sees halos around lights
Image by University of Michigan Kellogg Eye Center, retrieved from: http://kellogg.umich.edu/theeyeshaveit/redeye/angleclosure_glaucoma.html on 4/25/2020. Creative Commons License Associated: CC BY 3.0

## Slide 30
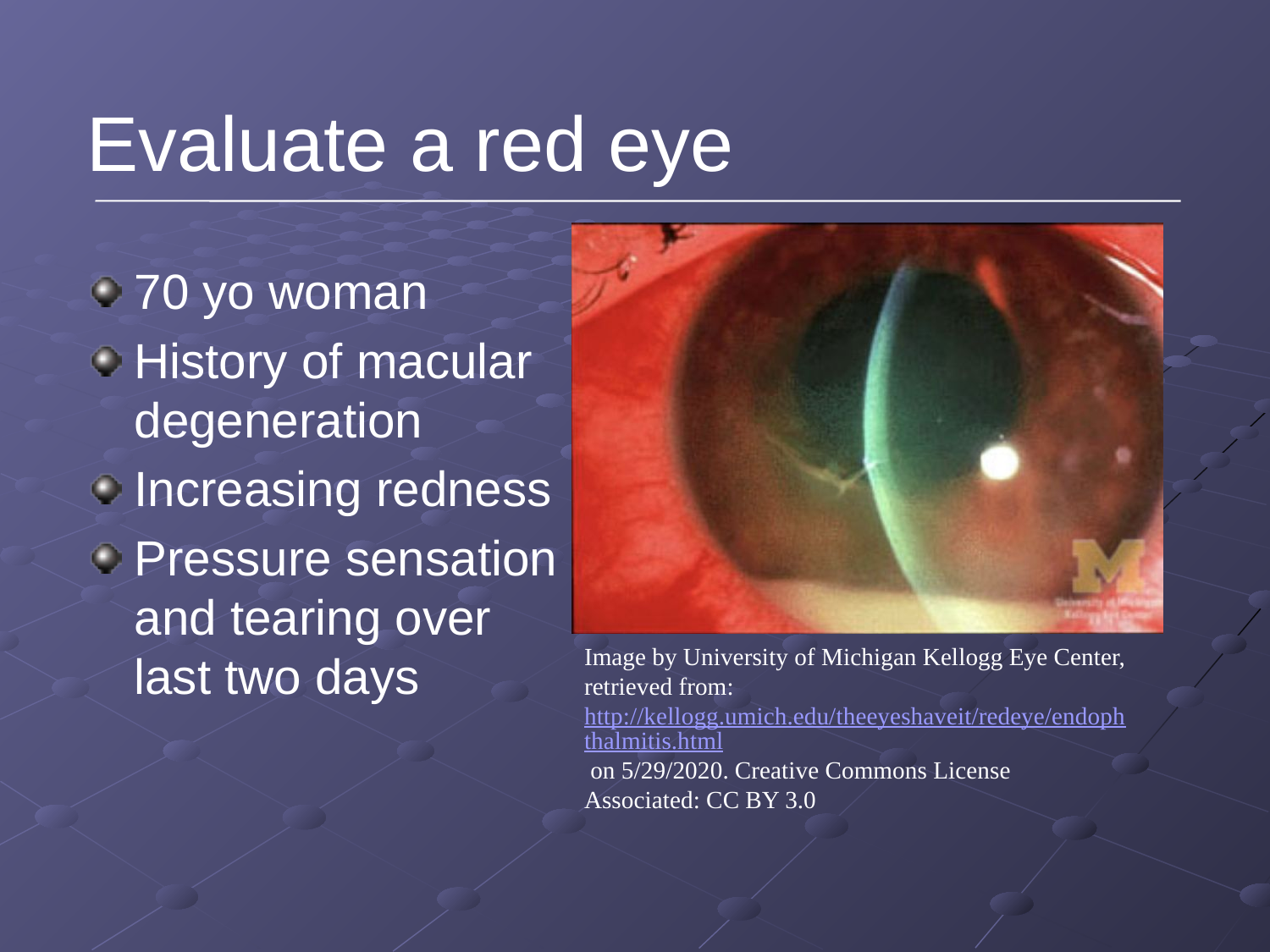

# Evaluate a red eye
70 yo woman
History of macular degeneration
Increasing redness
Pressure sensation and tearing over last two days
Image by University of Michigan Kellogg Eye Center, retrieved from: http://kellogg.umich.edu/theeyeshaveit/redeye/endophthalmitis.html on 5/29/2020. Creative Commons License Associated: CC BY 3.0

## Slide 31
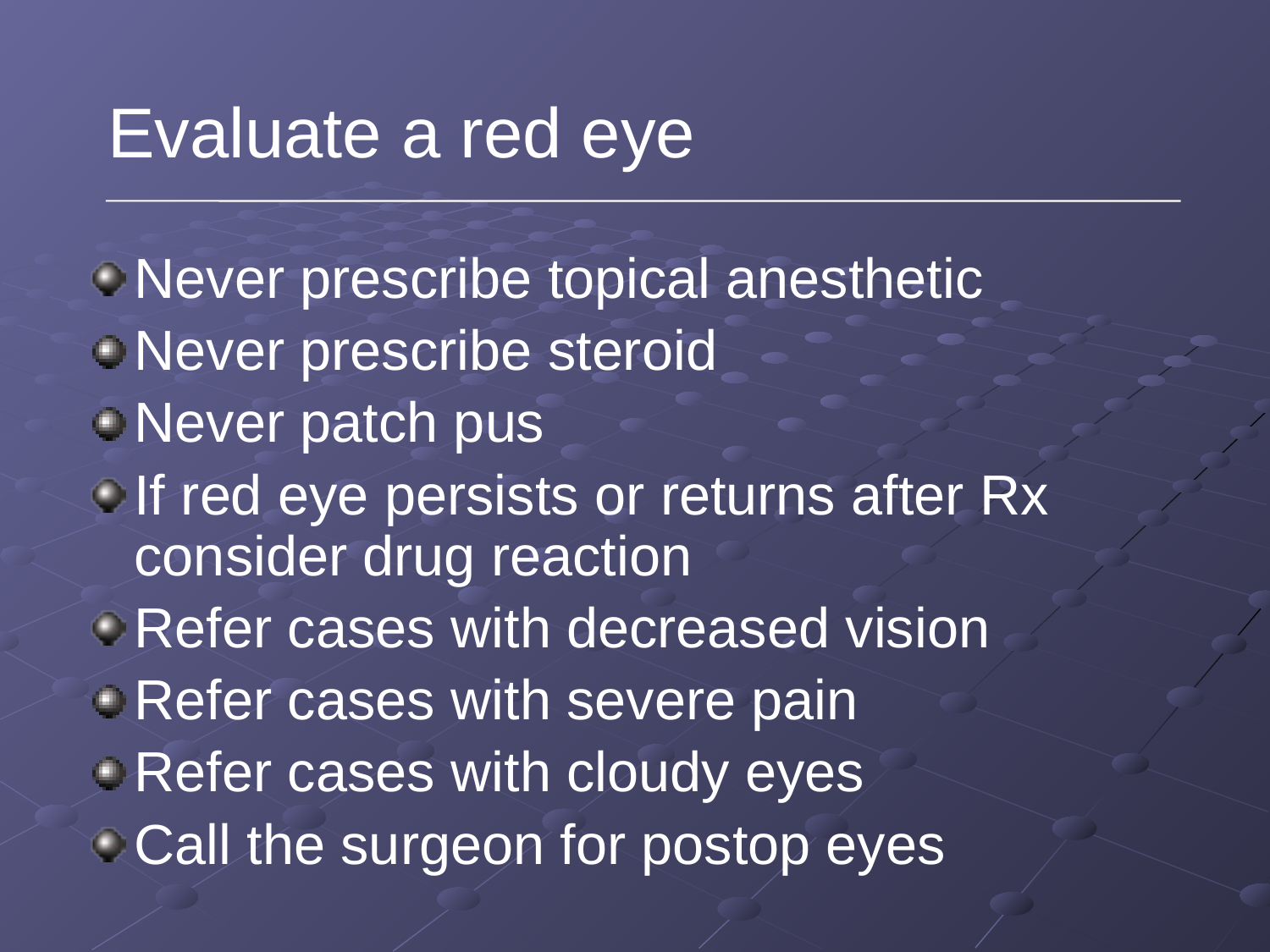

# Evaluate a red eye
Never prescribe topical anesthetic
Never prescribe steroid
Never patch pus
If red eye persists or returns after Rx consider drug reaction
Refer cases with decreased vision
Refer cases with severe pain
Refer cases with cloudy eyes
Call the surgeon for postop eyes

## Slide 32
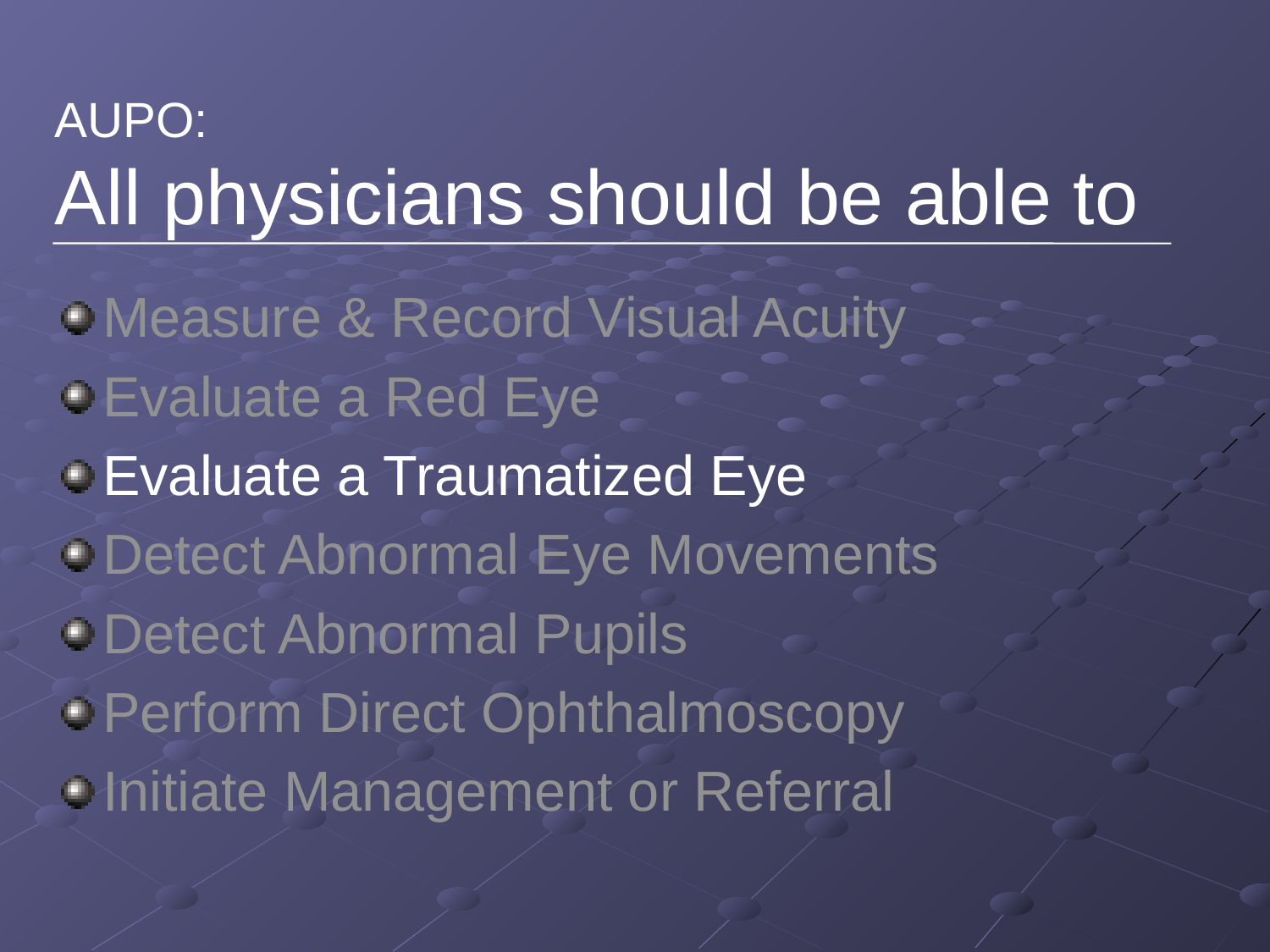

# AUPO: All physicians should be able to
Measure & Record Visual Acuity
Evaluate a Red Eye
Evaluate a Traumatized Eye
Detect Abnormal Eye Movements
Detect Abnormal Pupils
Perform Direct Ophthalmoscopy
Initiate Management or Referral

## Slide 33
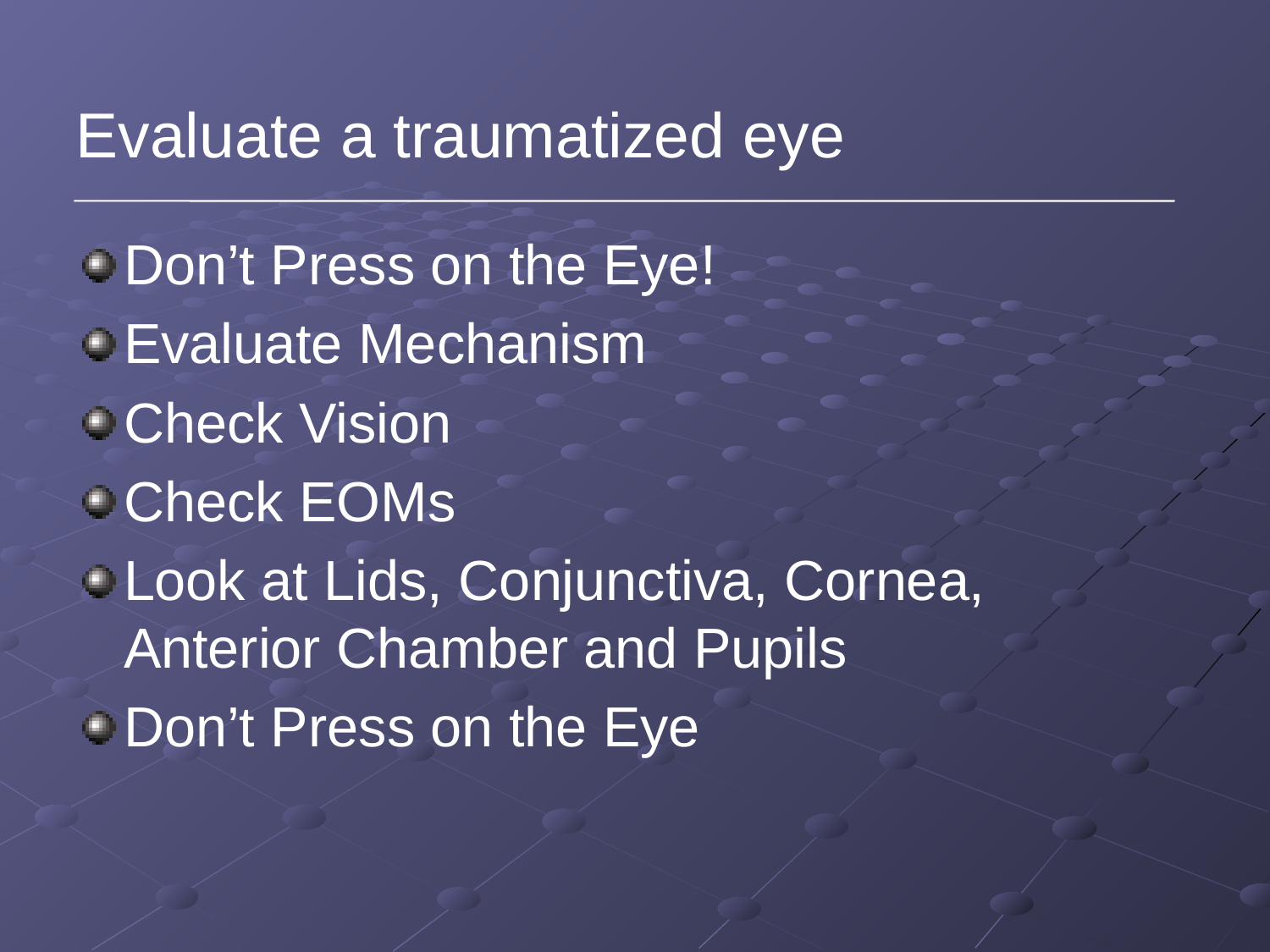

# Evaluate a traumatized eye
Don’t Press on the Eye!
Evaluate Mechanism
Check Vision
Check EOMs
Look at Lids, Conjunctiva, Cornea, Anterior Chamber and Pupils
Don’t Press on the Eye

## Slide 34
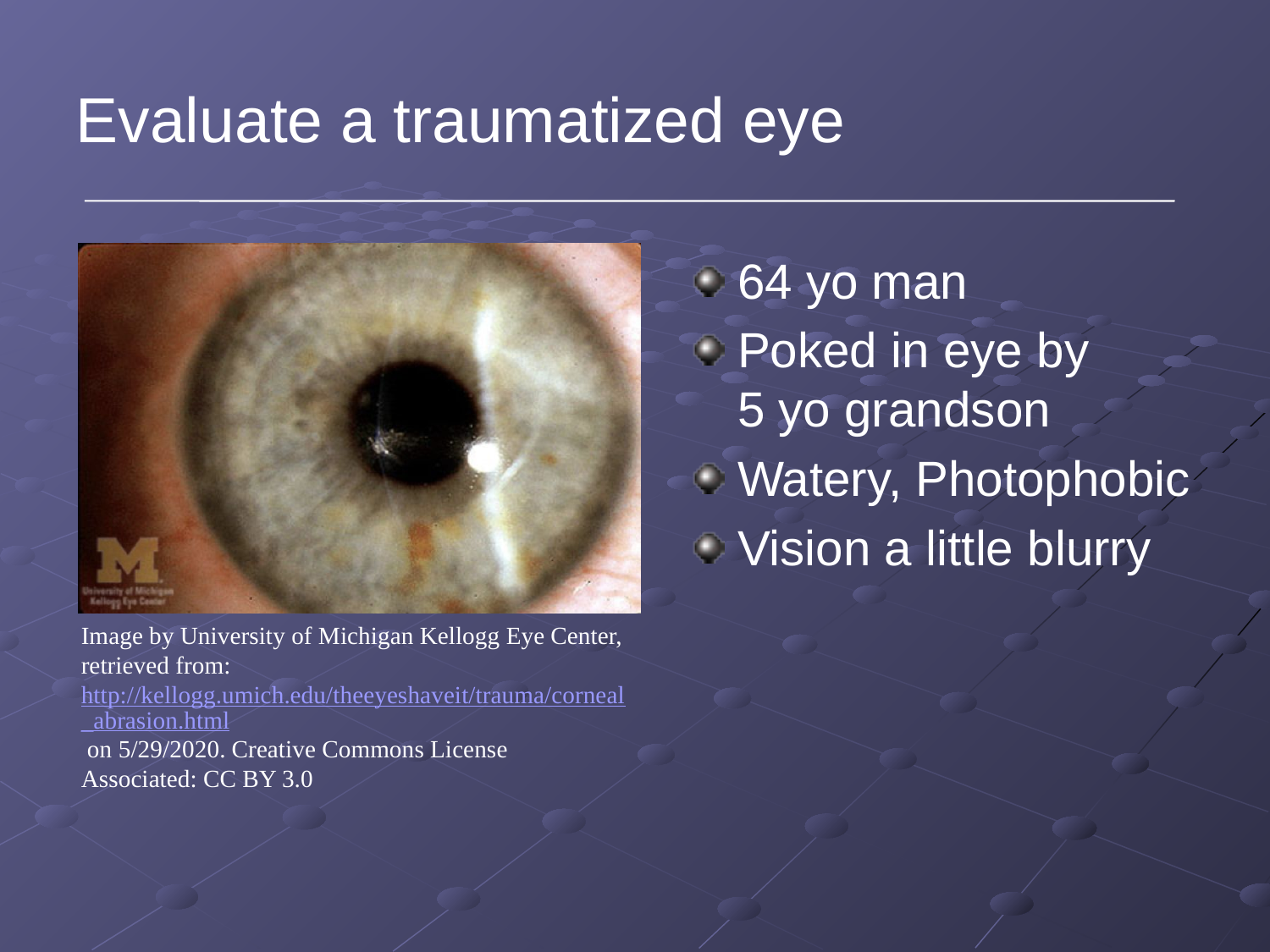

# Evaluate a traumatized eye
64 yo man
Poked in eye by
5 yo grandson
Watery, Photophobic
Vision a little blurry
Image by University of Michigan Kellogg Eye Center, retrieved from: http://kellogg.umich.edu/theeyeshaveit/trauma/corneal_abrasion.html on 5/29/2020. Creative Commons License Associated: CC BY 3.0

## Slide 35
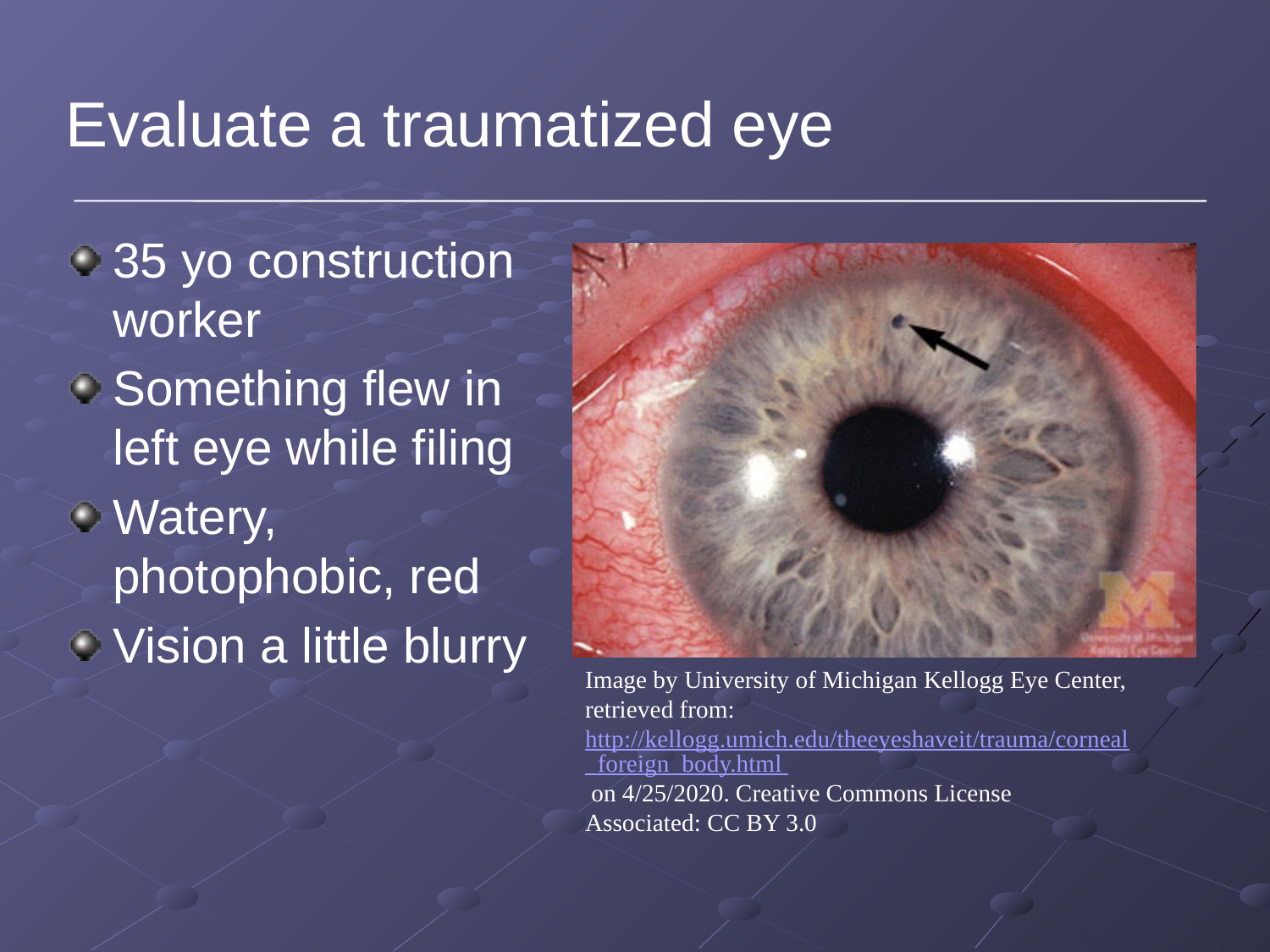

# Evaluate a traumatized eye
35 yo construction worker
Something flew in left eye while filing
Watery, photophobic, red
Vision a little blurry
Image by University of Michigan Kellogg Eye Center, retrieved from: http://kellogg.umich.edu/theeyeshaveit/trauma/corneal_foreign_body.html on 4/25/2020. Creative Commons License Associated: CC BY 3.0

## Slide 36
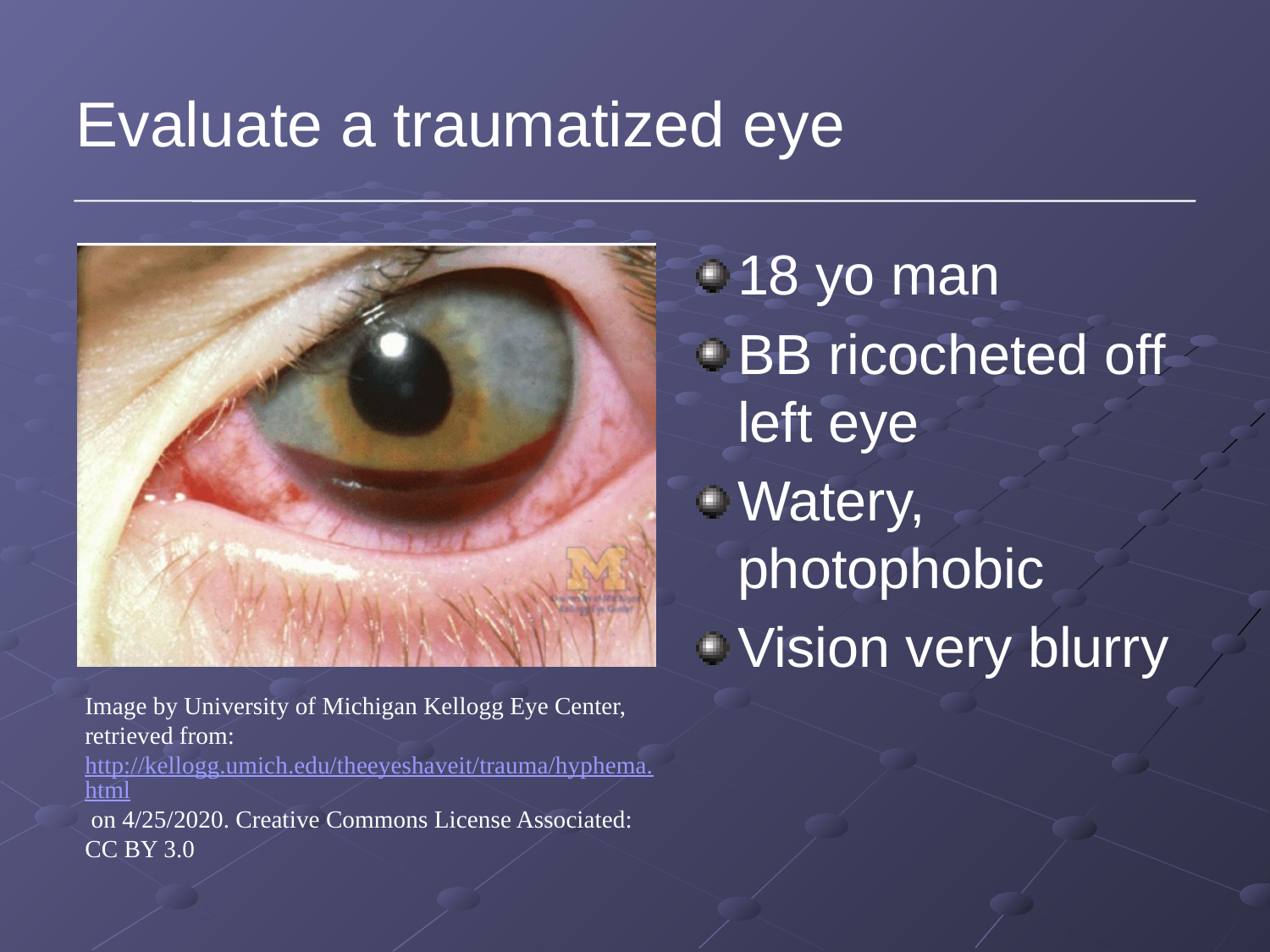

# Evaluate a traumatized eye
18 yo man
BB ricocheted off left eye
Watery, photophobic
Vision very blurry
Image by University of Michigan Kellogg Eye Center, retrieved from: http://kellogg.umich.edu/theeyeshaveit/trauma/hyphema.html on 4/25/2020. Creative Commons License Associated: CC BY 3.0

## Slide 37
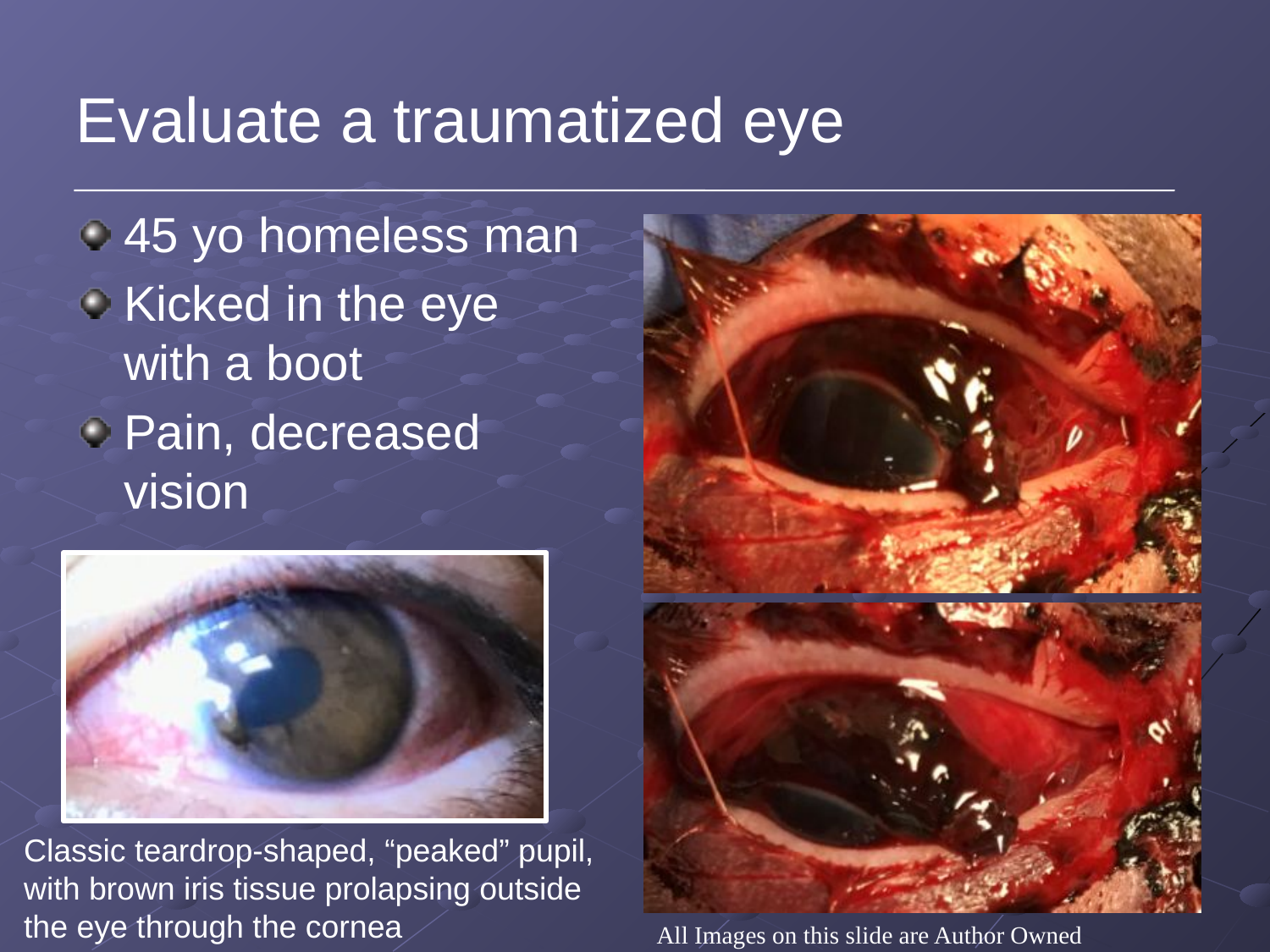

# Evaluate a traumatized eye
45 yo homeless man
Kicked in the eye with a boot
Pain, decreased vision
Classic teardrop-shaped, “peaked” pupil, with brown iris tissue prolapsing outside the eye through the cornea
All Images on this slide are Author Owned

## Slide 38
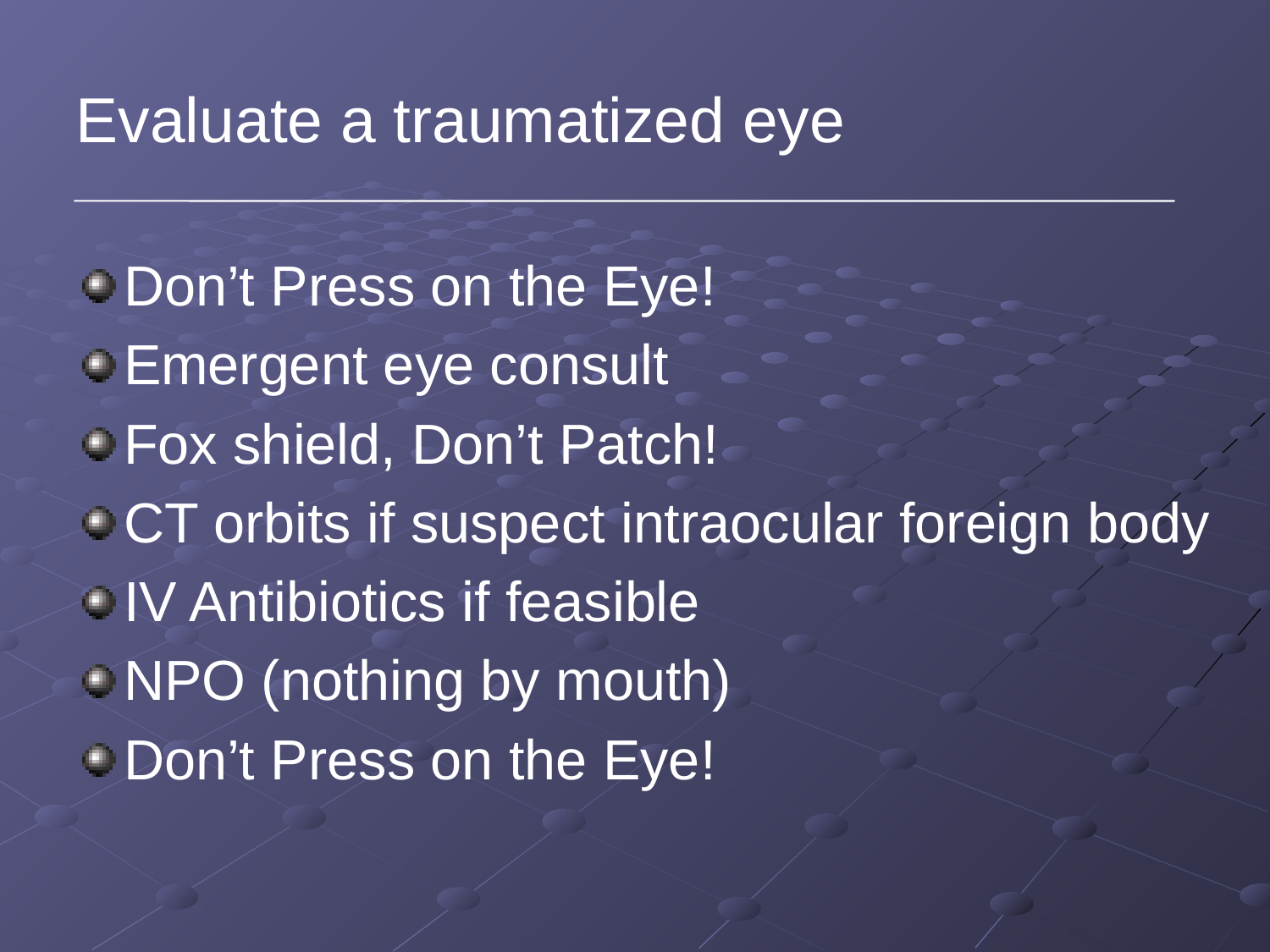

# Evaluate a traumatized eye
Don’t Press on the Eye!
Emergent eye consult
Fox shield, Don’t Patch!
CT orbits if suspect intraocular foreign body
IV Antibiotics if feasible
NPO (nothing by mouth)
Don’t Press on the Eye!

## Slide 39
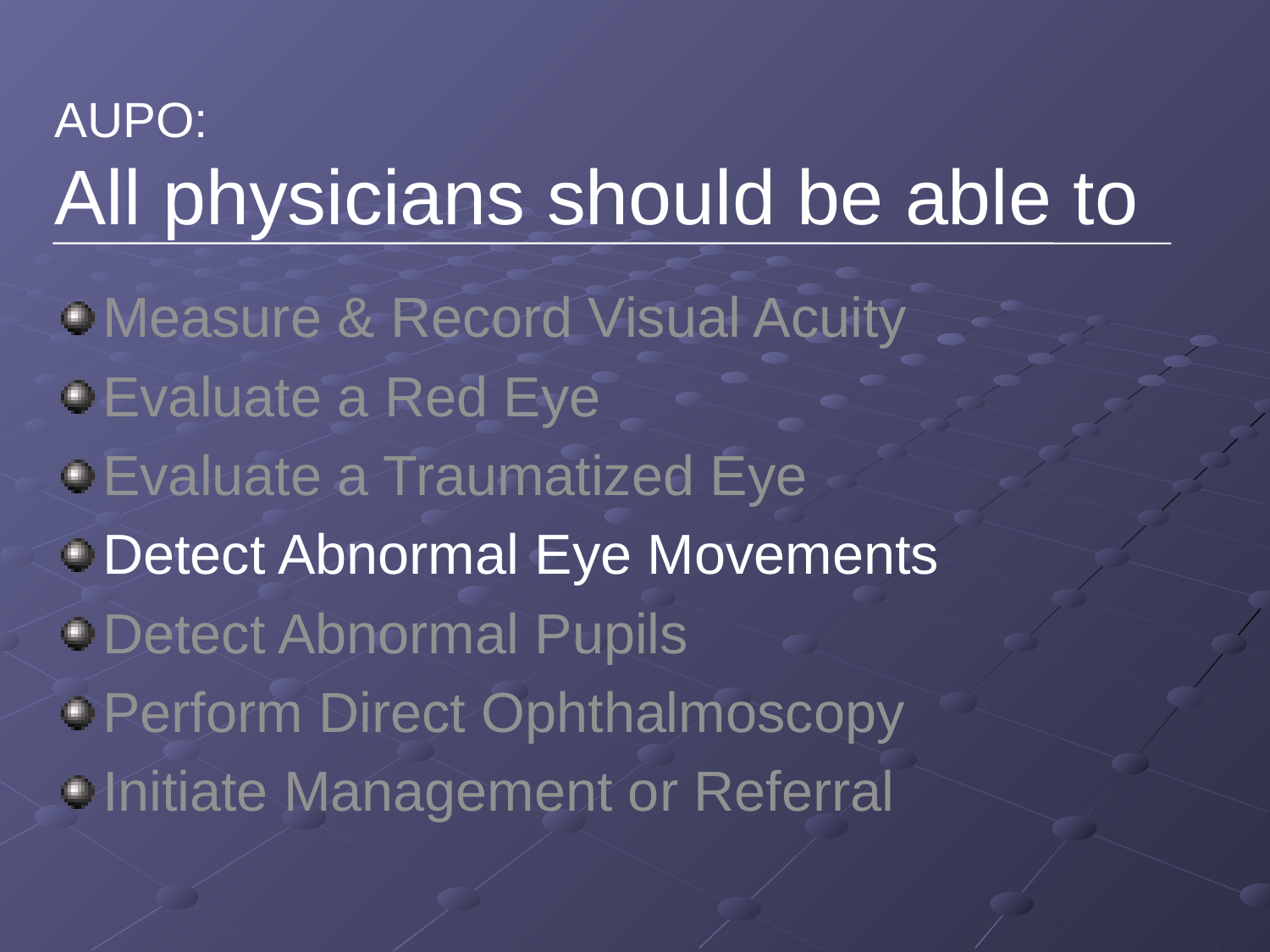

# AUPO: All physicians should be able to
Measure & Record Visual Acuity
Evaluate a Red Eye
Evaluate a Traumatized Eye
Detect Abnormal Eye Movements
Detect Abnormal Pupils
Perform Direct Ophthalmoscopy
Initiate Management or Referral

## Slide 40
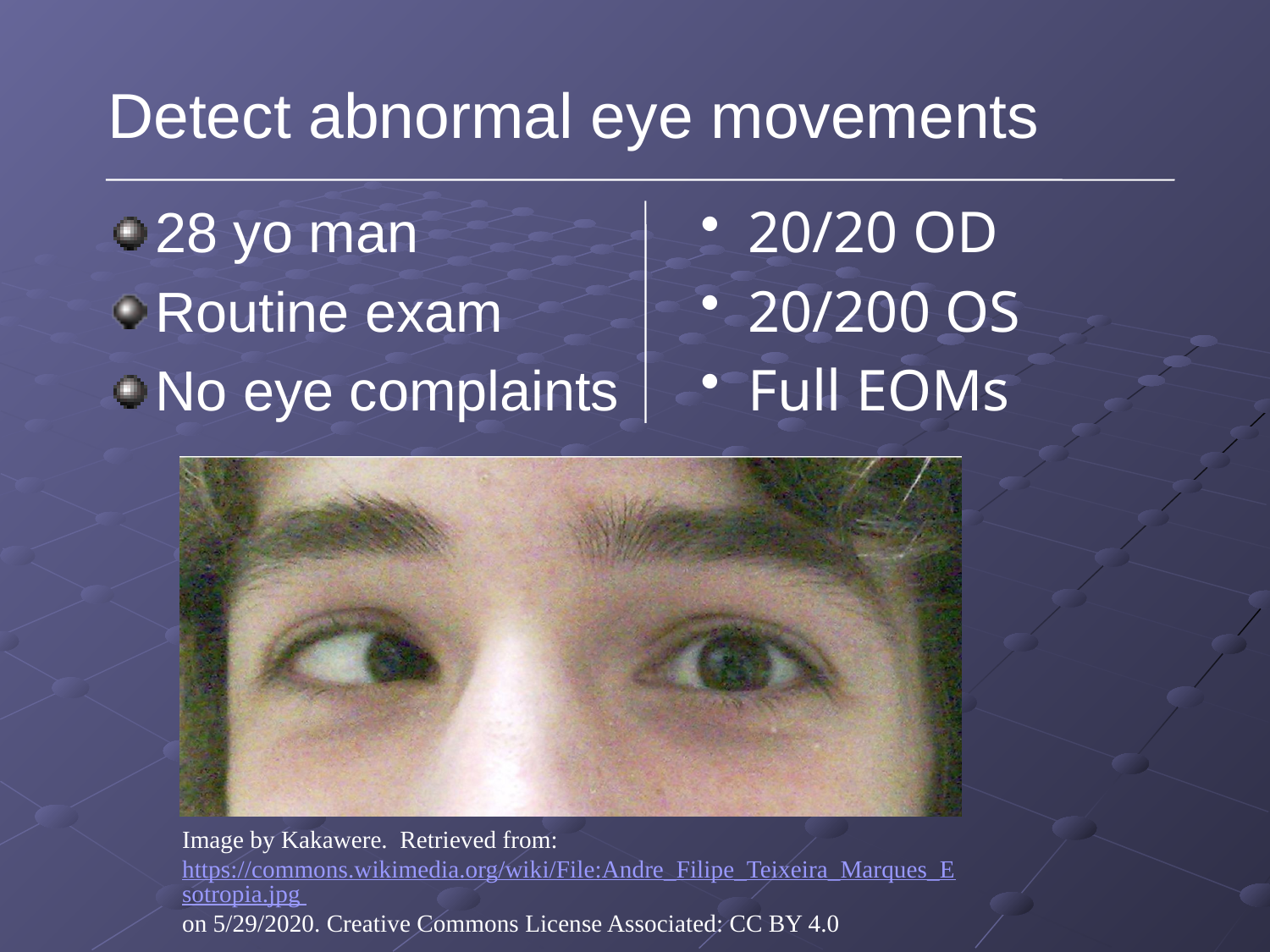

# Detect abnormal eye movements
28 yo man
Routine exam
No eye complaints
20/20 OD
20/200 OS
Full EOMs
Image by Kakawere. Retrieved from: https://commons.wikimedia.org/wiki/File:Andre_Filipe_Teixeira_Marques_Esotropia.jpg on 5/29/2020. Creative Commons License Associated: CC BY 4.0

## Slide 41
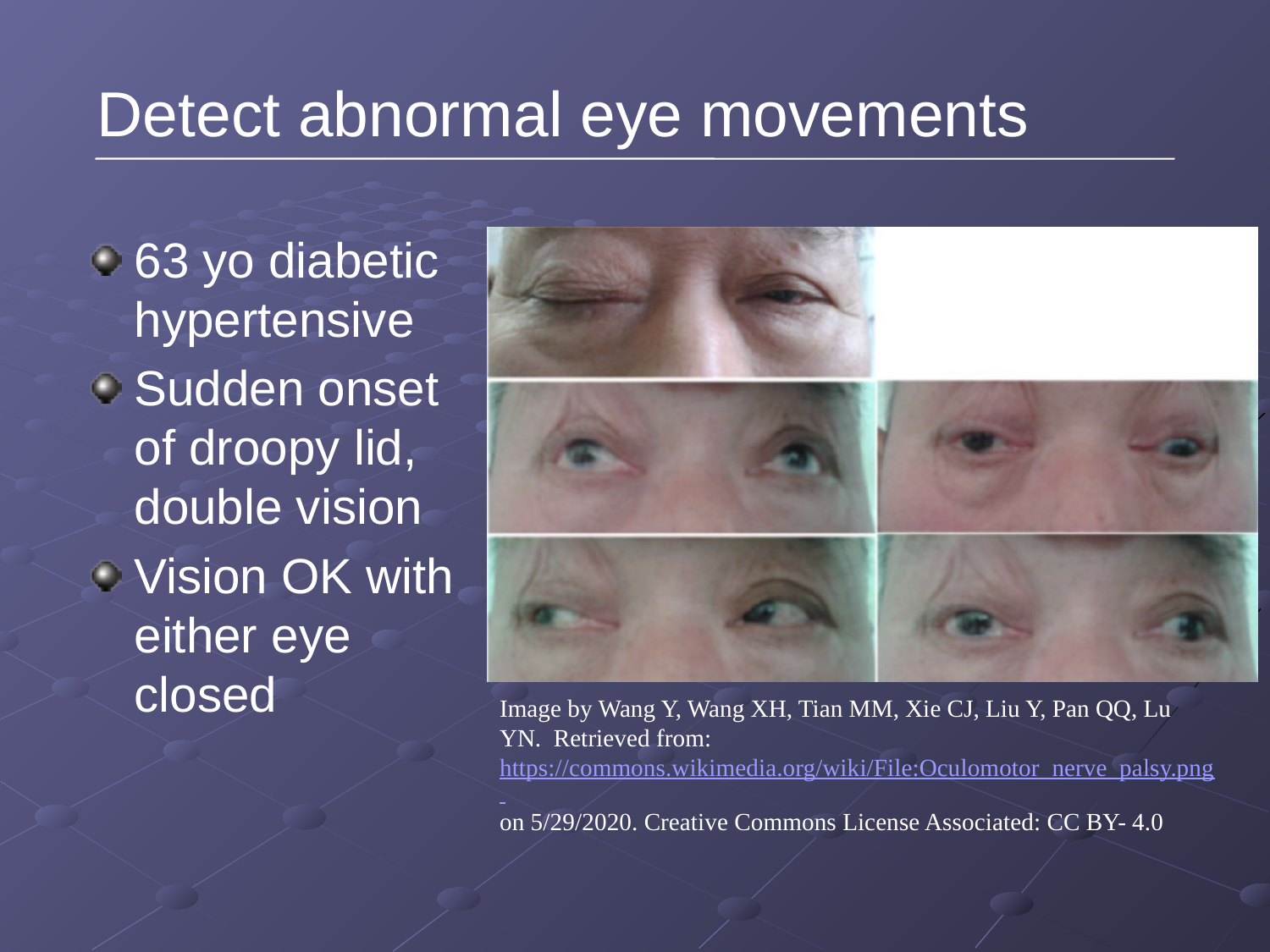

# Detect abnormal eye movements
63 yo diabetic hypertensive
Sudden onset of droopy lid, double vision
Vision OK with either eye closed
Image by Wang Y, Wang XH, Tian MM, Xie CJ, Liu Y, Pan QQ, Lu YN. Retrieved from: https://commons.wikimedia.org/wiki/File:Oculomotor_nerve_palsy.png on 5/29/2020. Creative Commons License Associated: CC BY- 4.0

## Slide 42
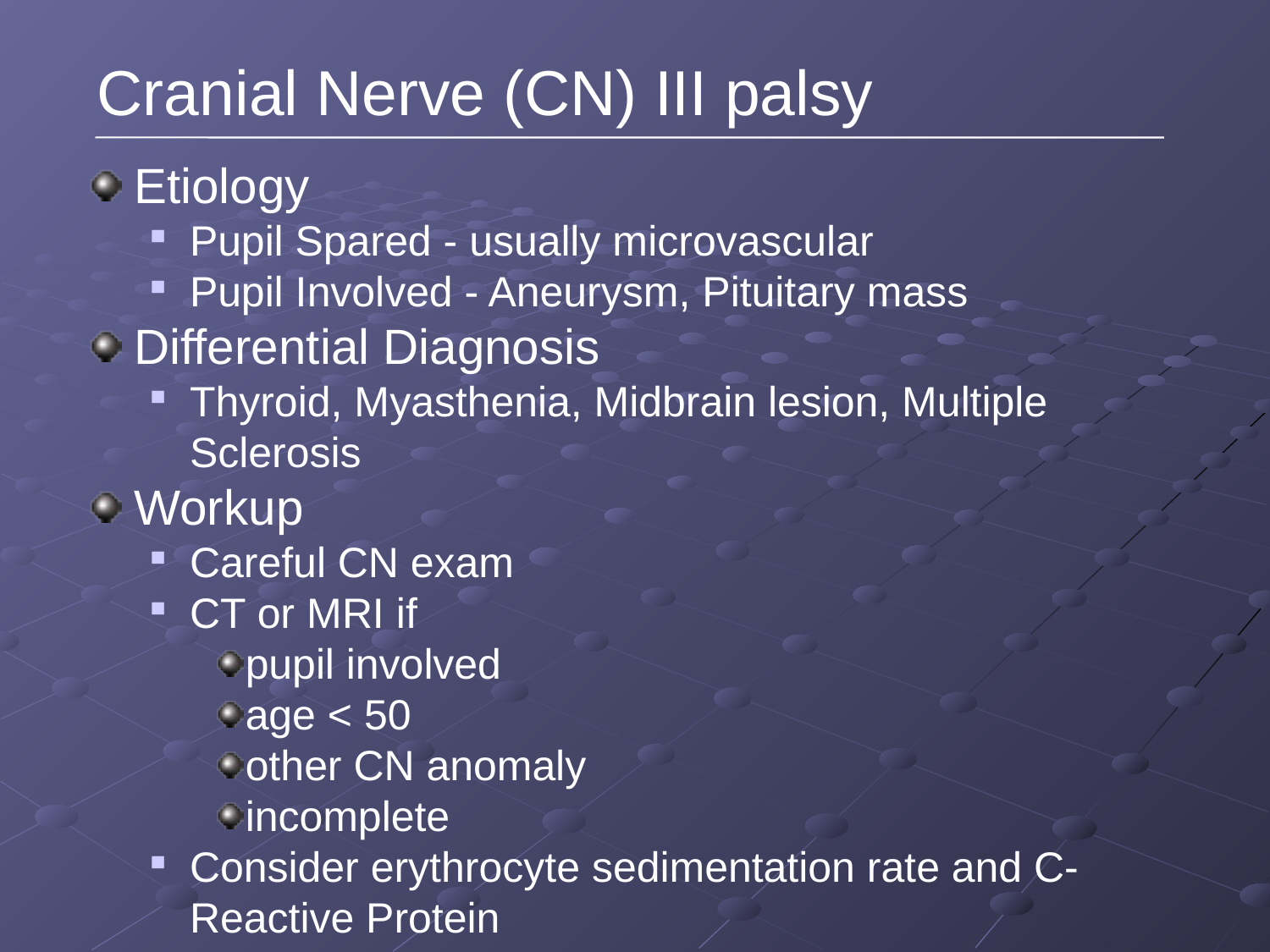

# Cranial Nerve (CN) III palsy
Etiology
Pupil Spared - usually microvascular
Pupil Involved - Aneurysm, Pituitary mass
Differential Diagnosis
Thyroid, Myasthenia, Midbrain lesion, Multiple Sclerosis
Workup
Careful CN exam
CT or MRI if
pupil involved
age < 50
other CN anomaly
incomplete
Consider erythrocyte sedimentation rate and C-Reactive Protein

## Slide 43
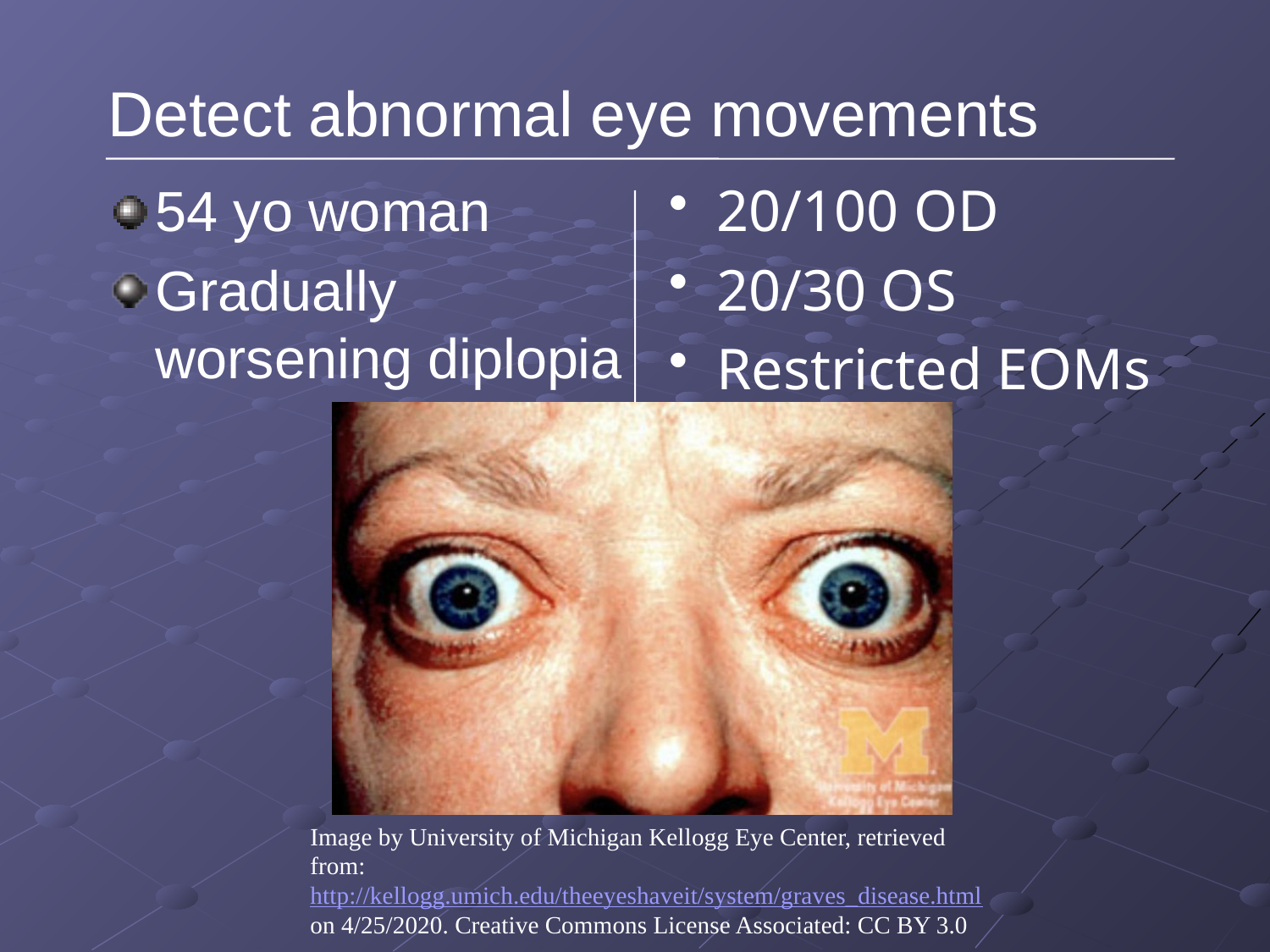

# Detect abnormal eye movements
54 yo woman
Gradually worsening diplopia
20/100 OD
20/30 OS
Restricted EOMs
Image by University of Michigan Kellogg Eye Center, retrieved from: http://kellogg.umich.edu/theeyeshaveit/system/graves_disease.html on 4/25/2020. Creative Commons License Associated: CC BY 3.0

## Slide 44
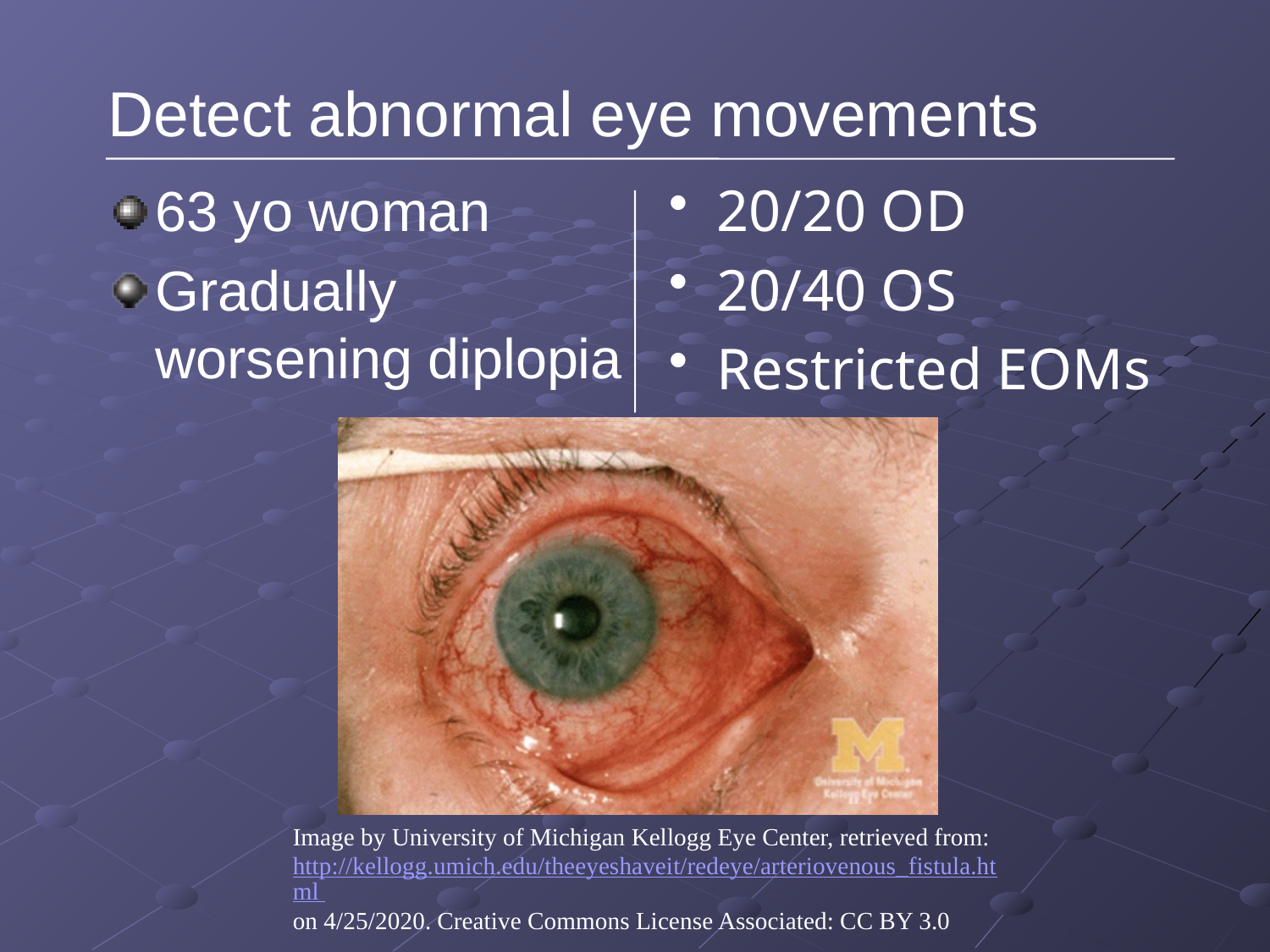

# Detect abnormal eye movements
63 yo woman
Gradually worsening diplopia
20/20 OD
20/40 OS
Restricted EOMs
Image by University of Michigan Kellogg Eye Center, retrieved from: http://kellogg.umich.edu/theeyeshaveit/redeye/arteriovenous_fistula.html on 4/25/2020. Creative Commons License Associated: CC BY 3.0

## Slide 45
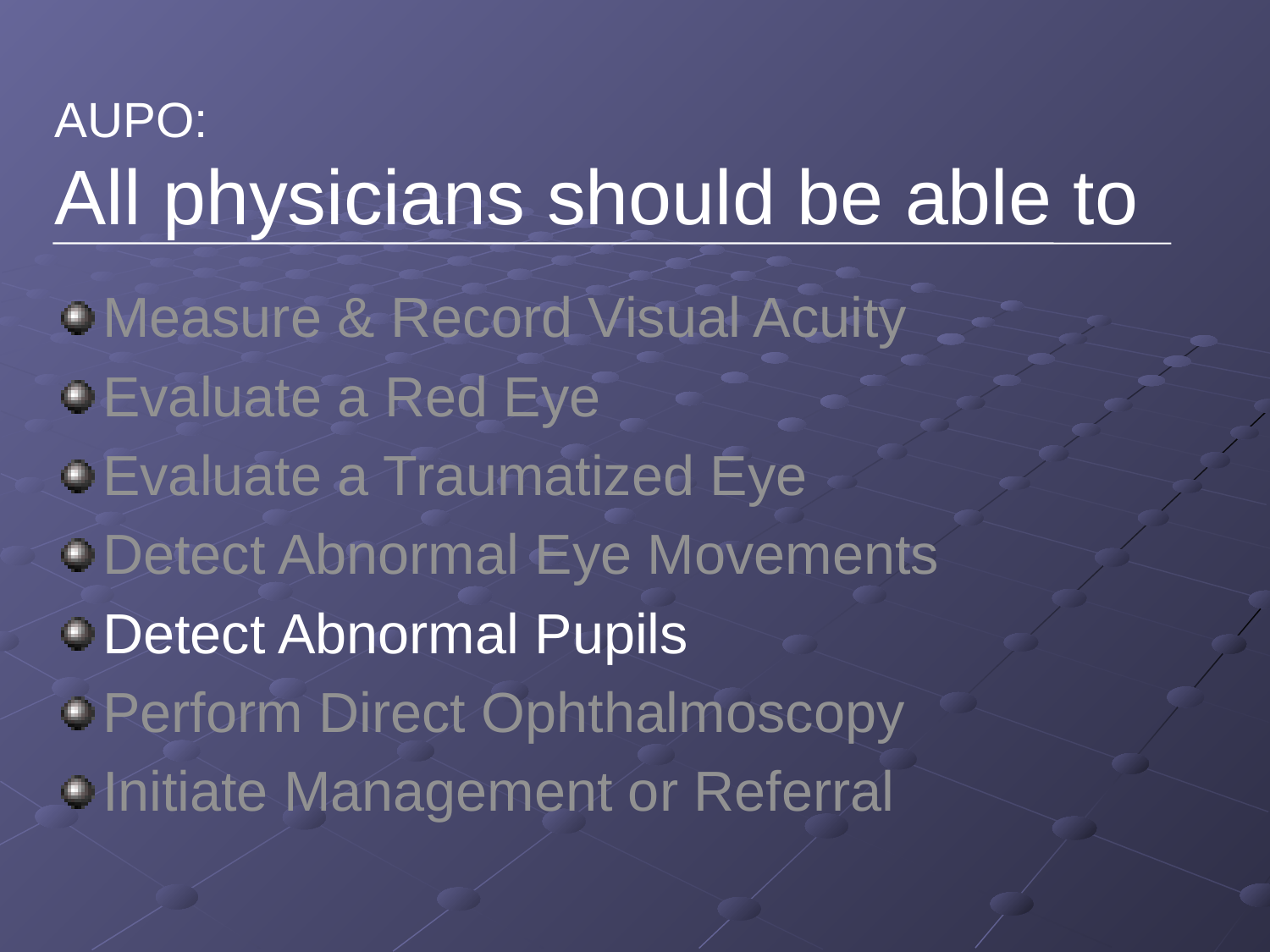

# AUPO: All physicians should be able to
Measure & Record Visual Acuity
Evaluate a Red Eye
Evaluate a Traumatized Eye
Detect Abnormal Eye Movements
Detect Abnormal Pupils
Perform Direct Ophthalmoscopy
Initiate Management or Referral

## Slide 46
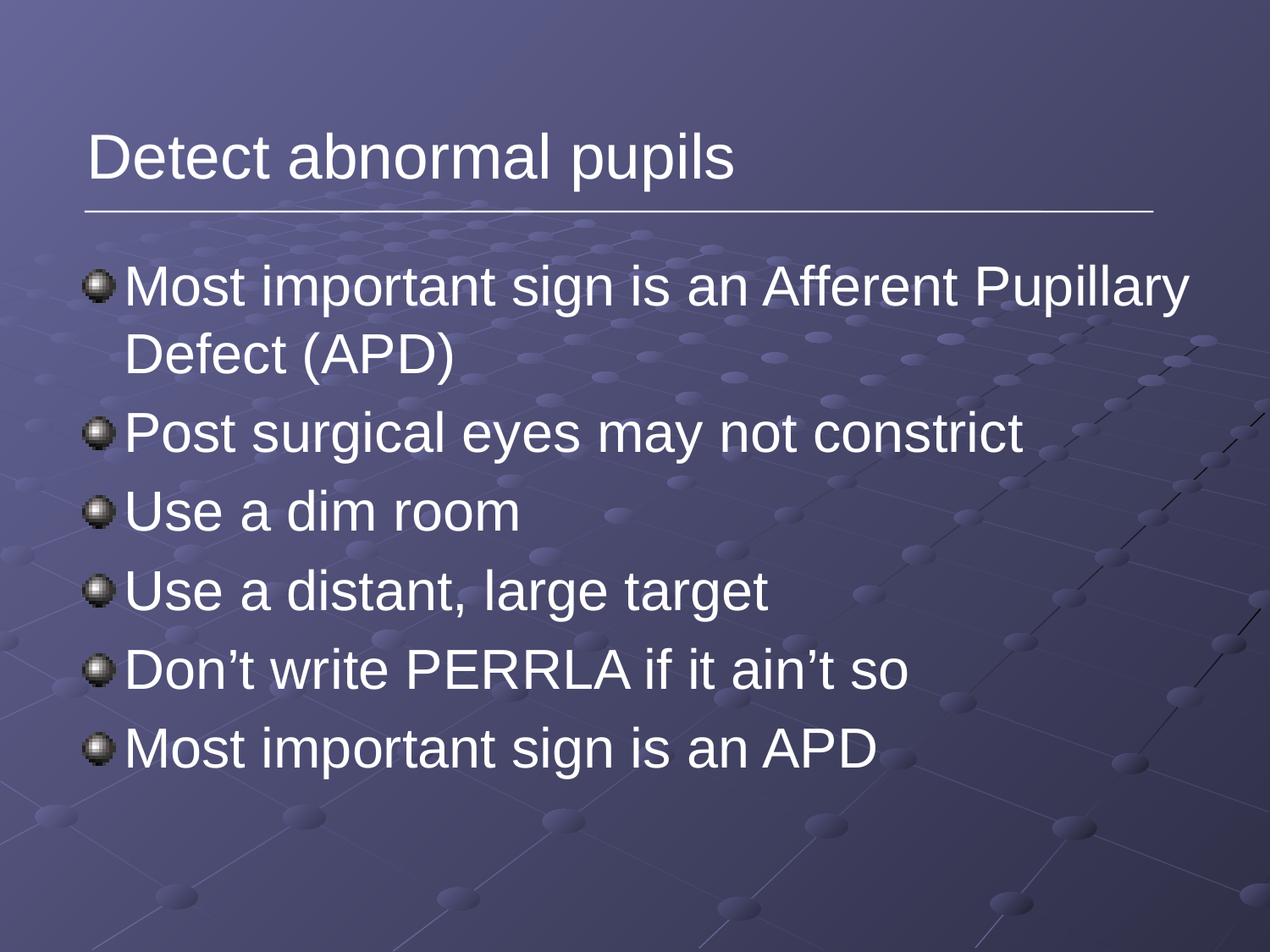

# Detect abnormal pupils
Most important sign is an Afferent Pupillary Defect (APD)
Post surgical eyes may not constrict
Use a dim room
Use a distant, large target
Don’t write PERRLA if it ain’t so
Most important sign is an APD

## Slide 47
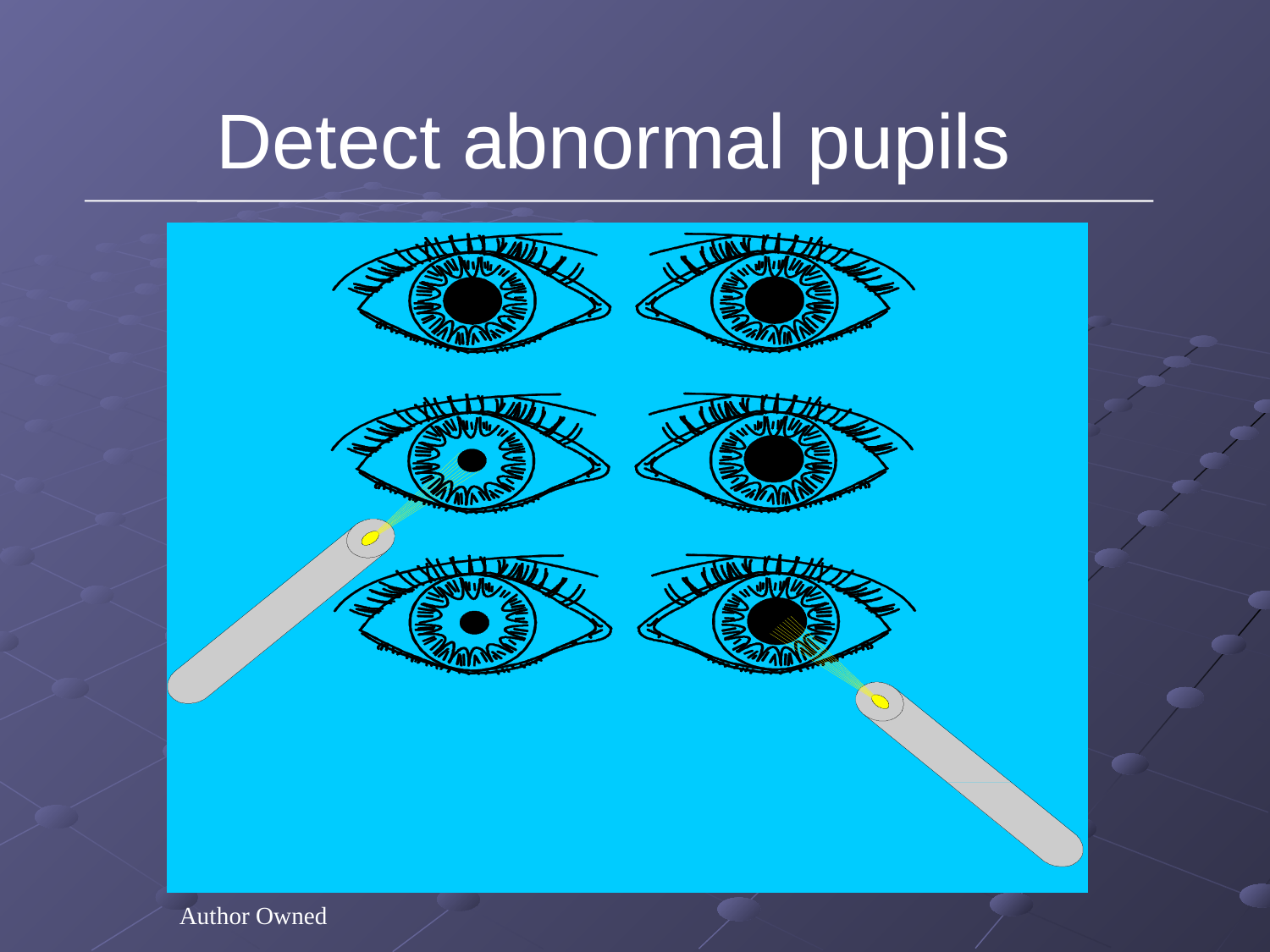

# Detect abnormal pupils
Author Owned

## Slide 48
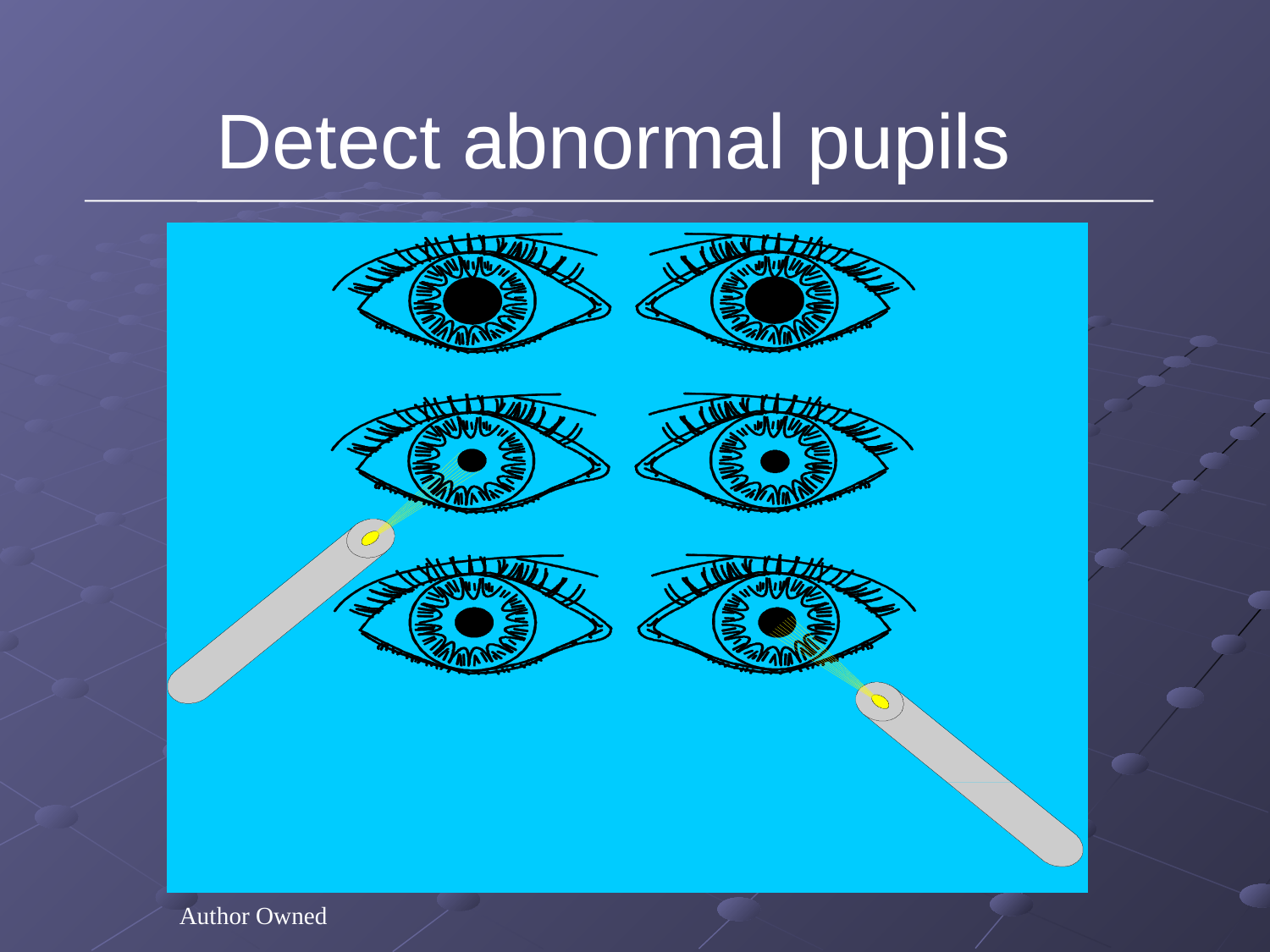

# Detect abnormal pupils
Author Owned

## Slide 49
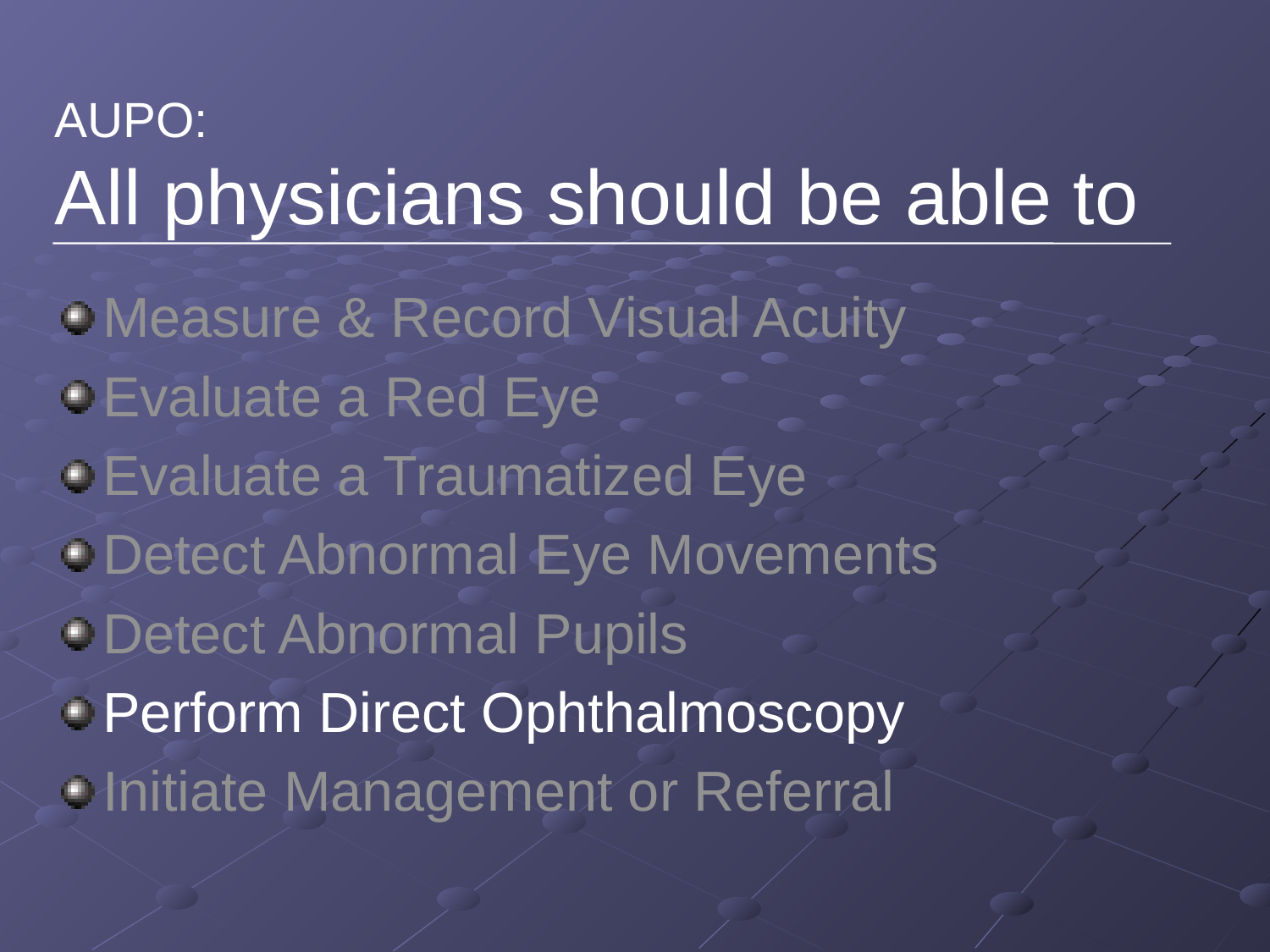

# AUPO: All physicians should be able to
Measure & Record Visual Acuity
Evaluate a Red Eye
Evaluate a Traumatized Eye
Detect Abnormal Eye Movements
Detect Abnormal Pupils
Perform Direct Ophthalmoscopy
Initiate Management or Referral

## Slide 50
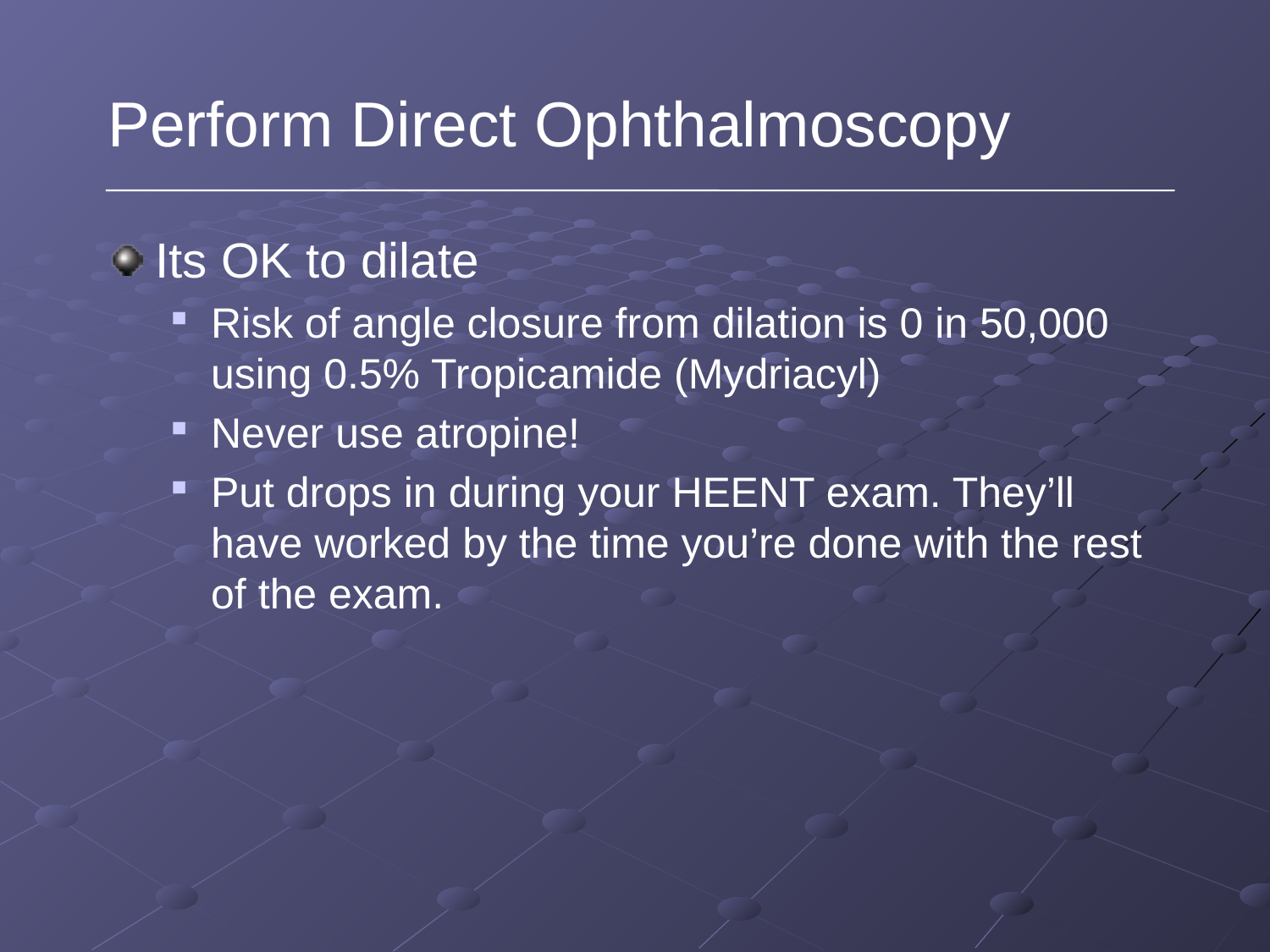

# Perform Direct Ophthalmoscopy
Its OK to dilate
Risk of angle closure from dilation is 0 in 50,000 using 0.5% Tropicamide (Mydriacyl)
Never use atropine!
Put drops in during your HEENT exam. They’ll have worked by the time you’re done with the rest of the exam.

## Slide 51
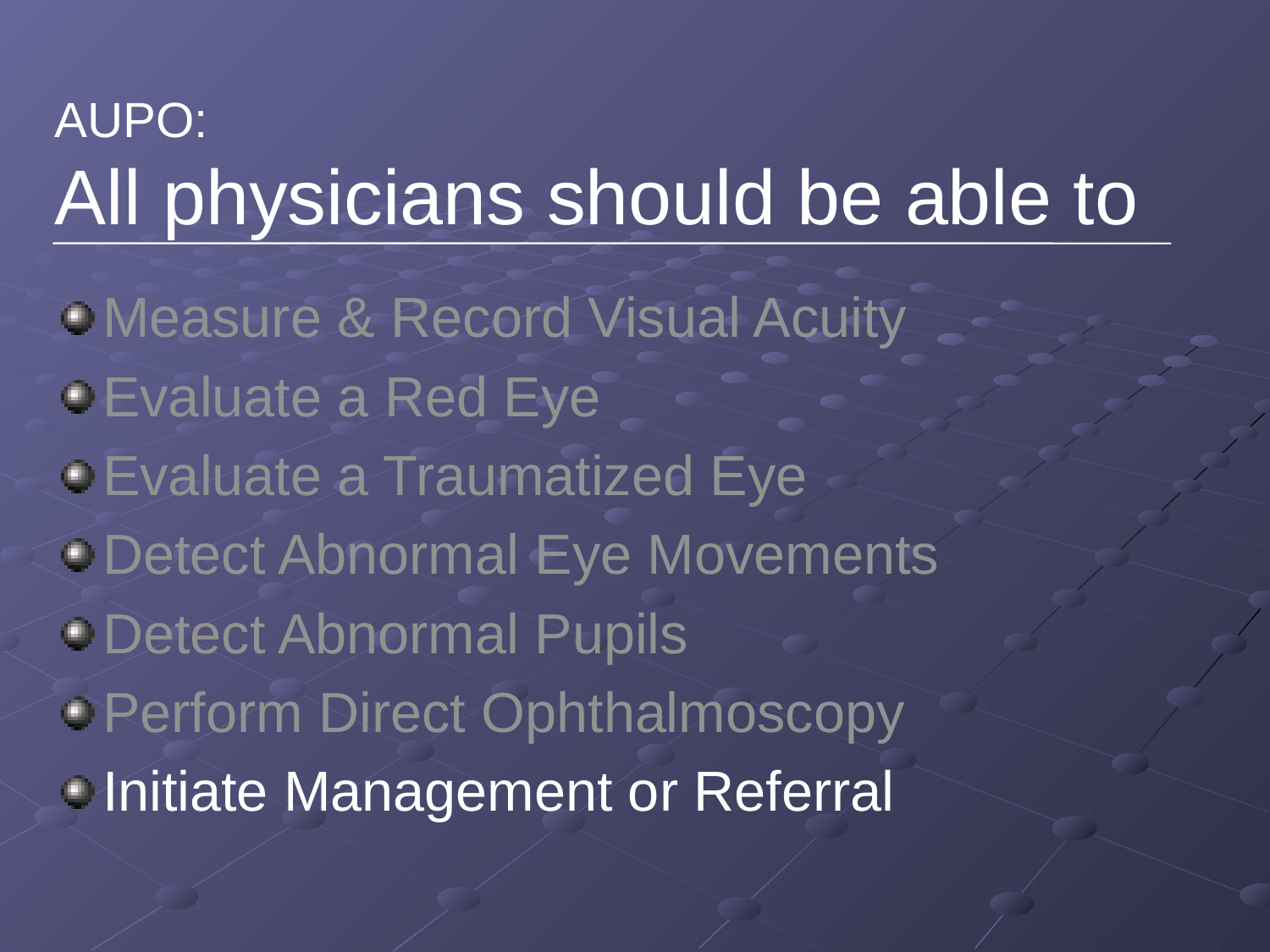

# AUPO: All physicians should be able to
Measure & Record Visual Acuity
Evaluate a Red Eye
Evaluate a Traumatized Eye
Detect Abnormal Eye Movements
Detect Abnormal Pupils
Perform Direct Ophthalmoscopy
Initiate Management or Referral

## Slide 52
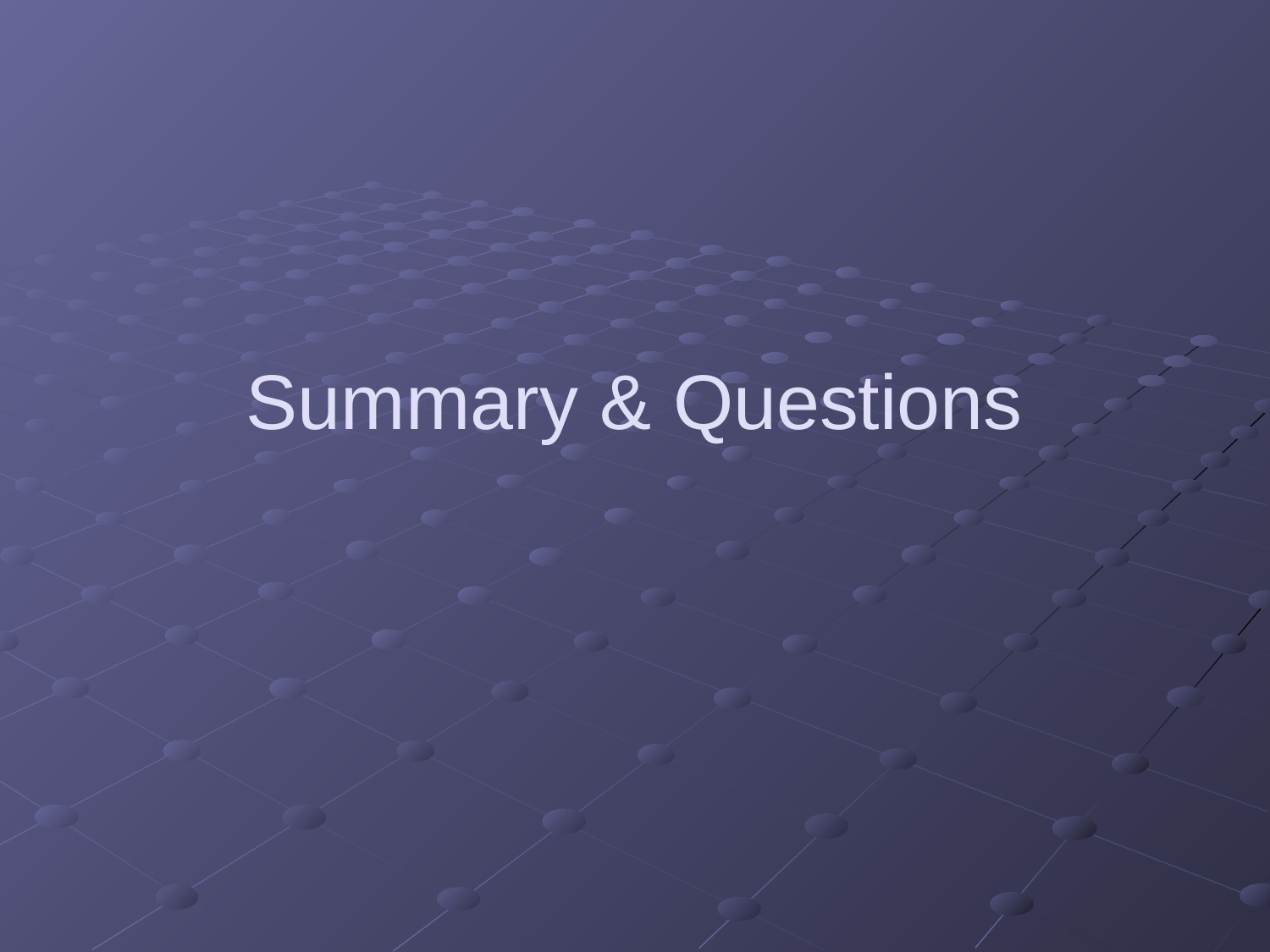

# Summary & Questions
