# Supplementary material for: A Required Ophthalmology Rotation: Providing Medical Students with a Foundation in Eye-Related Diagnoses and Management
Source: MedEdPORTAL. 2021 Feb 12;17:11100. doi: 10.15766/mep_2374-8265.11100 (PMC7880261; doi:10.15766/mep_2374-8265.11100)
Supplement: Supplementary file 1 — Ophthalmology Slides Instructors Guide.docxOphthalmology Handout.docxOphthalmology Slides.pptxOphthalmology Sessions.docxOphthalmology Sessions Answer Key.docxOphthalmology Sessions Student Handouts.docxOphthalmology Final Examination.docxStudent Postrotation Feedback Form.docx [file mep_2374-8265.11100-s001.zip › D. Ophthalmology Sessions.docx]

**Cataract Case Conference**

**Case 1** - A 65-year-old man complains of difficulty seeing street signs while driving and also of glare from headlights that interferes with driving at night. On exam, his visual acuity with his current glasses is 20/50 OD (right) and 20/40 OS (left).


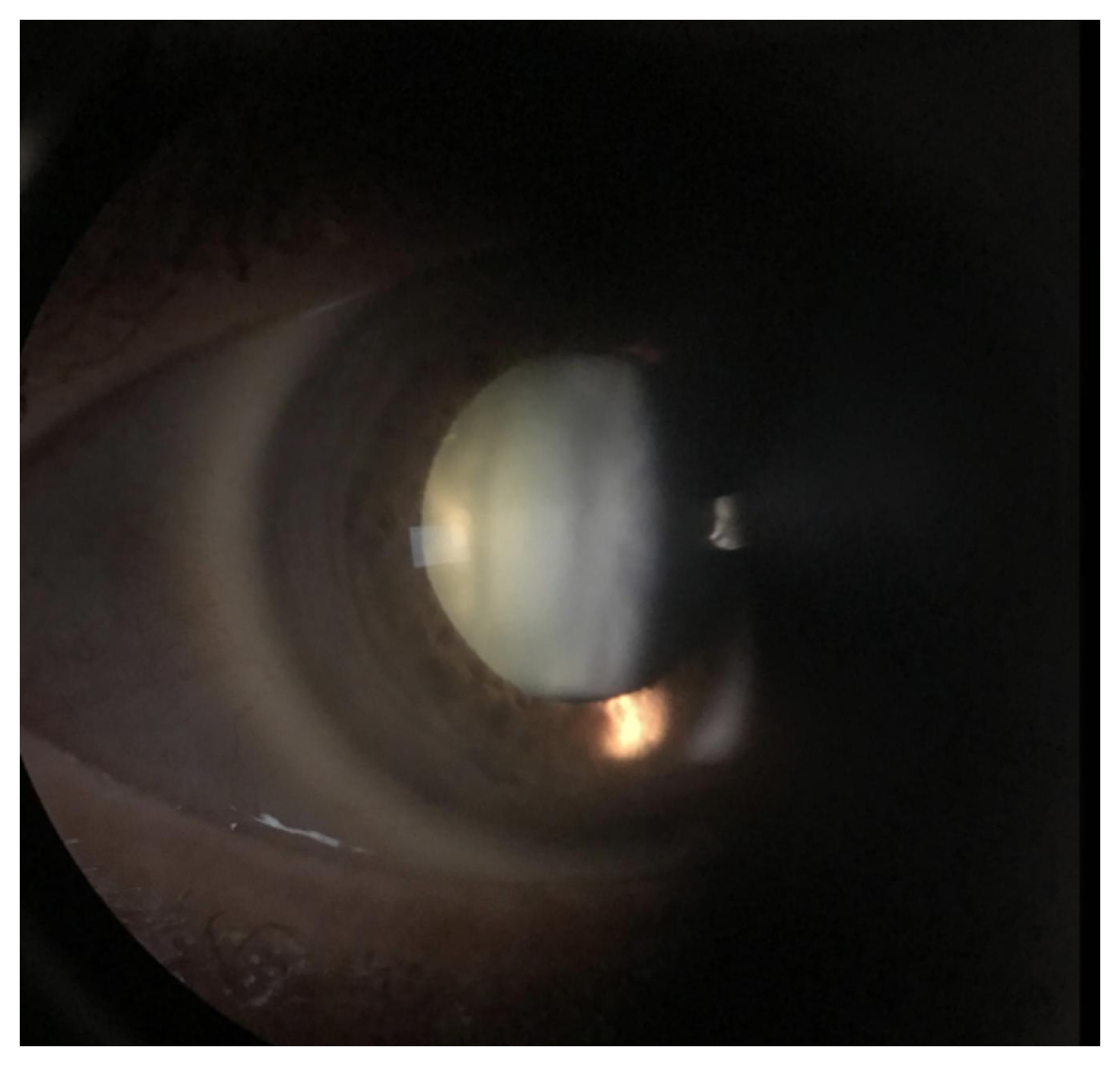


1. Describe the findings.
2. What is the diagnosis?
3. What other elements of the eye exam are important in deciding on management?
4. Further exam demonstrates that the vision is unimproved

with a change in glasses prescription and that the remainder of the exam is normal. Discuss the medical and surgical management options. Be sure to compare the risks of further observation versus the risk of cataract surgery.

Author Owned

1. The patient asks for more detail on how cataract surgery is done and what he can expect during the surgery. In particular, he wants to know how the laser is used and how often he’ll need to have his implant replaced. Also, how long will the eye be removed from the socket?

**Case 2** - A 72-year-old man is brought in by his wife for a second opinion regarding cataracts. He had recently seen another doctor who told them that he needed to have his cataracts removed as soon as possible. The patient does not drive and has no complaints about his vision. His current medical history is significant for Alzheimer’s disease, hypertension, and diabetes.

On exam, his visual acuity with his current glasses is 20/50 OD and 20/40 OS. Vision is unimproved with a change in glasses prescription. Anterior segment exam show 2+ nuclear sclerotic cataracts. Examination of the retina reveals some changes consistent with dry macular degeneration.

1. Discuss the medical and surgical management options. Make sure to include the indications for cataract surgery. When is cataract surgery “needed as soon as possible?”
2. What is your advice to the patient and his wife?
3. Would this advice be different if the patient’s vision was 20/400 in each eye and the wife noted that the patient was having trouble getting around the house?

**Case 3** - You are the acting intern on the neo-natal unit. As you are examining a 1-day old baby girl you notice the finding below.


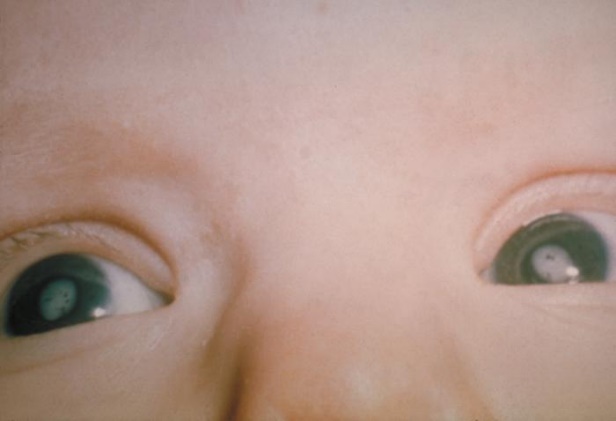


Image by Centers for Disease Control and Prevention, retrieved from: <https://phil.cdc.gov/phil_images/20030724/28/PHIL_4284_lores.jpg> on 4/12/2020. Creative Commons License associated: CC0 1.0

1. Describe the finding.
2. What are the possible diagnoses? Which is most likely?
3. Assuming that this is bilateral cataracts, what are the management options? What are the indications for cataract surgery?

**Glaucoma Case Conference**

**Case 1** - A 46-year-old man comes to your office for a routine physical.

1. What elements of the pre-examination history would be relevant to his risk for glaucoma?
2. Describe the parts of the eye examination that can be done in the primary care setting to further assess this patient’s glaucoma risk.

On history, the patient notes that he has no eye problems and that his glasses for myopia work well. He was hit in the right eye by a baseball when he was a teenager but after a few weeks he saw well and has had no problems since. His mother takes eye drops but he does not know the details.

On examination you note visual acuity of 20/20 with glasses in both eyes. On checking the pupils you notice a small amount of redilation of the pupils of both eyes when the light is moved from the left eye to the right eye and a small amount of constriction of both pupils when the light is moved from the right eye to the left eye. Exam of the right eye with the direct ophthalmoscope is shown below.


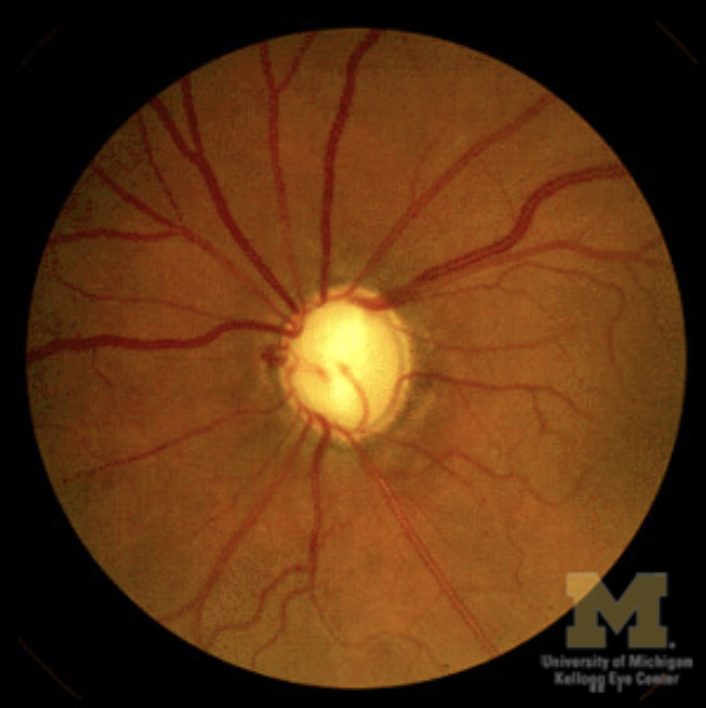


Image by University of Michigan Kellogg Eye Center, retrieved from: <http://kellogg.umich.edu/theeyeshaveit/opticfundus/disc_cupping.html>

on 5/29/2020. Creative Commons License Associated: CC BY 3.0

1. Describe the findings.
2. What are the likely diagnoses?
3. Describe further management in the primary care setting. Is this an emergent, urgent or routine problem?
4. Describe management by the ophthalmic consultant.

**Case 2** - A 59-year-old woman comes to the emergency dept


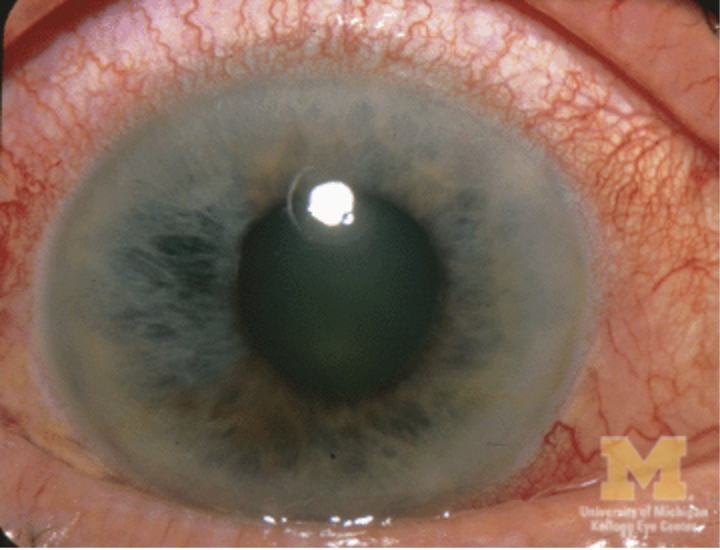


complaining of severe right eye pain beginning this morning.

The pain has been getting worse through the day and the vision

in that eye has been getting progressively foggy and dim.

She has no previous eye history except for wearing glasses.

She’s had a cold recently for which she’s been taking an over

the counter cold remedy.

Image by University of Michigan Kellogg Eye Center, retrieved from: <http://kellogg.umich.edu/theeyeshaveit/redeye/angleclosure_glaucoma.html> on 5/29/2020. Creative Commons License Associated: CC BY 3.0

1. What additional historical details might be helpful?
2. Describe the parts of the eye examination that can be done in the ED setting to further assess this patient’s problem.

On review of additional history, the patient tells you that she has needed her glasses for reading more than for distance. The cold medicine she was taking has pseudophedrine in it. She remembers driving her aunt to an appointment for laser surgery a long time ago.

On examination, the vision is 20/400 on the right and 20/20 on the left. While the pupil response on the right is normal, the left pupil appears fixed and mid-dilated. The right eye appears normal to a penlight exam but the cornea on the left is cloudy looking. Gentle palpation of the eyes shows that the left eye is much harder than the right. Direct ophthalmoscopy is unrewarding.

1. What is the likely diagnosis?
2. What steps can be taken in the ED setting to manage this patient? Is this an emergent, urgent or routine problem?
3. Describe management by the ophthalmic consultant.

**Case 3** – A 2-year-old boy is brought to the pediatrician. The mother notes that the left eye doesn’t look right and tears a lot.


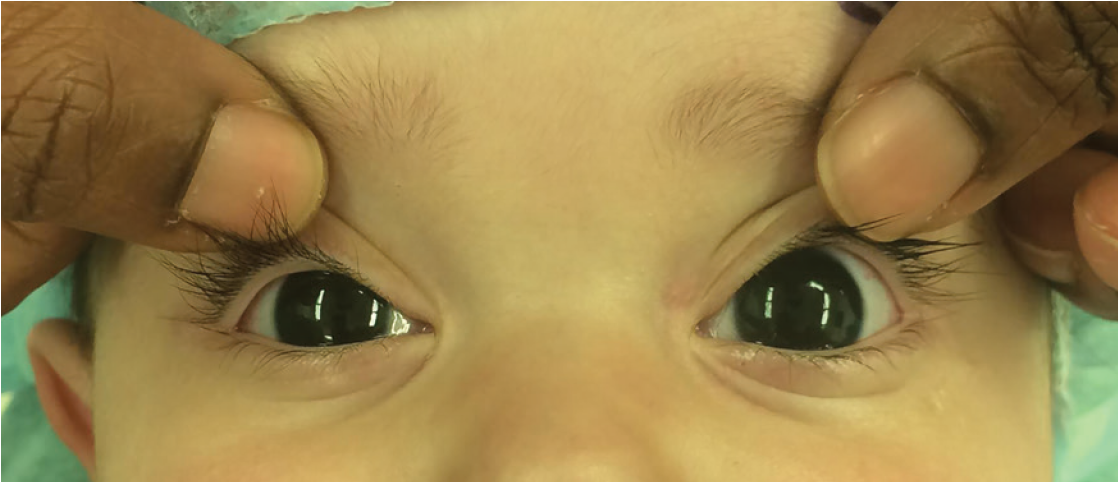


1. What diagnosis needs to be considered?
2. Describe further management for this condition in the primary care setting. Is this an emergent, urgent or routine problem?

Image by American Academy of Ophthalmology, used with permission

1. Describe management by an ophthalmologist in present day.

**Macular Degeneration Case Conference**

**Case 1** - A 72-year-old woman has noticed distortion of her vision over the last month. Everything appears normal when only the right eye is opened. When the left eye is open straight lines appear to be bent in the middle.

1. What elements of the eye exam can be performed in the primary care setting to determine the cause of the visual loss?

The retinal exams of the left and right eyes are shown below.


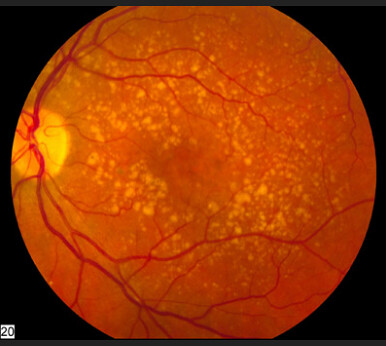


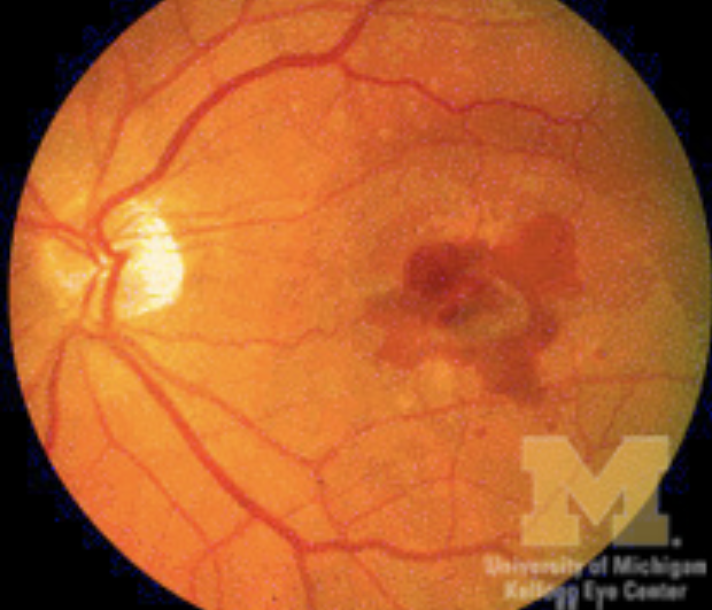


Image by National Eye Institute, retrieved from: <https://search.creativecommons.org/photos/5b566129-05d3-4204-997b-2ef9f6c14379> on 4/13/2020. Creative Commons License associated:  [CC BY 2.0](https://creativecommons.org/licenses/by-nc-nd/2.0/?ref=ccsearch&atype=rich)

Image by University of Michigan Kellogg Eye Center, retrieved from: <http://kellogg.umich.edu/theeyeshaveit/opticfundus/subretinal_hemorrhage.html>

on 5/29/2020. Creative Commons License Associated: CC BY 3.0

1. Describe the findings.
2. What is the diagnosis?
3. What do the findings represent at the histologic/microscopic level?
4. What additional testing can be performed to help decide management?
5. What are the management options and what are the goals of treatment?

**Case 2** - A 72-year-old man with a history of hypertension and heart disease related to smoking is noted to have increased blood pressure on exam today. On interviewing the patient, you discover that he has discontinued taking his blood pressure medication. On further questioning, the embarrassed patient admits that he is unable to afford both the anti-hypertensive you have prescribed and the vitamin pills for which he pays his eye doctor $50/month. The patient has seen the terrible effect that macular degeneration has had on his friends and says that he’d rather be dead than lose his sight.

1. How would you advise this patient?
2. What data exists regarding the role of smoking and macular degeneration?

**Amblyopia and Strabismus Case Conference**

**Case 1** - A 3-year-old boy is brought in by his mother. Lately she has noticed that the child’s left eye turns in. The problem had been intermittent initially.


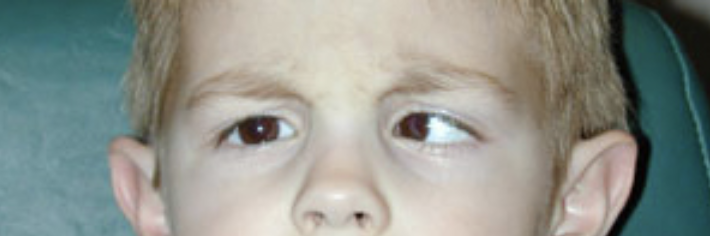


1. Describe the findings.
2. What historical information might be helpful?
3. What examination techniques would you use?

Image by University of Michigan Kellog Eye Center, retrieved from: <http://kellogg.umich.edu/theeyeshaveit/otherc/strabismus.html> on 5/29/2020. Creative Commons License associated: CC BY 3.0

1. What is the diagnosis?
2. What are the possible causes?
3. As the child’s primary care doctor how would you proceed from here?
4. Would you consult an eye doctor? If so, is this an emergent, urgent or routine consult?
5. Discuss possible treatments for this condition.

**Case 2** - You are invited to spend Thanksgiving at your roommate’s mother’s house. After the meal you retire to the living room and are treated to a slide show

of your roommate’s sister’s child. In several of the photographs you notice an odd appearance to the child’s eyes.


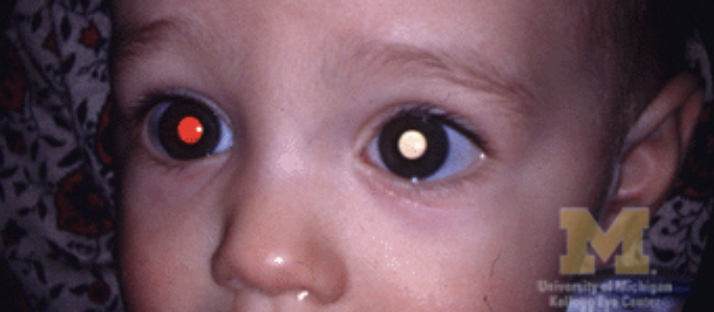


1. Describe and name the physical findings.

1. What are the possible causes?
2. How would you advise your roommate’s sister to proceed from here?

Image by University of Michigan Kellog Eye Center, retrieved from: <http://kellogg.umich.edu/theeyeshaveit/otherc/retinoblastoma.html> on 5/29/2020. Creative Commons License associated: CC BY 3.0

1. What examination techniques would you tell the family to expect?
2. After the consultation with the child’s doctor the distraught mother calls you to tell you that the diagnosis is a tumor in eye. Which tumor is most likely? Discuss the management options and prognosis.

**Case 3** - On call in the pediatric ER you are presented with a 6-month-old boy who was brought in by his mother. The child fell off the couch earlier in the day and has been difficult to arouse ever since. The left eye appears to be turned inward.

1. After assessment of the child’s vital signs how would you proceed?
2. What historical data would be important?
3. What, if any, techniques might be useful in examining the eyes?
4. Fundoscopic exam shows the findings below. Describe the findings.


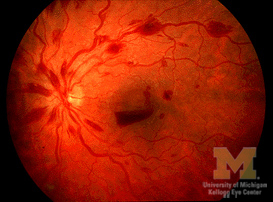


Image by University of Michigan Kellog Eye Center, retrieved from: <http://kellogg.umich.edu/theeyeshaveit/opticfundus/retinal_hemorrhages.html> on 4/14/2020. Creative Commons License associated: CC BY 3.0

1. What is the most likely diagnosis?
2. What additional tests should be considered?

**Acute Vision Loss Case Conference**

Please fill out the chart in Handout 5 titled *Acute Vision Loss - The Essentials* that corresponds to the below 9 photos. Include the diagnosis, presenting history, and common patient demographic associated with the condition, the timing of onset of the condition (minutes to hours, hours to days), if you would expect an afferent pupillary defect (APD), and the management and prognosis.

**Photo A.**


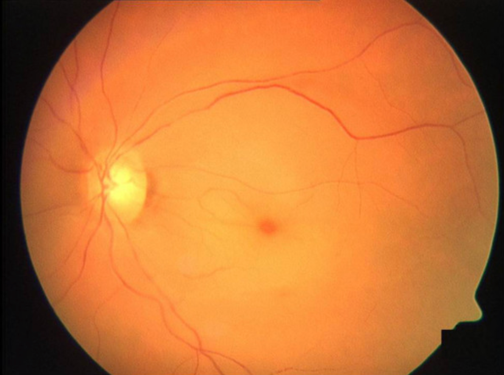


Image byAchim Fieß, Ömer Cal, Stephan Kehrein, Sven Halstenberg, Inez Frisch, Ulrich Helmut Steinhorst, retrieved from: <https://commons.wikimedia.org/w/index.php?curid=68585933>

on 5/29/2020. Creative Commons License associated: CC BY 2.0

**Photo B.**


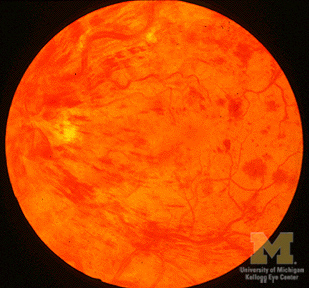


Image by University of Michigan Kellog Eye Center, retrieved from: <http://kellogg.umich.edu/theeyeshaveit/tehi_images/vein-occlusion.jpg> on 4/15/2020. Creative Commons License associated: CC BY 3.0

**Photo C.**


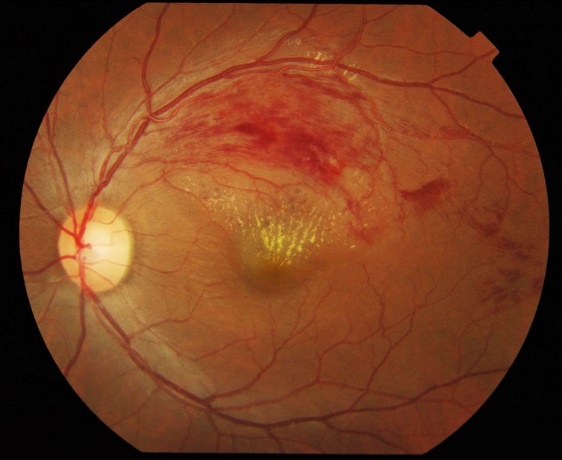


Image by Ku C Yong, Tan A Kah, Yeap T Ghee, Lim C Siang and Mae-Lynn C Bastion, Department of Ophthalmology, Universiti Kebangsaan Malaysia Medical Centre (UKMMC) and Universiti Malaysia Sarawak (UNIMAS), Kuala Lumpur, Malaysia, retrieved from: <http://www.biomedcentral.com/1471-2415/11/24/figure/F1> on 4/15/2020. Creative Commons License associated: CC BY 2.0

**Photo D.**


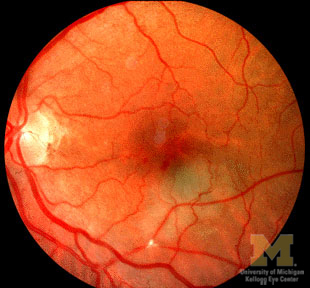


Image by University of Michigan Kellog Eye Center, retrieved from: <http://kellogg.umich.edu/theeyeshaveit/opticfundus/retinal_infarct.html> on 4/14/2020. Creative Commons License associated: CC BY 3.0

**Photo E.**


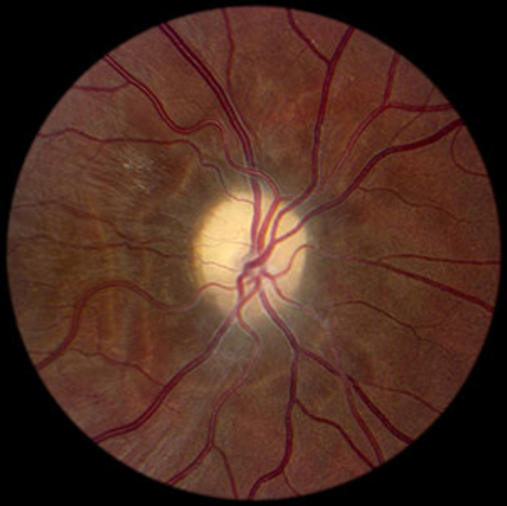


Image by University of Michigan Kellog Eye Center, retrieved from:

<http://kellogg.umich.edu/theeyeshaveit/opticfundus/disc_pallor.html> on 5/29/2020. Creative Commons License associated: CC BY 3.0

**Photo F.**


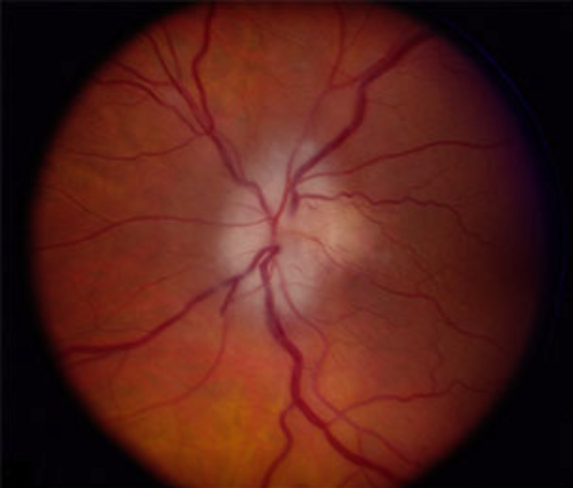


Image by University of Michigan Kellogg Eye Center, retrieved from: [http://kellogg.umich.edu/theeyeshaveit/system/giant_cell_arteritis.html on 4/25/2020](http://kellogg.umich.edu/theeyeshaveit/system/giant_cell_arteritis.html%20on%204/25/2020). Creative Commons License associated: CC BY 3.0

**Photo G.**


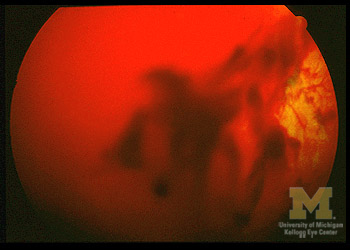


Image by University of Michigan Kellog Eye Center, retrieved from: http://kellogg.umich.edu/theeyeshaveit/tehi_images/vitreous-hemorrhage.jpg on 4/14/2020. Creative Commons License associated: CC BY 3.0

**Photo H.**


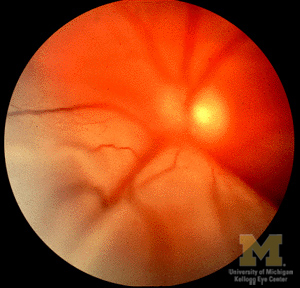


Image by University of Michigan Kellog Eye Center, retrieved from: <http://kellogg.umich.edu/theeyeshaveit/tehi_images/retinal-detachment.jpg> on 4/15/2020. Creative Commons License associated: CC BY 3.0

**Photo I.**


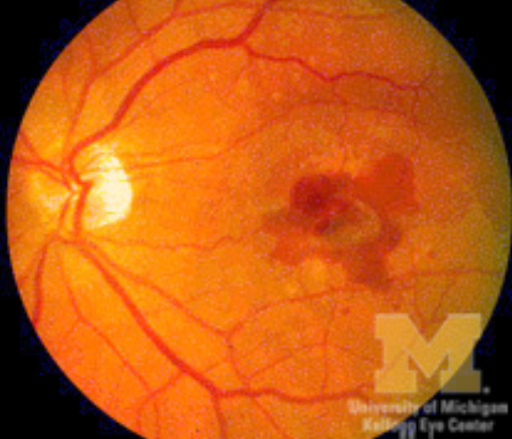


Image by University of Michigan Kellogg Eye Center, retrieved from: <http://kellogg.umich.edu/theeyeshaveit/opticfundus/subretinal_hemorrhage.html>

on 5/29/2020. Creative Commons License Associated: CC BY 3.0
