# Supplementary material for: A Required Ophthalmology Rotation: Providing Medical Students with a Foundation in Eye-Related Diagnoses and Management
Source: MedEdPORTAL. 2021 Feb 12;17:11100. doi: 10.15766/mep_2374-8265.11100 (PMC7880261; doi:10.15766/mep_2374-8265.11100)
Supplement: Supplementary file 1 — Ophthalmology Slides Instructors Guide.docxOphthalmology Handout.docxOphthalmology Slides.pptxOphthalmology Sessions.docxOphthalmology Sessions Answer Key.docxOphthalmology Sessions Student Handouts.docxOphthalmology Final Examination.docxStudent Postrotation Feedback Form.docx [file mep_2374-8265.11100-s001.zip › E. Ophthalmology Sessions Answer Key.docx]

**Cataract Case Conference – Answer Key**

**Case 1** - A 65-year-old man complains of difficulty seeing street signs while driving and also of glare from headlights that interferes with driving at night. On exam, his visual acuity with his current glasses is 20/50 OD (right) and 20/40 OS (left).


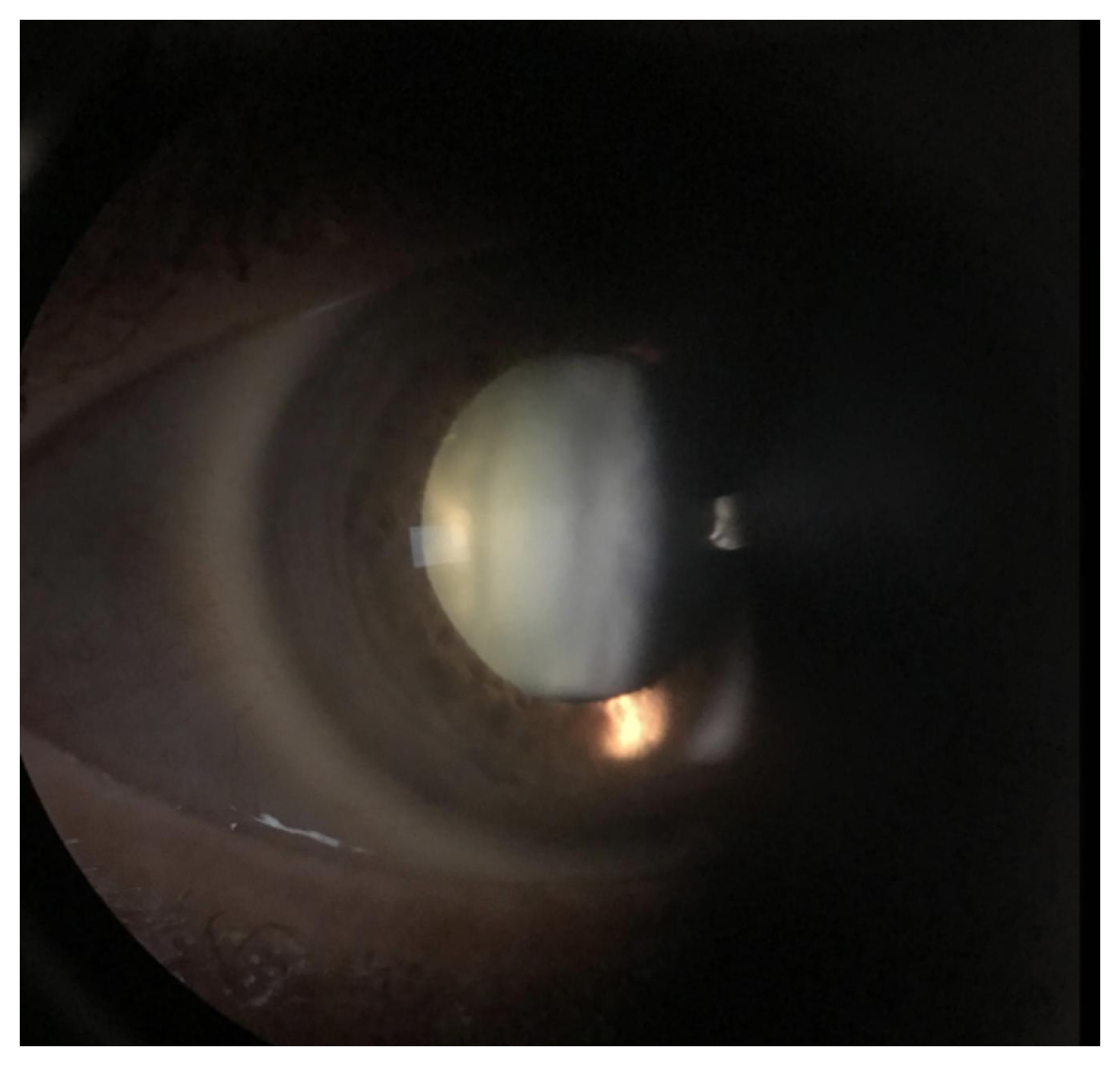


1. Describe the findings.

Nuclear sclerotic and cortical changes of the crystalline lens.

1. What is the diagnosis?

Combined age-related cataract.

1. What other elements of the eye exam are important in deciding on management?

Author Owned

Refraction (in an attempt to improve the vision with a new glasses prescription), dilated fundus examination (to look for other possible causes of decreased vision, which would not be helped by cataract surgery).

1. Further exam demonstrates that the vision is unimproved with a change in glasses prescription and that the remainder of the exam is normal. Discuss the medical and surgical management options. Be sure to compare the risks of further observation versus the risk of cataract surgery.

Observation vs. cataract surgery.

Risks of observation – vision will likely continue to worsen

Risks of cataract surgery – endophthalmitis (severe infection inside the eye, which could lead to loss of vision or even loss of the eye), blurred vision due to macular or corneal edema, ocular hypertension, diplopia, ptosis, need for spectacle correction at distance and/or near, posterior capsular opacity requiring a YAG laser capsulotomy in the clinic, need for additional surgery or procedure.

1. The patient asks for more detail on how cataract surgery is done and what he can expect during the surgery. In particular, he wants to know how the laser is used and how often he’ll need to have his implant replaced. Also, how long will the eye be removed from the socket?

We don’t typically use a laser. The implant generally lasts a lifetime (though sometimes they do have to be removed and replaced, due to dislocation caused by trauma or underlying ocular disease). Your eye stays right where it is during the surgery.

Cataract surgery is basically laparoscopic surgery—we make little tiny incisions in the front of the eye, then work inside the eye with little tiny instruments. A cataract is just the lens you were born with, that got cloudy as you got older. We break the cataract into 4 pieces, like cutting up a pie, and then we vacuum those pieces out. Then we take a shiny new artificial lens, roll it up in a tube like a burrito, and insert it through one of those little incisions into the eye. It sits behind the iris, the colored part of the eye, where the old lens was.

**Case 2** - A 72-year-old man is brought in by his wife for a second opinion regarding cataracts. He had recently seen another doctor who told them that he needed to have his cataracts removed as soon as possible. The patient does not drive and has no complaints about his vision. His current medical history is significant for Alzheimer’s disease, hypertension, and diabetes.

On exam, his visual acuity with his current glasses is 20/50 OD and 20/40 OS. Vision is unimproved with a change in glasses prescription. Anterior segment exam show 2+ nuclear sclerotic cataracts. Examination of the retina reveals some changes consistent with dry macular degeneration.

1. Discuss the medical and surgical management options. Make sure to include the indications for cataract surgery. When is cataract surgery “needed as soon as possible?”

Observation vs cataract surgery.

Cataract surgery is merited when the patient has decreased vision that interferes with his life. Since this patient does not drive and has no complaints about his vision, the risks of cataract surgery are likely to outweigh benefits. Additionally, his macular degeneration may also be affecting his vision, and he should have an OCT macula and potential acuity testing before proceeding to cataract surgery, even when he starts to complain of decreased vision.

Reasons for cataract surgery “as soon as possible”? Well…

Acute angle-closure with patent laser peripheral iridotomy comes to mind.

Poor vision that interferes with safe ambulation, too.

1. What is your advice to the patient and his wife?

Observation.

1. Would this advice be different if the patient’s vision was 20/400 in each eye and the wife noted that the patient was having trouble getting around the house?

Yes.

**Case 3** - You are the acting intern on the neo-natal unit. As you are examining a 1-day old baby girl you notice the finding below.


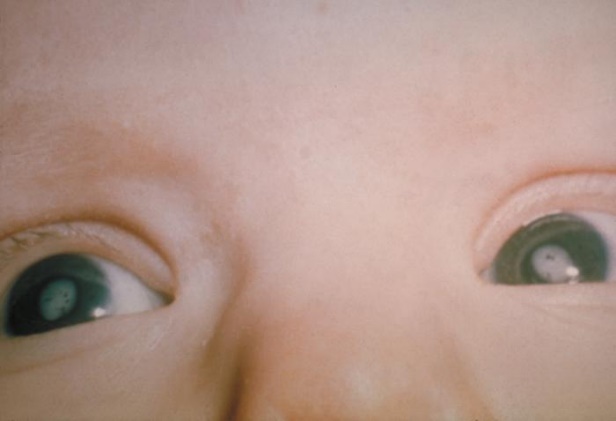


Image by Centers for Disease Control and Prevention, retrieved from: <https://phil.cdc.gov/phil_images/20030724/28/PHIL_4284_lores.jpg> on 4/12/2020. Creative Commons License associated: CC0 1.0

1. Describe the finding.

Bilateral leukocoria (white spot in the pupil).

1. What are the possible diagnoses? Which is most likely?

Congenital cataracts are most likely.

1. Assuming that this is bilateral cataracts, what are the management options? What are the indications for cataract surgery?

Cataract surgery should be performed at age 4-6 weeks for unilateral congenital cataract and by age 3 months for bilateral congenital cataract. If the cataracts are large enough to interfere with the patient’s vision, then surgery is merited to prevent amblyopia.

**Glaucoma Case Conference**

**Case 1** - A 46-year-old man comes to your office for a routine physical.

1. What elements of the pre-examination history would be relevant to his risk for glaucoma?

Family history of glaucoma (including diagnosis at young age, use of eye drops, and/or need for surgery). History of ocular trauma. History of steroid use, especially steroid eye drops.

1. Describe the parts of the eye examination that can be done in the primary care setting to further assess this patient’s glaucoma risk.

- Visual acuity
- Pupillary assessment including relative afferent pupillary defect (RAPD, or APD)
- Intraocular pressure using a Tonopen or iCare (if available) or by palpation through the patient’s closed upper eyelid
- Confrontation visual fields
- Direct ophthalmoscopy to examine the optic nerves

On history, the patient notes that he has no eye problems and that his glasses for myopia work well. He was hit in the right eye by a baseball when he was a teenager but after a few weeks he saw well and has had no problems since. His mother takes eye drops but he does not know the details.

On examination you note visual acuity of 20/20 with glasses in both eyes. On checking the pupils you notice a small amount of redilation of the pupils of both eyes when the light is moved from the left eye to the right eye and a small amount of constriction of both pupils when the light is moved from the right eye to the left eye. Exam of the right eye with the direct ophthalmoscope is shown below.


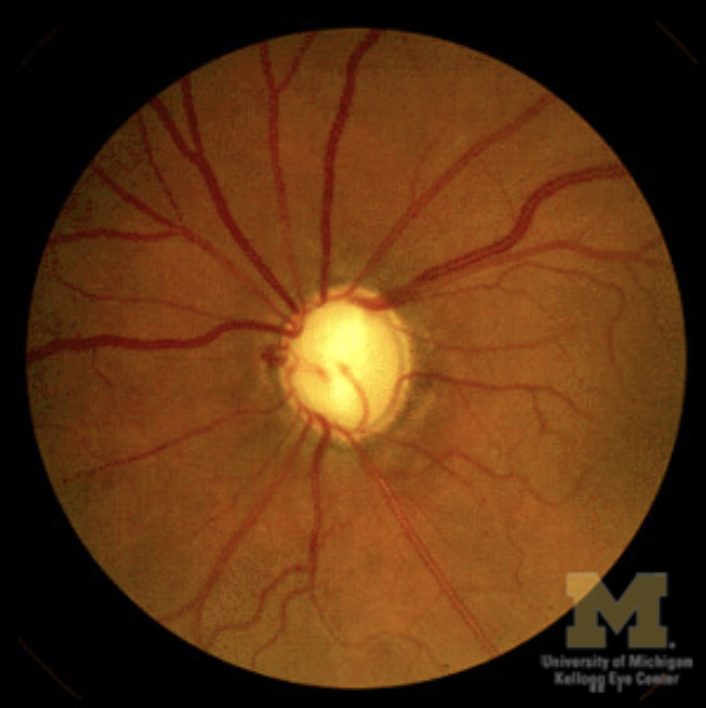


Image by University of Michigan Kellogg Eye Center, retrieved from: <http://kellogg.umich.edu/theeyeshaveit/opticfundus/disc_cupping.html>

on 5/29/2020. Creative Commons License Associated: CC BY 3.0

1. Describe the findings.

Extensive cupping of the optic disc, with thin rim of healthy nerve tissue and large pale center, displacement of the retinal vasculature to the periphery of the nerve.

1. What are the likely diagnoses?

Glaucoma (while pallor may be seen with many causes of optic neuropathy, cupping is pretty specific for glaucoma).

1. Describe further management in the primary care setting. Is this an emergent, urgent or routine problem?

Avoid nocturnal hypotension (if he needs blood pressure medications, try to give these in the morning). This is a routine problem. He does need to see an ophthalmologist, preferably a glaucoma specialist. He does need regular follow up and likely treatment for his glaucoma to prevent permanent vision loss. However, optic disc pallor takes 6-8 weeks to develop, and the baseball hit his eye decades ago. This is a chronic problem.

1. Describe management by the ophthalmic consultant.

Regular follow up (likely every 3-4 months, given the advanced cupping) with intraocular pressure measurements at every visit, along with visual field testing and OCT imaging of the retinal nerve fiber and ganglion cell layers at intervals.

First-line treatments include Selective Laser Trabeculoplasty (SLT) laser treatment in clinic vs. regular eye drop use at home (prostaglandin analogues, beta-blockers, alpha-agonists, carbonic anhydrase inhibitors).

Numerous surgical options are available, including trabeculectomy, seton (tube shunt) devices, and newer so-called “minimally invasive” options.

**Case 2** - A 59-year-old woman comes to the emergency dept


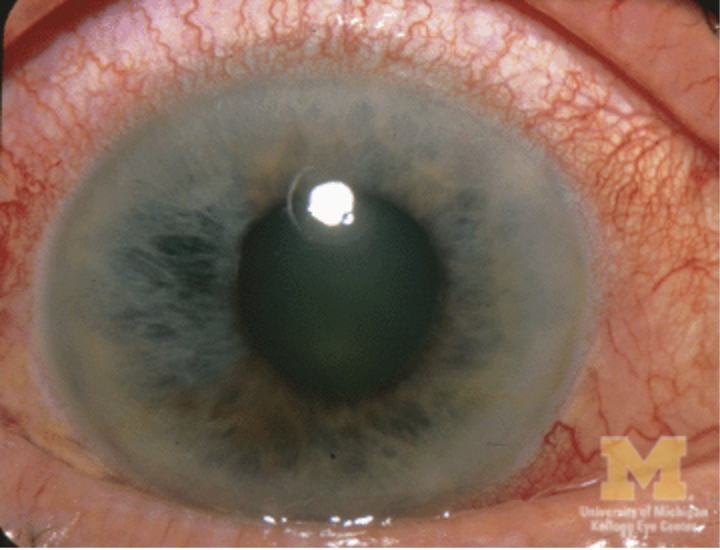


complaining of severe right eye pain beginning this morning.

The pain has been getting worse through the day and the vision

in that eye has been getting progressively foggy and dim.

She has no previous eye history except for wearing glasses.

She’s had a cold recently for which she’s been taking an over

the counter cold remedy.

Image by University of Michigan Kellogg Eye Center, retrieved from: <http://kellogg.umich.edu/theeyeshaveit/redeye/angleclosure_glaucoma.html> on 5/29/2020. Creative Commons License Associated: CC BY 3.0

1. What additional historical details might be helpful?

Prior episodes of ocular pain, redness, and/or halos around

lights, particularly after a dilated eye exam or in dim lighting conditions.

1. Describe the parts of the eye examination that can be done in the ED setting to further assess this patient’s problem.

- Visual acuity
- Pupillary assessment including looking for a fixed, mid-dilated pupil and checking for a relative afferent pupillary defect (RAPD, or APD)
- Intraocular pressure using a Tonopen or iCare (if available) or by palpation through the patient’s closed upper eyelid
- Penlight exam of the anterior segment, including shining the light from the side to assess the anterior chamber depth and looking for corneal clouding (due to edema)
- Direct ophthalmoscopy to examine the optic nerves

On review of additional history, the patient tells you that she has needed her glasses for reading more than for distance. The cold medicine she was taking has pseudophedrine in it. She remembers driving her aunt to an appointment for laser surgery a long time ago.

On examination, the vision is 20/400 on the right and 20/20 on the left. While the pupil response on the right is normal, the left pupil appears fixed and mid-dilated. The right eye appears normal to a penlight exam but the cornea on the left is cloudy looking. Gentle palpation of the eyes shows that the left eye is much harder than the right. Direct ophthalmoscopy is unrewarding.

1. What is the likely diagnosis?

Acute angle-closure.

1. What steps can be taken in the ED setting to manage this patient? Is this an emergent, urgent or routine problem?

Topical aqueous suppressants (e.g., beta-blockers, alpha-agonists, carbonic anhydrase inhibitors)

Oral or intravenous acetazolamide (Diamox) or methazolamide.

Oral glycerin or intravenous mannitol (though be careful in the elderly, as rapid intracranial fluid shifts can tear bridging veins).

This is an emergent problem.

1. Describe management by the ophthalmic consultant.

The ophthalmic consultant will do many of the same things that the emergency department physician can do:

Give topical aqueous suppressants (e.g., beta-blockers, alpha-agonists, carbonic anhydrase inhibitors) – 2 or 3 rounds of drops given 5-10 minutes apart.

Administer oral or intravenous acetazolamide (Diamox) or methazolamide.

Perform anterior chamber paracentesis (basically sticking a needle into the anterior chamber to release aqueous fluid and acutely bring down the intraocular pressure) – this doesn’t last long, maybe a couple hours, though it does help the corneal edema, making the eye drops penetrate better and improving the view for a more definitive laser treatment.

And lastly, the consultant can more definitive treatment with laser peripheral iridotomy.

**Case 3** – A 2-year-old boy is brought to the pediatrician. The mother notes that the left eye doesn’t look right and tears a lot.


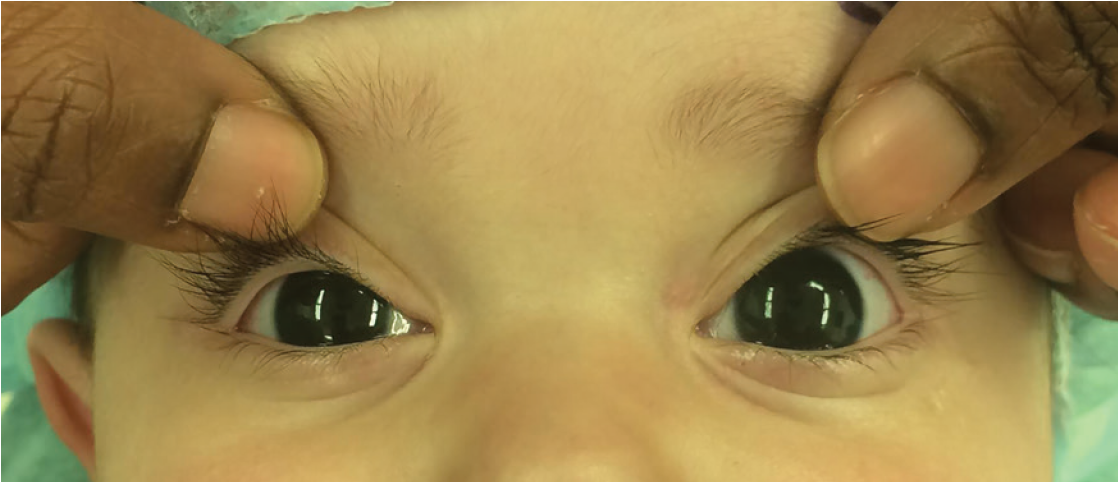


1. What diagnosis needs to be considered?

Congenital glaucoma.

1. Describe further management for this condition in the primary care setting. Is this an emergent, urgent or routine problem?

Image by American Academy of Ophthalmology, used with permission

Urgent, maybe emergent.

1. Describe management by an ophthalmologist in present day.

Topical medications to lower the eye pressure including prostaglandin analogues, beta-blockers, and carbonic anhydrase inhibitors. Be careful with alpha-agonists – brimonidine (Alphagan) causes sedation and apnea in little ones and thus should not be used in young children (<8 years old); apraclonidine (Iopidine) is safer in this age group.

Surgical options are more definitive and include trabeculectomy surgery or angle-based procedures such as goniotomy or trabeculotomy (basically cutting through the trabecular meshwork to physically open the anatomically abnormal drainage angle). These angle-based procedures work particularly well in pediatric patients.

**Macular Degeneration Case Conference**

**Case 1** - A 72-year-old woman has noticed distortion of her vision over the last month. Everything appears normal when only the right eye is opened. When the left eye is open straight lines appear to be bent in the middle.

The retinal exams of the left and right eyes are shown below.


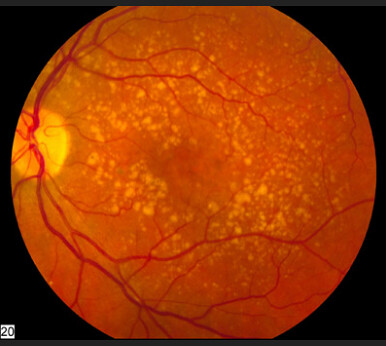


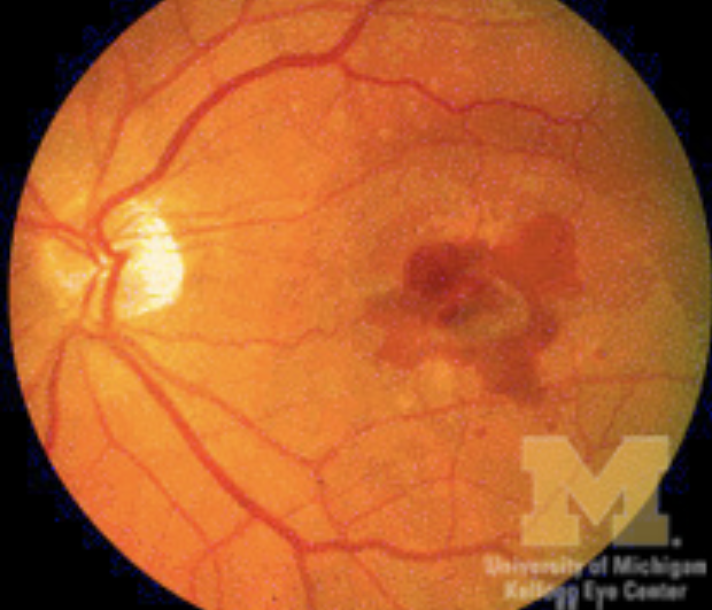


Image by National Eye Institute, retrieved from: <https://search.creativecommons.org/photos/5b566129-05d3-4204-997b-2ef9f6c14379> on 4/13/2020. Creative Commons License associated:  [CC BY 2.0](https://creativecommons.org/licenses/by-nc-nd/2.0/?ref=ccsearch&atype=rich)

Image by University of Michigan Kellogg Eye Center, retrieved from: <http://kellogg.umich.edu/theeyeshaveit/opticfundus/subretinal_hemorrhage.html>

on 5/29/2020. Creative Commons License Associated: CC BY 3.0

1. Describe the findings.

Right eye – large subretinal hemorrhage in the central macula (presumably due to choroidal neovascular membrane) with a couple smaller subretinal hemorrhages in the superior macula, patchy macular atrophy and drusen.

Left eye – extensive variably sized hard and soft drusen throughout the macula, central pigmentary changes.

1. What is the diagnosis?

Age-related macular degeneration (AMD).

1. What do the findings represent at the histologic/microscopic level?

Drusen represent lipid and protein that originated from the choroidal circulation and have deposited under the retinal pigment epithelium, atrophy represents degeneration of the photoreceptors and supporting retinal pigment epithelial cells.

Subretinal hemorrhage and/or subretinal grey-green appearance in the central macula is due to a choroidal neovascular membrane, which represents abnormal blood vessel growth under the macula.

1. What additional testing can be performed to help decide management?

Amsler grid to look for metamorpsia and/or scotoma, which could suggest conversion to wet AMD.

OCT macula to look for fluid (blood) within or under the retina in either eye; given that the exam shows subretinal hemorrhage in the right eye, this eye will likely need an intravitreal injection of an anti-VEGF agent. If OCT macula of the left eye shows fluid, then it may need an injection, too. This is what we call “wet” AMD (vs “dry” AMD with drusen, pigmentary change, and atrophy).

1. What are the management options and what are the goals of treatment?

See answer to #4. For dry AMD, we recommend smoking cessation and “eye vitamins” with the AREDS2 formulation (AREDS2 or AREDS are acceptable, if the patient is a non-smoker). For wet AMD, we treat with intravitreal injections of anti-VEGF agents, e.g., bevacizumab (Avastin) or aflibercept (Eylea).

**Case 2** - A 72-year-old man with a history of hypertension and heart disease related to smoking is noted to have increased blood pressure on exam today. On interviewing the patient, you discover that he has discontinued taking his blood pressure medication. On further questioning, the embarrassed patient admits that he is unable to afford both the anti-hypertensive you have prescribed and the vitamin pills for which he pays his eye doctor $50/month. The patient has seen the terrible effect that macular degeneration has had on his friends and says that he’d rather be dead than lose his sight.

1. How would you advise this patient?

Smoking cessation, resume his blood pressure medication, eat dark leafy greens and oily fish.

1. What data exists regarding the role of smoking and macular degeneration?

Evidence that smoking cessation helps prevent progression of AMD is much stronger than that for nutrition and/or nutritional supplements. Smoking is the strongest modifiable risk factor for AMD, and multiple studies have demonstrated at least a 2-fold decrease in risk of progression of AMD with smoking cessation. Interestingly, hypertension also shows up on list of modifiable risk factors (although it appears to be a very weak one compared to smoking). Not treating his hypertension puts this patient at risk for cerebrovascular accident (CVA) and myocardial infarction (MI), an association which also has much better supporting evidence that evidence surrounding nutrition and AMD. AREDS and AREDS2 studies that showed a benefit for nutritional supplementation for AMD. Other studies have had negative findings.

**Amblyopia and Strabismus Case Conference**

**Case 1** - A 3-year-old boy is brought in by his mother. Lately she has noticed that the child’s left eye turns in. The problem had been intermittent initially.


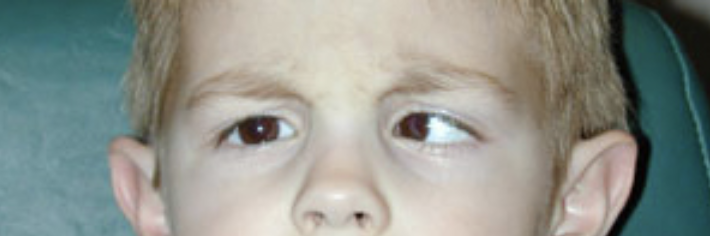


1. Describe the findings.

Left esotropia (left eye turning inward).

1. What historical information might be helpful?

Image by University of Michigan Kellog Eye Center, retrieved from: <http://kellogg.umich.edu/theeyeshaveit/otherc/strabismus.html> on 5/29/2020. Creative Commons License associated: CC BY 3.0

The usual (onset, timing, duration, exacerbating/relieving

factors), plus recent trauma and history of patching,

eyeglasses, or surgery. Also, history of sickle cell

(these children are at increased risk of strokes).

1. What examination techniques would you use?

Cover-uncover test, alternating cover test, and prism cover testing (measure the deviation using prisms, while performing alternating cover test). Also, stereopsis testing (if this 2-year-old patient is able to cooperate).

1. What is the diagnosis?

Left esotropia (previously left intermittent esotropia).

1. What are the possible causes?

Congenital esotropia. Accommodative esotropia due to uncorrected hyperopic refractive error. Less likely cranial nerve (CN) VI palsy (from brain bleed secondary to trauma or, in a child with sickle cell, stroke is a possible cause).

1. As the child’s primary care doctor how would you proceed from here?

Referral to pediatric ophthalmologist.

1. Would you consult an eye doctor? If so, is this an emergent, urgent or routine consult?

Yes. Regarding urgency, this depends on history. If this is long-standing, likely a routine consult. Worsening recently (as in this case) may warrant a more urgent clinic visit. Sudden onset would make this concerning for CN VI palsy, which in a child would be highly unusual and merit an emergency department visit.

1. Discuss possible treatments for this condition.

Eye muscle (strabismus) surgery, patching (to treat/prevent amblyopia).

**Case 2** - You are invited to spend Thanksgiving at your roommate’s mother’s house. After the meal you retire to the living room and are treated to a slide show

of your roommate’s sister’s child. In several of the photographs you notice an odd appearance to the child’s eyes.


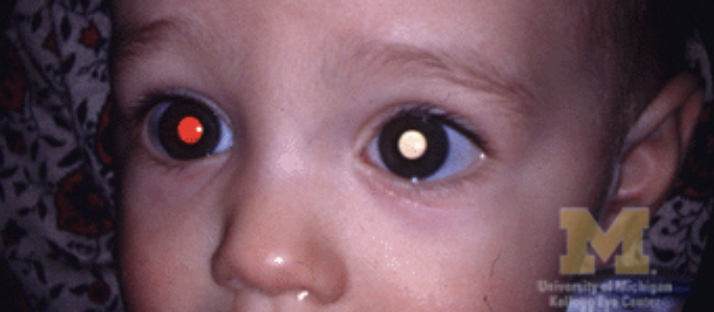


1. Describe and name the physical findings.

Leukocoria (white pupil reflex).

1. What are the possible causes?

Image by University of Michigan Kellog Eye Center, retrieved from: <http://kellogg.umich.edu/theeyeshaveit/otherc/retinoblastoma.html> on 5/29/2020. Creative Commons License associated: CC BY 3.0

Retinoblastoma, congenital (or traumatic) cataract,
ROP (retinopathy of prematurity), PPHV (persistent
hyperplastic primary vitreous).

1. How would you advise your roommate’s sister to proceed from here?

Schedule an urgent appointment with a pediatric ophthalmologist.

1. What examination techniques would you tell the family to expect?

Dilating eye drops, dilated fundus examination, slit lamp examination (via flying baby technique). An examination under anesthesia may be needed, if the patient is not able to cooperate in clinic.

1. After the consultation with the child’s doctor the distraught mother calls you to tell you that the diagnosis is a tumor in eye. Which tumor is most likely? Discuss the management options and prognosis.

Retinoblastoma (RB). Enucleation (removal of the eye) vs. brachytherapy (radioactive iodine plaque). MRI is needed to look for mid-line tumor, including pinealoma. Regularly scheduled examination under anesthesia of the fellow eye, in order to dilate and look for RB.

**Case 3** - On call in the pediatric ER you are presented with a 6-month-old boy who was brought in by his mother. The child fell off the couch earlier in the day and has been difficult to arouse ever since. The left eye appears to be turned inward.

1. After assessment of the child’s vital signs how would you proceed?

Contact the state’s designated agency for child protective services immediately. Notify your attending physician, as well.

1. What historical data would be important?

Prior visits with traumatic injuries.

1. What, if any, techniques might be useful in examining the eyes?

Dilated fundus examination via direct ophthalmoscopy, indirect ophthalmoscopy, and/or slit lamp biomicroscopy. Retinal photos with a portable fundus camera.

1. Fundoscopic exam shows the findings below. Describe the findings.


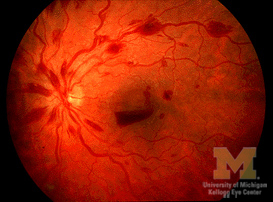


Image by University of Michigan Kellog Eye Center, retrieved from: <http://kellogg.umich.edu/theeyeshaveit/opticfundus/retinal_hemorrhages.html> on 4/14/2020. Creative Commons License associated: CC BY 3.0

1. What is the most likely diagnosis?

Non-accidental trauma (a.k.a. “shaken baby syndrome”).

1. What additional tests should be considered?

CT of the head looking for intracranial hemorrhages (especially subdural hemorrhages) and head-to-toe skeletal survey looking for evidence of fractures (possibly at various stages of healing, reflecting multiple injuries inflicted over time).

**Acute Vision Loss Case Conference**

Please fill out the chart in Handout 5 titled *Acute Vision Loss - The Essentials* that corresponds to the below 9 photos. Include the diagnosis, presenting history, and common patient demographic associated with the condition, the timing of onset of the condition (minutes to hours, hours to days), if you would expect an afferent pupillary defect (APD), and the management and prognosis.

**Photo A.**


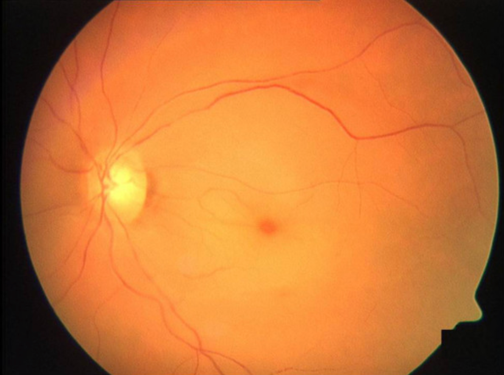


Image byAchim Fieß, Ömer Cal, Stephan Kehrein, Sven Halstenberg, Inez Frisch, Ulrich Helmut Steinhorst, retrieved from: <https://commons.wikimedia.org/w/index.php?curid=68585933>

on 5/29/2020. Creative Commons License associated: CC BY 2.0

**Photo B.**


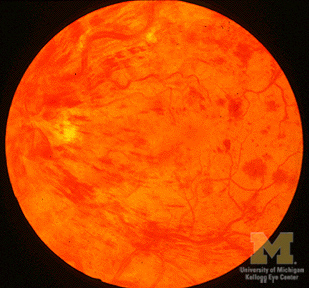


Image by University of Michigan Kellog Eye Center, retrieved from: <http://kellogg.umich.edu/theeyeshaveit/tehi_images/vein-occlusion.jpg> on 4/15/2020. Creative Commons License associated: CC BY 3.0

**Photo C.**


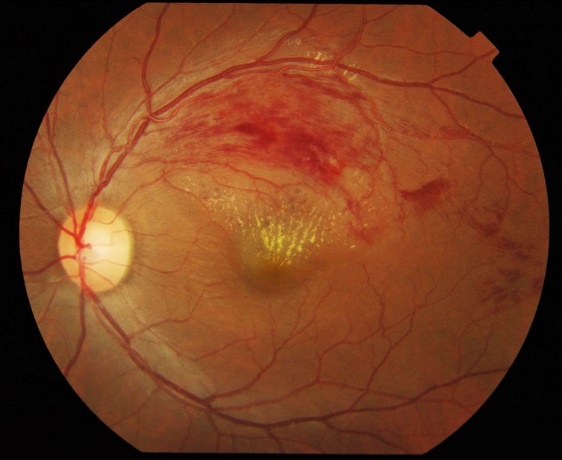


Image by Ku C Yong, Tan A Kah, Yeap T Ghee, Lim C Siang and Mae-Lynn C Bastion, Department of Ophthalmology, Universiti Kebangsaan Malaysia Medical Centre (UKMMC) and Universiti Malaysia Sarawak (UNIMAS), Kuala Lumpur, Malaysia, retrieved from: <http://www.biomedcentral.com/1471-2415/11/24/figure/F1> on 4/15/2020. Creative Commons License associated: CC BY 2.0

**Photo D.**


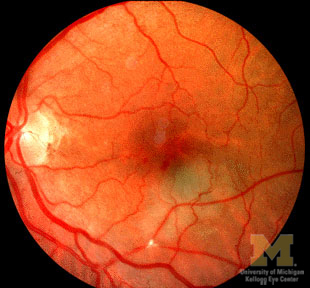


Image by University of Michigan Kellog Eye Center, retrieved from: <http://kellogg.umich.edu/theeyeshaveit/opticfundus/retinal_infarct.html> on 4/14/2020. Creative Commons License associated: CC BY 3.0

**Photo E.**


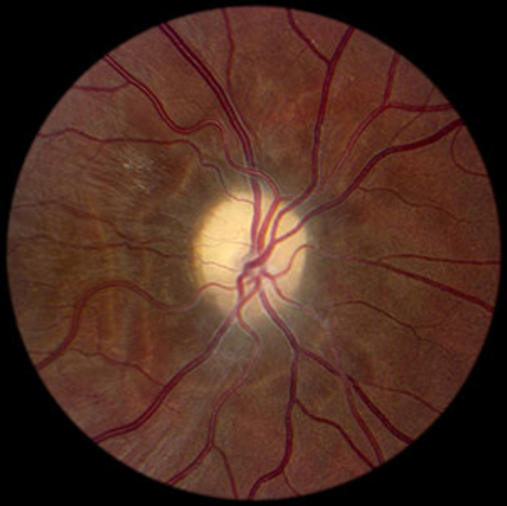


Image by University of Michigan Kellog Eye Center, retrieved from:

<http://kellogg.umich.edu/theeyeshaveit/opticfundus/disc_pallor.html> on 5/29/2020. Creative Commons License associated: CC BY 3.0

**Photo F.**


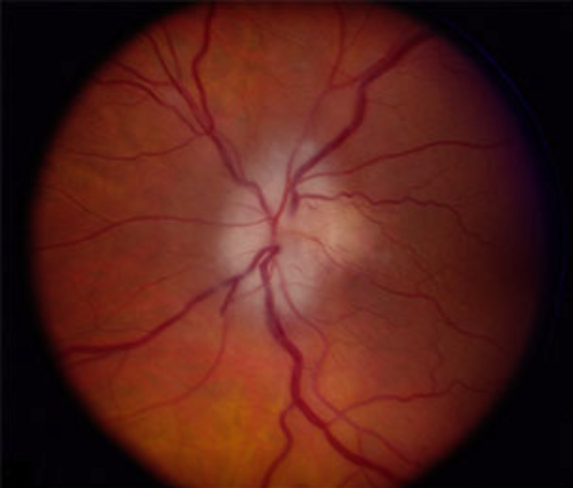


Image by University of Michigan Kellogg Eye Center, retrieved from: [http://kellogg.umich.edu/theeyeshaveit/system/giant_cell_arteritis.html on 4/25/2020](http://kellogg.umich.edu/theeyeshaveit/system/giant_cell_arteritis.html%20on%204/25/2020). Creative Commons License associated: CC BY 3.0

**Photo G.**


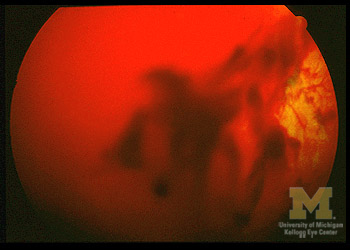


Image by University of Michigan Kellog Eye Center, retrieved from: http://kellogg.umich.edu/theeyeshaveit/tehi_images/vitreous-hemorrhage.jpg on 4/14/2020. Creative Commons License associated: CC BY 3.0

**Photo H.**


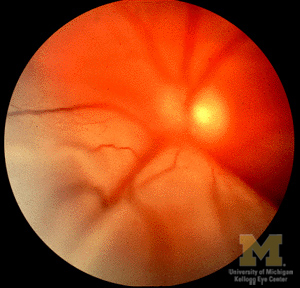


Image by University of Michigan Kellog Eye Center, retrieved from: <http://kellogg.umich.edu/theeyeshaveit/tehi_images/retinal-detachment.jpg> on 4/15/2020. Creative Commons License associated: CC BY 3.0

**Photo I.**


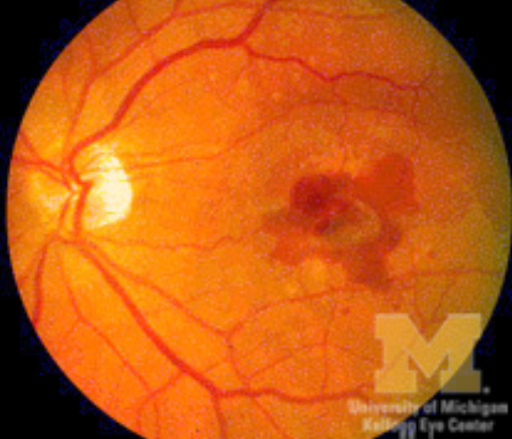


Image by University of Michigan Kellogg Eye Center, retrieved from: <http://kellogg.umich.edu/theeyeshaveit/opticfundus/subretinal_hemorrhage.html>

on 5/29/2020. Creative Commons License Associated: CC BY 3.0

**Acute Vision Loss Case Conference Photo Answer Key**

1. Central Retinal Artery Occlusion (CRAO)
2. Central Retinal Vein Occlusion (CRVO)
3. Branch Retinal Vein Occlusion (BRVO)
4. Branch Retinal Artery Occlusion (BRAO)
5. Optic Nerve Pallor due to previous Optic Neuritis
6. Arteritic Anterior Ischemic Optic Neuropathy (AAION)/Giant Cell Arteritis (GCA)
7. Vitreous Hemorrhage (VH)
8. Retinal Detachment (RD)
9. Wet Age-Related Macular Degeneration (wet AMD)

| **Photo** | **Diagnosis** | **History/**  **Demographic** | **Timing** | **APD?** | **Red Reflex?** | **Management** | **Prognosis** |
| --- | --- | --- | --- | --- | --- | --- | --- |
| **A** | Central Retinal Artery Occlusion (CRAO) | Sudden loss of vision. Older patient with vascular risk factors, carotid stenosis, and/or arrthymia such  as Afib. Can be due to giant cell arteritis (GCA); see answer for photo F below for expected history. | Seconds to minutes | Yes, marked | Yes | Immediate ESR, CRP, and platelet count to rule out GCA, if at least 55 years old and no embolus on exam. Stroke workup (BP, fasting glucose, A1c, lipids, CBC with differential, PT/PTT, MRI brain, imaging of carotid arteries [duplex Doppler US, CTA or MRA head/neck], EKG, echo). Consider tPA directly into ophthalmic artery (if at specialized center and patient presents within 3 hr). May try ocular massage and/or IOP lowering with acetazolamide 500 mg IV or PO, based on anecdotal evidence. | Poor prognosis with very limited potential for visual recovery. Increased risk of stroke, so important to treat any risk factors. Neo-vascularization may develop, requiring laser treatment &/or anti-VEGF injections. |
| **B** | Central Retinal Vein Occlusion (CRVO) | Sudden loss of vision in patient  with vascular  risk factors. Caused by atherosclerosis  of adjacent central retinal artery, compressing vein with secondary thrombosis. | Minutes to hours | Yes or No | Yes | Assess vascular risk factors by obtaining BP, fasting glucose and A1c, lipids, CBC with differential and platelets, PT/PTT, ESR.  In younger patients, consider assessing for hypercoagulable state.  Treat underlying conditions. If possible, discontinue oral contraceptives and diuretics. Follow up with internist for cardiovascular evaluation. | Vision may improve. 8-10% develop CRVO in other eye.  Macular edema and/or neo-vascularization often develop, requiring laser treatment &/or anti-VEGF injections. |
| **C** | Branch Retinal Vein Occlusion (BRVO) | Sudden partial loss of visual field | Minutes to hours | No | Yes | Assess vascular risk factors by obtaining BP, fasting glucose and A1c, lipids, CBC with differential and platelets, PT/PTT, ESR.  In younger patients, consider assessing for hypercoagulable state.  Treat underlying conditions. Follow up with internist for cardiovascular evaluation. | Vision may improve. Macular edema and/or neo-vascularization may develop, requiring laser treatment &/or anti-VEGF injections. |
| **D** | Branch Retinal Artery Occlusion (BRAO) | Sudden partial loss of visual field, possibly with recent amaurosis (transient vision loss). | Seconds to minutes | Yes or No | Yes | Immediate ESR, CRP, and platelet count to rule out GCA, if at least 55 years old and no embolus on exam. Stroke workup (BP, fasting glucose, A1c, lipids, CBC with differential, PT/PTT, MRI brain, imaging of carotid arteries [duplex Doppler US, CTA or MRA head/neck], EKG, echo). May try ocular massage and/or IOP lowering with acetazolamide 500 mg IV or PO, based on anecdotal evidence. | Poor prognosis with limited potential for visual recovery. Increased risk of stroke, so important to treat any risk factors. Neo-vascularization is rare. |
| **E** | Optic Nerve Pallor due to previous Optic Neuritis | Young adults, orbital pain especially with eye movements, decreased perception of colors and light intensity. | Hours to days for optic neutitis, weeks to months for pallor | Yes | Yes | MRI brain/orbits with gadolinium and fat suppression, consider IV methylprednisolone 1 g/day x 3 days followed by PO prednisone 1 mg/kg/day x 11 days (do NOT treat with PO steroids only). | Vision typically improves; IV steroids speed the process. If demyelinating lesions on MRI, 72% develop MS over 15 years (vs. 25% if no WM lesions). |
| **F** | Arteritic Anterior Ischemic Optic Neuropathy (AAION)/ Giant Cell Arteritis (GCA) | Sudden vision loss in older adults (at least 55 years of age). Many have HA, jaw claudication (pain with chewing), scalp tenderness especially over temple, fever, decreased weight and/or appetite. | Hours to days | Yes | Yes | Immediate ESR, CRP, and platelet count. Start systemic steroids immediately and obtain temporal artery biopsy within 1 week. Start with IV methylprednisolone 250 mg q6h x 3 days, then switch to PO prednisone 80-100 mg daily with a taper to lowest necessary dose and total treatment length of 6-12 months. Prophylactic antacid and osteoporosis prevention are needed. | Rapid progression from amaurosis (episodes of transient visual loss) to permanent vision loss. If not treated quickly, other eye can lose vision within  a week. |
| **G** | Vitreous Hemorrhage (VH) | Sudden onset with dark spots and/or cloudy haze in vision. May result from proliferative retinopathy (from diabetes, vein occlusion, or sickle cell), trauma, tumor, posterior vitreous detachment (PVD) or retinal tear. | Minutes to hours | No to Mild | Absent if severe | Ask about history of DM, HTN, sickle cell, trauma, malignancy, recent flashes/floaters, diabetic retinopathy, retinal vein occlusion. Look for neovascularization of iris, optic disc, and/or retina. Perform B scan ultrasonography to look for RD or tumor, if no view. Treat underlying cause.  Precautions include head of bed elevated; no bending, straining, or lifting; no blood thinners (unless medically necessary). | Depends on the cause. |
| **H** | Retinal Detachment (RD) | Any age, usually older. Flashes, floaters, curtain or shadow moving to cover field of vision, usually starts peripherally and moves centrally. | Minutes to hours | No to Mild | Yes | Urgent surgical repair with vitrectomy and gas  bubble, or with scleral buckle. Laser around any retinal tears. | Depends on extent of detachment; better if macula-on & repaired within 48 hours |
| **I** | Wet Age-Related Macular Degeneration (wet AMD) | Most common in elderly Caucasians. Drusen, subretinal fluid with fluid between the retina and RPE and/or focal RPE detachment(s) with fluid beneath the RPE. | Hours to days | Yes or No | Yes | For dry AMD: smoking cessation; AREDS2 eye vitamins for bilateral intermediate or unilateral severe dry AMD.  For wet AMD: intravitreal injections of anti-VEGF agents including bevacizumab (Avastin), ranibizumab (Lucentis), or aflibercept (Eylea). | May do well with frequent (up to monthly) injections. Permanent vision loss  if central scarring (subretinal fibrosis) develops. |
