# Supplementary material for: A Required Ophthalmology Rotation: Providing Medical Students with a Foundation in Eye-Related Diagnoses and Management
Source: MedEdPORTAL. 2021 Feb 12;17:11100. doi: 10.15766/mep_2374-8265.11100 (PMC7880261; doi:10.15766/mep_2374-8265.11100)
Supplement: Supplementary file 1 — Ophthalmology Slides Instructors Guide.docxOphthalmology Handout.docxOphthalmology Slides.pptxOphthalmology Sessions.docxOphthalmology Sessions Answer Key.docxOphthalmology Sessions Student Handouts.docxOphthalmology Final Examination.docxStudent Postrotation Feedback Form.docx [file mep_2374-8265.11100-s001.zip › F. Ophthalmology Sessions Student Handouts.docx]

**Handout 1: Cataract - the Essentials**


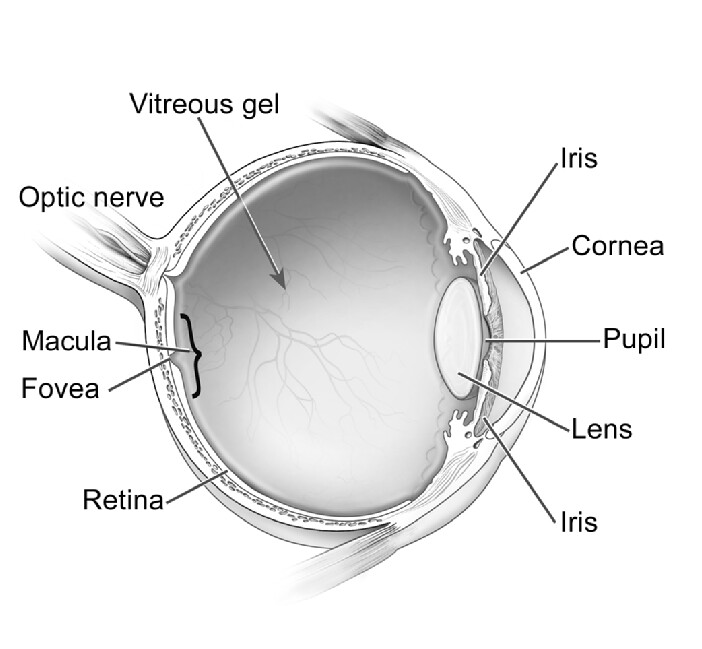


**Definitions**

*Cataract* - opacity of the crystalline lens *Visually Significant Cataract* - a cataract that causes a noticeable decrease in vision

*Functionally Significant Cataract* - a visually significant cataract that interferes with a person’s ability to perform necessary or desired tasks

For many people a cataract become visually and functionally significant when vision is decreased to 20/40 or worse.

Image by National Eye Institute, retrieved from: <https://search.creativecommons.org/photos/fe9ce6ae-08e5-4988-bbb3-9f1dcef01fef>

on 4/17/2020. Creative Commons License associated: CC BY 2.0

**Epidemiology**

Most common cause of decreased vision not correctable with glasses. Incidence - 50% people between 65 and 74, 70% in people 75 and over.


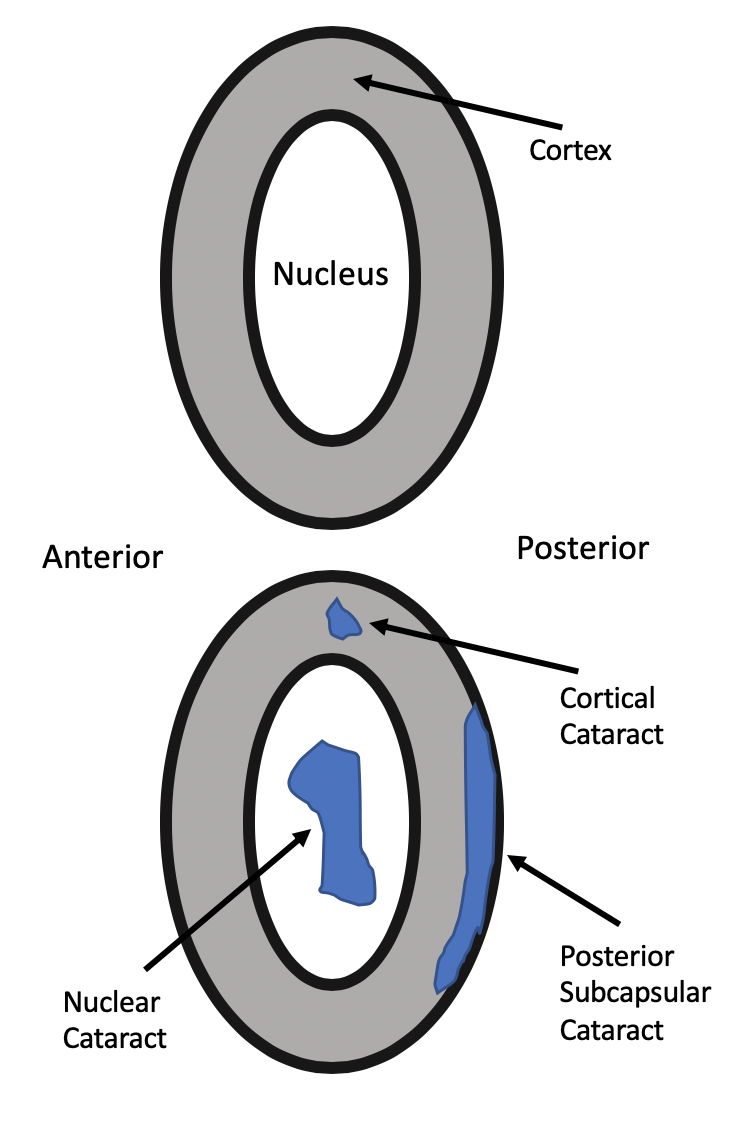


**Etiology**

Most common - age related changes in lens protein content (*senile cataract*)

Other - trauma, inflammation, metabolic defects (e.g.

diabetes, steroid use), radiation, congenital

Cataract formation is accelerated by smoking and sun

exposure.


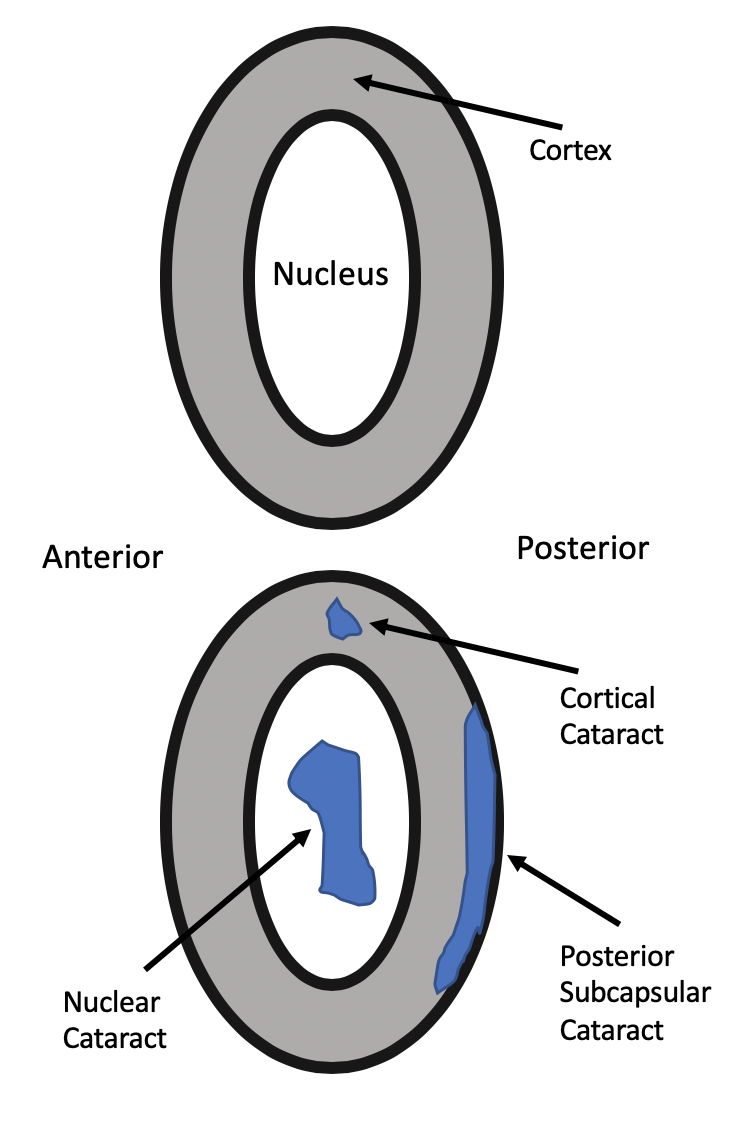


**Symptoms**

Most common - change in refractive error, image blur, glare

Other - monocular diplopia, image distortion, altered color perception

**Signs**

Decreased *red reflex* with direct ophthalmoscope. When very advanced may appear as a white pupil or *leukocoria.*

At slit lamp cataracts can be seen to be of three major types.

1. *nuclear* - yellowing/clouding of central lens

Figure Author Owned

1. *cortical* - opacification of cortex the intermediate layer of the lens
2. *posterior subcapsular* - opacification of the cortex immediately adjacent to the posterior lens capsule

Each person’s lens may have one or all of these types of opacities.

**Treatment**

*Senile cataract*

There is no medical cure for cataract. When the cataract is visually and functionally significant the patient is offered the option of cataract surgery. Cataract surgery is almost always elective surgery as the cataract does not damage the eye. Rarely a cataract may become so advanced that it may cause glaucoma and iritis. No patient need be considered to old for cataract surgery. Cataract surgery is usually outpatient surgery done under local anesthesia with monitoring. Success rates for surgery exceed 95%.

Cataract extraction involves removal of the nucleus and cortex of the lens. The capsule is left behind to support a synthetic lens implant. *Phacoemulsification* surgery involves removal of lens material through a small incision after breaking it into pieces using ultrasound energy. *Extracapsular* surgery involves removing the lens nucleus as a single piece through a larger incision. Extracapsular surgery is usually reserved today for advanced cataracts that cannot be safely phacoemulsified.

Postoperative care typically requires several weeks of topical antibiotic and anti-inflammatory drops. Several weeks after surgery the patient is fit with new glasses. Most surgeons feel it is safest to wait several weeks between eyes when bilateral cataract surgery is to be performed.

Complications of surgery include *corneal decompensation, glaucoma, retinal swelling, retinal* *detachment, infection and bleeding*.

*Congenital cataract*

As with senile cataract, treatment for congenital cataract is surgical. If the cataract is felt to be visually significant removal at the earliest safe time is indicated. Delay in removal of a visually significant congenital cataract will result in *amblyopia* or “lazy eye”.


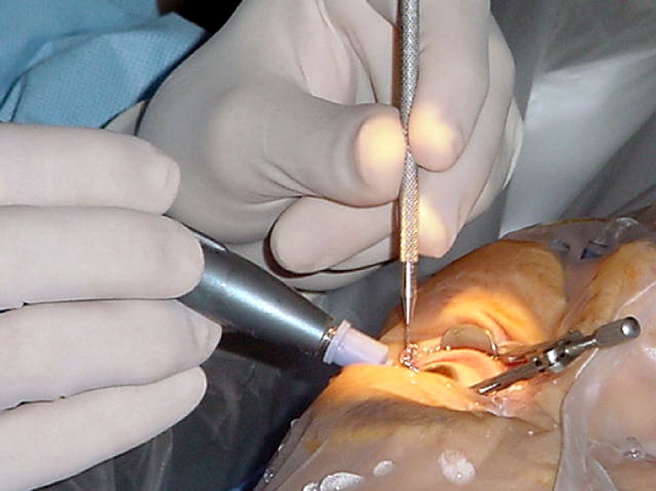


Image by National Eye Institute, retrieved from: <https://commons.wikimedia.org/wiki/File:Cataract_surgery.jpg> on 4/17/2020. Creative Commons License associated: Public Domain -Mark 1.0

**Handout 2: Glaucoma - the Essentials**

**Definitions/Introduction**

*Glaucoma* - a group of diseases which result in characteristic optic nerve damage and visual field loss. It is often but not always associated with elevated intraocular pressure.

*Intraocular Pressure* (IOP) - The eye is a closed space. *Aqueous humor*, produced by the *ciliary body*, flows through the pupil into the anterior chamber and leaves the eye through the *trabecular meshwork* and *Schlemm’s canal*. The pressure inside the eye is a result of the balance between inflow and outflow. Normal intraocular pressure ranges from 6mm Hg to 21mm Hg with an average of approximately 14mm Hg. Intraocular pressure varies diurnally and from day to day.

*Ocular Hypertension* - high intraocular pressure (>21 mmHg)

*Tonometry* - a method to measure pressure. The most common technique uses a Goldmann applanator attached to slitlamp. Other techniques include pneumo-tonometry (“puff of air” test) and a variety of handheld devices including the Tono-Pen and the older, now rarely used Schiotz tonometer.

**Primary Open-Angle Glaucoma** (POAG) - *Prevalence* - approximately one percent of all Americans. Second leading cause of blindness in US. Leading cause of blindness in African Americans.

*Risk factors* - family history, African American heritage, diabetes, age over 45. *Symptoms* - Usually asymptomatic until late in disease

*Signs* - elevated intraocular pressure, normal appearing anterior chamber angle,

**Most common types of glaucoma**

Primary Open-Angle Glaucoma

Normal Tension Glaucoma

Pigmentary Glaucoma

Pseudo-exfoliation syndrome Glaucoma

Acute Angle-Closure Glaucoma Trauma-Related Glaucoma

Uveitis-Related Glaucoma

Congenital Glaucoma

Neovascular Glaucoma

characteristic optic nerve damage (cupping)

and characteristic visual field defects.

*Etiology/Pathophysiology* - the cause of the high intraocular pressure is unknown. Visual loss is due to damage to retinal nerve fibers which make up the optic nerve. The exact mechanism by which high intraocular pressure damages optic nerve fibers is unknown.

*Treatment*- treatment in all forms of glaucoma is directed toward lowering intraocular pressure to arrest further damage to the optic nerve. In POAG initial therapy consists of eye drops which act to decrease aqueous secretion, increase trabecular meshwork outflow or increase alternative outflow paths for aqueous humor.

When topical therapy fails the eye may be treated with *selective laser trabeculoplasty* (SLT). In SLT, the trabecular meshwork is treated with laser and aqueous outflow is increased. The mechanism for improved outflow is not well understood.

Patients who fail topical therapy may also undergo *trabeculectomy* in which a fistula is created between the anterior chamber and the subconjunctival space allowing aqueous humor to bypass the trabecular meshwork on its way out of the eye.

**Normal Tension Glaucoma** (NTG)

*Prevalence* - unknown

*Symptoms* - identical to POAG

*Signs* - identical to POAG except for lack of elevated IOP

*Etiology/Pathophysiology* - unknown but theories abound --

Diurnal fluctuation in IOP

Artifactual low pressures due to thin corneas

True higher susceptibility to optic nerve damage from “normal” IOP

**Pigmentary Glaucoma**

*Risk factors* - typically develops in 20's and 30's. Men more than women. More often in near-sighted patients.

*Symptoms* - usually same as POAG. Occasionally pt may notice blurred vision with exercise. *Signs* - optic nerve and visual field changes identical to POAG, elevated intraocular pressure, iris transillumination defects, heavily pigmented trabecular meshwork *Etiology/Pathophysiology* - rubbing of pigmented layer of iris against lens causes shedding of pigment which may clog the trabecular meshwork

*Treatment* - same as POAG. SLT appears to work better for these patients. Miotic therapy may also be appropriate.

**Pseudo-exfoliation Syndrome Glaucoma**

*Risk factors* - over age 50, European or Russian descent.

*Symptoms* - identical to POAG but often unilateral

*Signs* - optic nerve and visual field changes identical to POAG, elevated intraocular pressure, “dandruff like” material deposited on lens iris and trabecular meshwork *Etiology/Pathophysiology* - clogging of trabecular meshwork with the pseudoexfoliation material. The origin of the material is unknown.

*Treatment* - same as POAG. As in pigmentary glaucoma, SLT appears to work better for these patients.

**Angle Closure Glaucoma**

*Prevalence* - less common that POAG, affects approximately half a million people in US *Risk Factors* - hyperopia, Asian descent, age, family history, acute attacks may be precipitated by anything causing prolonged dilation i.e prolonged time in dark, drugs with anticholinergic effects, dilation for eye exam, emotional stress

*Symptoms* - asymptomatic between acute attacks, during acute attacks patients experience intense pain, blurry vision and perhaps halos around lights

*Signs*

Between attacks - normal or elevated intraocular pressure (depending on chronicity), narrow anterior chamber angles, typical optic nerve damage and visual field defects

During acute attack - very high intraocular pressure, cloudy swollen cornea, conjunctival injection, intraocular inflammation, closed anterior chamber angles *Etiology/Pathophysiology* - as mentioned above the trabecular meshwork is located in the angle where the cornea and iris meet. In most people the angle of approach is approximately 45 degrees. If this angle is decreased, the peripheral iris may block access to the trabecular meshwork. This is more likely to happen when the eye is dilated and the iris is crowded peripherally. Age may play a part as the lens becomes thicker with age and may push the iris forward.

*Treatment* - a peripheral iridectomy (PI), a hole in the peripheral iris, usually created with a laser, is the definitive treatment and reestablishes flow from the posterior to anterior chamber. Patients with narrow (occludable) angles should have PI’s placed prophylactically to prevent acute narrow angle glaucoma attacks. Patients with prolonged angle closure may form adhesions from the iris to the cornea permanently occluding the trabecular meshwork and causing a particularly intractable form of glaucoma.

**Trauma-Related Glaucoma**

**Uveitis-Related Glaucoma**

**Congenital Glaucoma**

**Neovascular Glaucoma**

Please read your text for descriptions of these types of glaucoma.

**Visual field testing and glaucoma**

Early optic nerve damage results in characteristic patterns of peripheral vision loss. This visual field loss is usually too subtle to be noticed by the patient or to be picked up by “finger counting” visual field testing. Visual field testing is typically done using an automated *perimeter*. The patient sits in front of a dome. Lights of varying intensities appear around the dome. The patient is asked to press a button each time they detect a light. The perimeter keeps track of the patient’s

threshold at each point in the visual field and presents it graphically for review and interpretation.


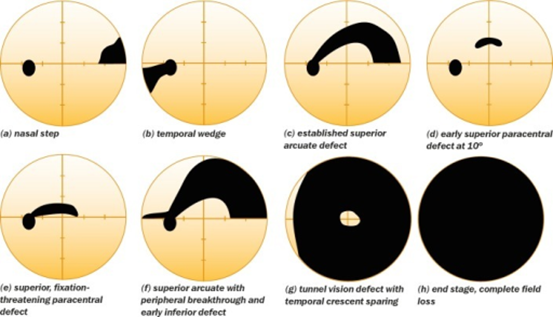


Glaucomatous visual field deficits

Image by Broadway DC retrieved from: <https://commons.wikimedia.org/wiki/File:Glaucomatous_field_defects.png>

on 4/19/2020. Creative Commons License associated: CC BY 2.0

**Optic Nerve**

**Examination of the optic nerve** Examination of the optic nerve is the single most sensitive and specific way to detect glaucoma. Furthermore, it is currently the


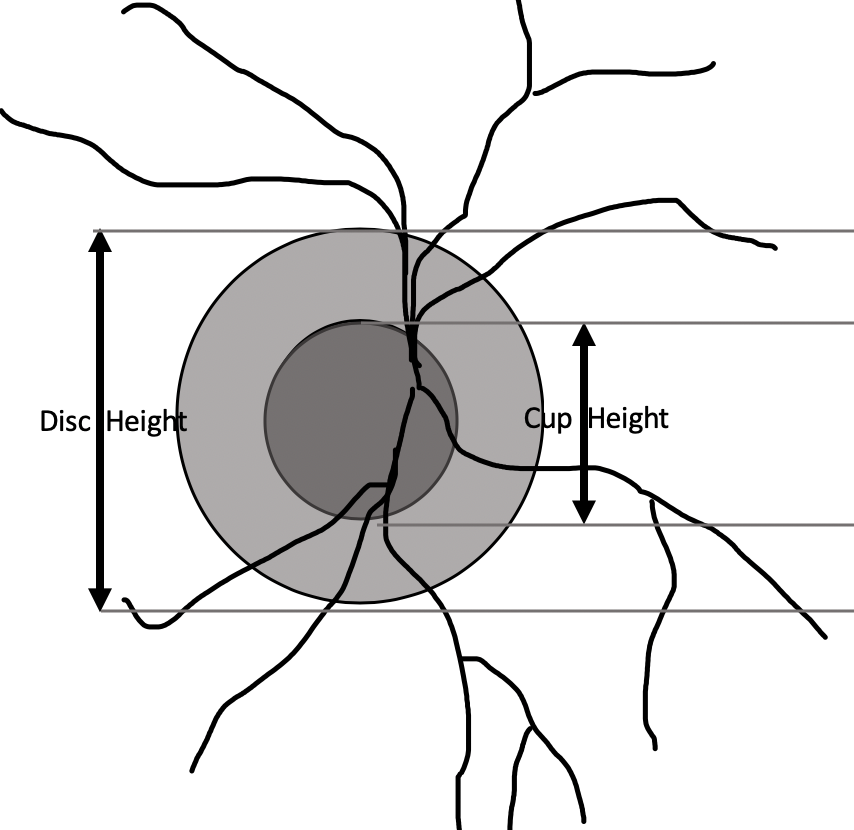


only practical way to screen for glaucoma

in a primary care setting.

Patients with cup to disc ratios of 0.4 or more and patients with cup to disc asymmetry of 0.2 or more require further

Figure Author Owned

evaluation. Hemorrhage on the optic nerve may also be a sign of glaucoma. More subtle signs of a glaucomatous nerve include notching and pallor.

**Screening**

|  | **No risk factors** | **Risk factors present** |
| --- | --- | --- |
| **Under age 45** | Every 4 years | Every 2 years |
| **45 and older** | Every 2 years | Every year |

The Glaucoma Foundation recommends this schedule to determine how often a patient should have an ophthalmologic exam to check for glaucoma.

Risk factors include: Family history, African American heritage, myopia, diabetes, hypertension, long term steroid use, previous eye injury.

**Glaucoma Medications**

| **Medication class** | **Examples** | **Mechanism of action** | **Selected side effects** |
| --- | --- | --- | --- |
| β blockers | timolol, levobunolol, cateolol, metipranolol, betaxolol | decrease aqueous formation | asthma exacerbation, CHF exacerbation, bradycardia, heart block, fatigue, impotence, alopecia, lipid profile changes |
| a-adrenergic agents | brimonidine, apraclonidine | decrease aqueous formation | headache, poor dark adaptation, retinal detachment |
| carbonic anhydrase inhibitors | dorzolamide, brinzolamide | decrease aqueous formation | high topical allergy rate, drowsiness |
| prostaglandins | latanoprost, travoprost, bimatoprost | increase non-trabecular outflow | renal stones |
| miotics | pilocarpine | increase trabecular outflow | red eye, turns hazel eyes brown, potentiates uveitis |

**Handout 3: Macular Degeneration - the Essentials**

**Definitions**


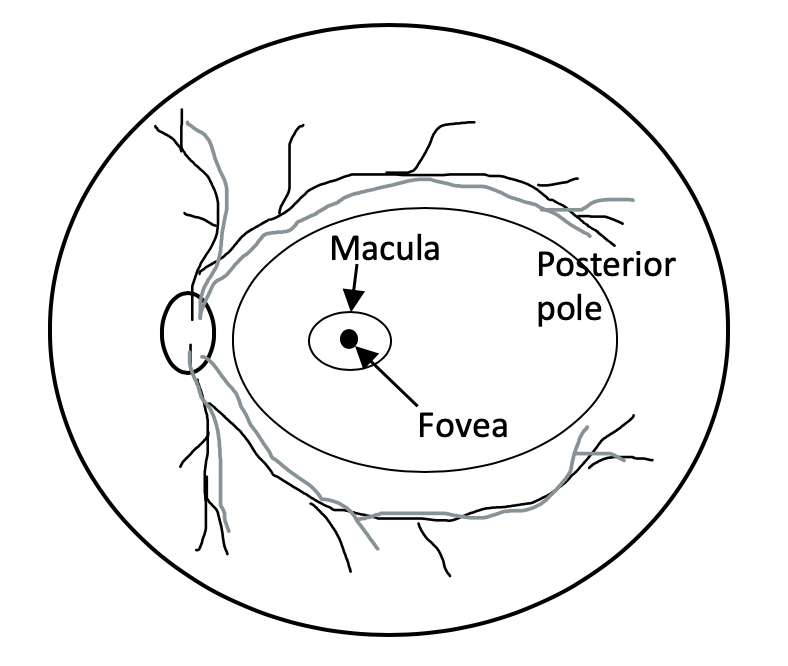


*Macula*: Area of the retina about 3 mm temporal to the optic nerve head. It is responsible for central vision, fine spatial discrimination and color vision.

*Fovea*: Central depression within macula containing only cones responsible for finest resolution sight.

*Age-related Macular Degeneration:* loss of central vision due to age related changes in the macula.

**Epidemiology**

Leading cause of legal blindness in patients over

Figure Author Owned

60. Affects 28% of people between ages 75 and

Smoking has been shown to be positively correlated with ARMD.


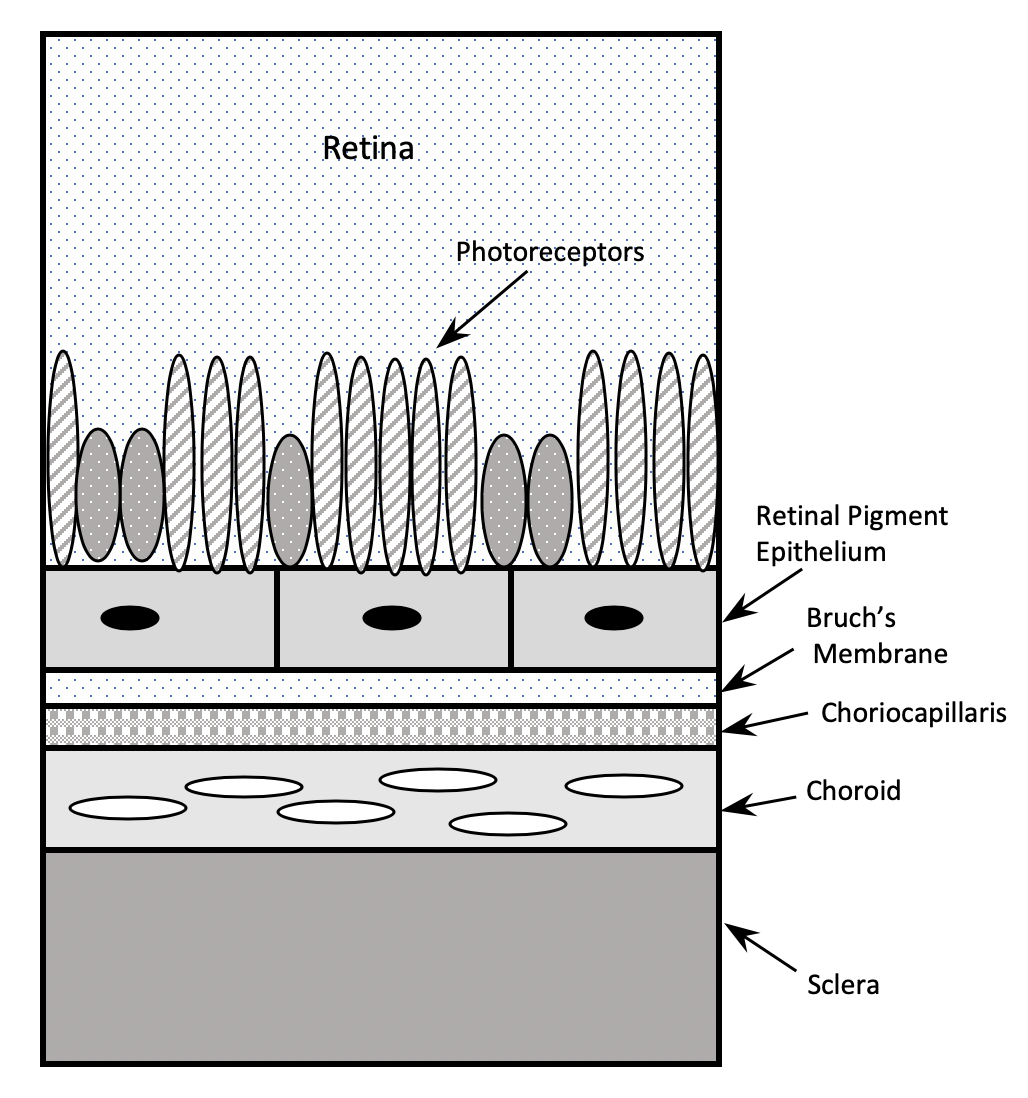


**Etiology/Pathophysiology**: Aging results in a number of changes in the retina.

1) *Drusen* - localized deposits in *Bruch’s* membrane - the layer between the *retinal* *pigment epithelium* (RPE) and *choroid*. They are classified by appearance into *hard* (small and discrete) and *soft* (irregular with indistinct edges) types.

2) Retinal Pigment Epithelium Changes - hyper and hypo pigmentation due to atrophy. The RPE serves a number of metabolic functions for the photoreceptors. When the RPE atrophies, photoreceptors lose function.

3) Weakening of Bruch’s membrane. The *dry* or *atrophic* form of macular degeneration is characterized by drusen and RPE changes. Visual acuity is variably reduced depending on the location and extent of the changes. Less commonly, a severe form of dry

Figure Author Owned

macular degeneration called geographic atrophy can result in total loss of central vision.

The *wet* or *neovascular* form of macular degeneration is characterized by extension of vessels from the choroidal circulation through the weakened Bruch’s membrane forming a *choroidal* *neovascular membrane (CNVM)* or *net*. The new blood vessels leak fluid, lipid and blood under

the retina. This is turn leads to photoreceptor death, subretinal fibrosis and scarring and


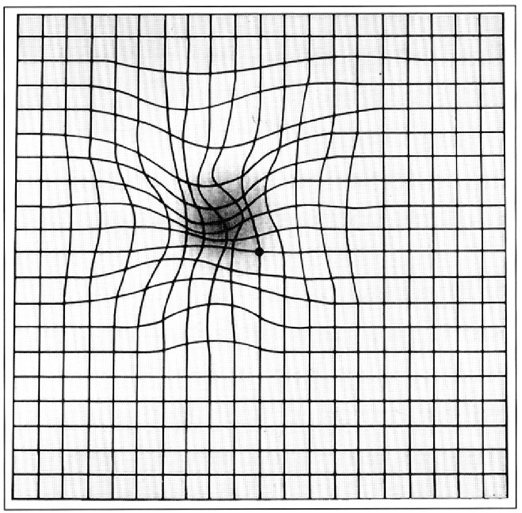
detachment of the retinal pigment epithelium. Visual loss in the wet form of macular degeneration is dependent on the location of the leaking vessels but is generally more sudden and more severe than the dry form.

Distorted Amsler Grid

**Symptoms**: Patients with the dry form of macular degeneration may note gradual mild decrease in central vision and will occasionally notice distortion causing waviness of straight lines (*metamorphopsia*). Patients with the wet form of macular degeneration may notice sudden loss of central vision with a central blind spot (*scotoma*) and severe metamorphopsia.

Image by National Eye Institute, retrieved from: <https://search.creativecommons.org/photos/c78eb00d-d7e4-430c-ab36-44eab55f32ce> on 4/16/2020. Creative Commons License associated: CC BY 2.0

**Treatment**: There is currently no treatment for the dry form of macular degeneration.

Until recently, wet ARMD was treated primarily with laser treatment aimed at sealing off and causing regression of the CNVM. The benefit from laser was small and patients often had permanent blind spots as a result of the treatment. This treatment is used much less often in current practice.

A newer treatment called photodynamic therapy (PDT) involves intravenous injection of a photoactivatable dye, verteporphorin, with an affinity for new blood vessels. Subsequent exposure of the CNVM to the correct frequency of light causes activation of the dye molecules and closure of the CNVM. When it works this treatment results in stabilization but rarely improvement in vision.

Currently, most patients with ARMD are treated with antibodies to vascular endothelial growth factor (VEGF). The anti- VEGF antibodies are injected into the vitreous cavity and cause regression of the CNVM with much less scarring than the older laser treatment. This newer treatment actually results in an improvement in vision rather than the slowing of visual loss or stabilization characteristic of older treatments.

Based on current data, patients with ARMD at any stage should be encouraged to stop smoking. A specific combination of vitamins A, E, C, Zinc and Copper has been shown to have some prophylactic value in patients with high-risk characteristics. The downside of this vitamin is minimal (except for patients who smoke for whom the risk of lung CA is increased) . The use of sunglasses in bright sun conditions has not been proved to be of clear prophylactic value but some ophthalmologists will recommend these to decrease chronic photic insult to the retina.

There are many claims that other vitamins and supplements are useful but none have yet been shown to be beneficial in well performed studies. As is often the case with chronic incurable diseases there will always be some practitioners who are willing and able to sell expensive treatments of no proven value.

**Handout 4: Amblyopia & Strabismus - the Essentials**

**Amblyopia**

*Definition* **-** loss of visual acuity not correctable by glasses in an otherwise healthy eye. *Epidemiology* - affects approximately 2% of US population. Develops during infancy and childhood

*Etiology* - atrophy of visual pathways from one or both eyes due to disuse during the critical period between birth and ten years old. The three major causes include

1. Strabismus or misalignment of the eyes - faced with double vision from misalignment of the eyes a child’s brain will suppress the image from one eye to maintain single vision. Over time, visual pathways from the suppressed eye atrophy.
2. High or asymmetric refractive error - if one eye is very farsighted or nearsighted compared to the other the brain will suppress the image from the blurry eye. If both eyes are very highly farsighted or (less commonly) nearsighted then visual pathways may not develop well in either eye.
3. Occlusion - if, during the critical period, vision is occluded or blurred by a congenital cataract, corneal scar or ptosis (droopy eyelid) visual pathways from the occluded eye do not develop correctly.

In all forms of amblyopia, the visual loss may persist even if the cause is fixed. Visual loss not corrected by age 10 is generally not recoverable.

*Symptoms* - children will not complain of poor vision from amblyopia and unless it is accompanied by obvious signs such as strabismus, an obvious cataract or ptosis parents may not aware of their child’s poor vision.


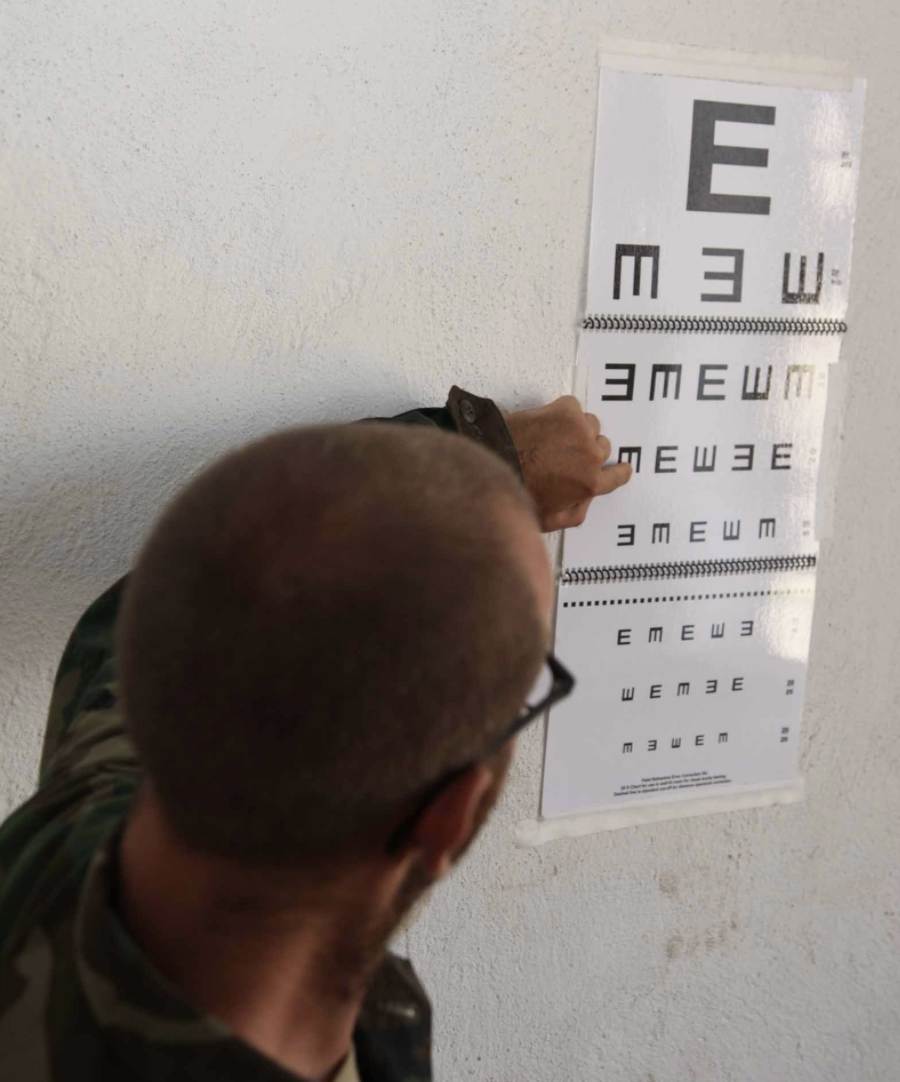


*Signs*

Visual acuity - visual acuity cannot be assessed in newborns. By the age of several months old most infants develop visual interest. From this age to about two years old, infants visual acuity can be assessed by a the child’s ability to maintain fixation on and follow a toy. The ability to fix and follow should appear equal between the eyes. A child with poor acuity in one eye may object or interfere when the good eye is covered. Acuity cards with pictures or the tumbling E can be used with children who are verbal but don’t know the letters of the alphabet. At 4 or 5 years old, many children can be tested using the standard acuity chart.

Image by Benjamin Tuck retrieved from: <https://www.dvidshub.net/image/464337/village-medical-outreach-herat-province> on 4/19/2020. Creative Commons License associated: CC0 1.0

Other signs - the physician in the primary care setting may note other signs related to the cause of the acuity loss and an organic cause for the decreased acuity should be sought by checking the pupillary reflexes, directly examining the eye with a penlight and checking the red reflex for leucocoria. Obvious strabismus may be seen without special testing. Smaller amounts of strabismus may be detected using the cover test (see below).

Examination in the ophthalmologist’s office includes all of this as well as determining the refractive state of the eye using retinoscopy, a slit-lamp exam and a dilated eye exam.


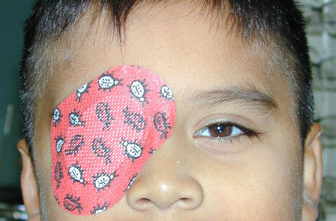
*Treatment* - successful treatment of amblyopia requires treatment of the underlying cause (correction of refractive error, cataract removal, etc) when possible and occlusion of the good eye with a patch to force the brain to use the amblyopic eye. Recent studies have suggested that blurring the good eye with atropine drops may be as effective as patching for some children. Prognosis is better for children with better starting vision and for children treated as earlier during the critical period. Amblyopia untreated by age 7 may be uncorrectable but success has been documented in children up to age 10.

Image by University of Michigan Kellog Eye Center, retrieved from: <http://kellogg.umich.edu/theeyeshaveit/tehi_images/amblyopia.jpg> on 4/17/2020. Creative Commons License associated: CC BY 3.0


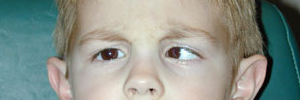


**Strabismus**

*Definitions*

Strabismus - misalignment of the eyes Esotropia - inward deviation; Exotropia - outward deviation; Hypertropia - vertical deviation

Image by University of Michigan Kellog Eye Center, retrieved from: <http://kellogg.umich.edu/theeyeshaveit/tehi_images/esotropia-uncorrected.jpg> on 4/17/2020. Creative Commons License associated: CC BY 3.0

*Etiology* - numerous including:

1. Sensory - poor vision in one or both eyes decreases the stimulus for the eyes to track together. Children with poor vision in one eye are often esotropic. Adults who lose vision in one eye are more likely to become exotropic.
2. Accommodative - accommodation (focusing on a near object) and convergence (aligning the eyes together towards a near object) are closely tied together in the brain. Esotropia can result if a child is very highly farsighted and must exert accommodative power to focus or if the gain for this reflex is mis-set in the brain
3. Restrictive - an extraocular muscle is trapped (trauma) or fibrotic (congenital or thyroid)
4. Paretic - one or more extraocular muscles are partially or fully denervated (MS, myasthenia, diabetes, CNS tumors, stroke)

Of these sensory deviations and accommodative deviations are most common in children.

*Symptoms* - parents may notice that a child’s eyes are turned in or turned out. The deviation may

be constant or intermittent and manifest only when the child is tired.

Signs - the physician in the primary care setting can check for eye misalignment using a variety of techniques

1. Inspection - an large angle constant deviation will be obvious on inspection


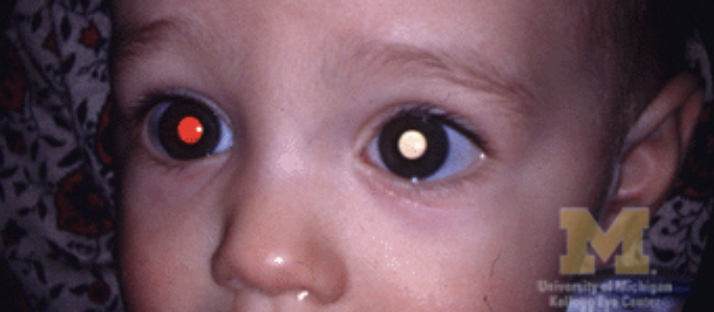


1. Corneal light reflex - a penlight is directed toward the patient. The reflected light on the cornea should appear at the same place in the same position relative to the pupil. If an eye is deviated the reflection will be in a different position on each eye.
2. Cover test - with the child fixating on an object such as a hand held toy, cover one of the child’s eyes. If the other eye

Image by University of Michigan Kellog Eye Center, retrieved from: <http://kellogg.umich.edu/theeyeshaveit/otherc/retinoblastoma.html> on 5/29/2020. Creative Commons License associated: CC BY 3.0

moves to fixate the object there is strabismus. If the eye moves

inward the deviation is an exotropia, if the eye moves outward

the deviation is an esotropia.


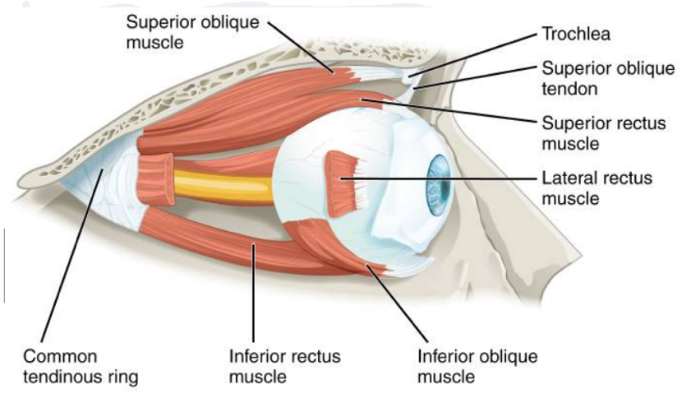
Other signs - the physician in the primary care setting may note other signs related to the cause of the strabismus. Visual acuity must be checked to

rule out a sensory deviation. Pupillary reflexes, directly examining the eye with a penlight and checking the red reflex for leukocoria are again important. Strabismus is a common presentation for many causes of poor vision in infancy and childhood including amblyopia, congenital cataract and retinoblastoma.

As above, examination in the ophthalmologist’s office includes a full eye exam with retinoscopy, slit-lamp exam and a dilated eye exam. In certain circumstances, an exam under anesthesia may be needed to fully examine the eye.

Image by Open Stax, retrieved from: <https://www.ncbi.nlm.nih.gov/books/NBK470534/figure/article-21502.image.f1/>on 4/17/2020. Creative Common License associated: CC BY 3.0


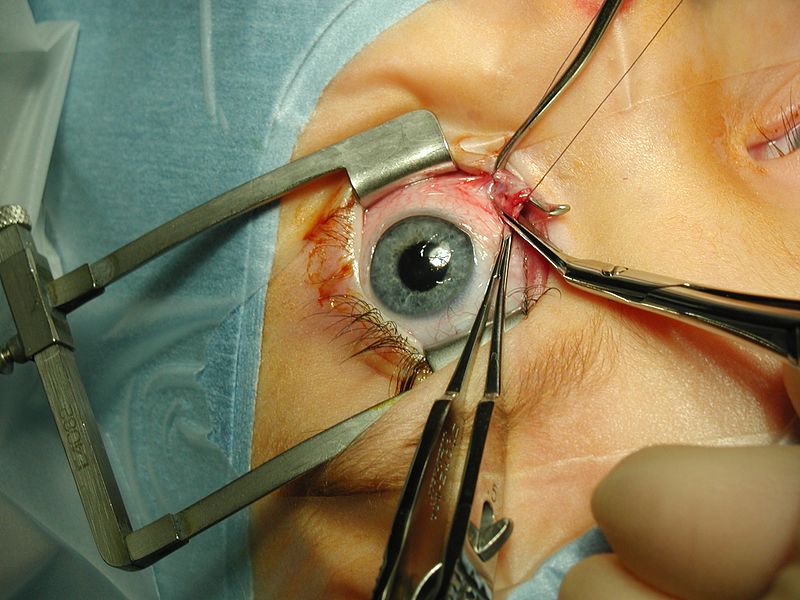


*Treatment* - treatment of sensory strabismus is directed toward improving the vision in the affected eye. Eyeglass correction of refractive errors, patching for amblyopia, or cataract removal. Accommodative strabismus is often correctable with eyeglass correction alone. Once equal vision is obtained in the two eyes, if a deviation persists the eyes can be realigned surgically by effectively weakening (recessing) or strengthening (resecting) the extraocular muscles. “Vision training” or eye exercises prescribed by some practitioners is generally considered worthless by the ophthalmic community.

Image by Bticho retrieved from: <https://commons.wikimedia.org/wiki/File:Desinsertion_du_muscle_CO.jpg>

on 4/17/2020. Creative Commons License associated: CC0 1.0


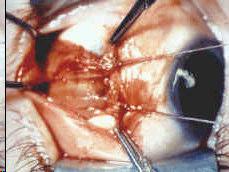


**Handout 5: Acute Visual Loss - the Essentials**

Please use the 9 photos in the Acute Vision Loss Case Conference to fill out the chart below. Include the diagnosis, presenting history and common patient demographic associated with this condition, the timing of onset of the condition (minutes to hours, hours to days), if you would expect an APD, and the management and prognosis.

| **Photo** | **Diagnosis** | **History/Demographic** | **Timing** | **APD?** | **Red Reflex?** | **Management/Prognosis** |
| --- | --- | --- | --- | --- | --- | --- |
| **A** |  |  |  |  |  |  |
| **B** |  |  |  |  |  |  |
| **C** |  |  |  |  |  |  |
| **D** |  |  |  |  |  |  |
| **E** |  |  |  |  |  |  |
| **F** |  |  |  |  |  |  |
| **G** |  |  |  |  |  |  |
| **H** |  |  |  |  |  |  |
| **I** |  |  |  |  |  |  |

**Handout 6: Diabetes and the Eye - the Essentials**

Diabetic retinopathy is the leading cause of blindness in working age Americans. Appropriate referral and treatment reduce visual loss by 50%.

Incidence of retinopathy is 23% in type I diabetics after 5 years and 80% after 15 years.

Etiology/Pathophysiology - Hyperglycemia leads to vascular endothelial dysfunction. Endothelial dysfunction leads to 1) leakage of blood or lipid into retina 2) capillary occlusion and ischemia of retina.

**Stages:**

1) No retinopathy – retina appears normal, endothelial dysfunction can be detected using special techniques

2) Background retinopathy


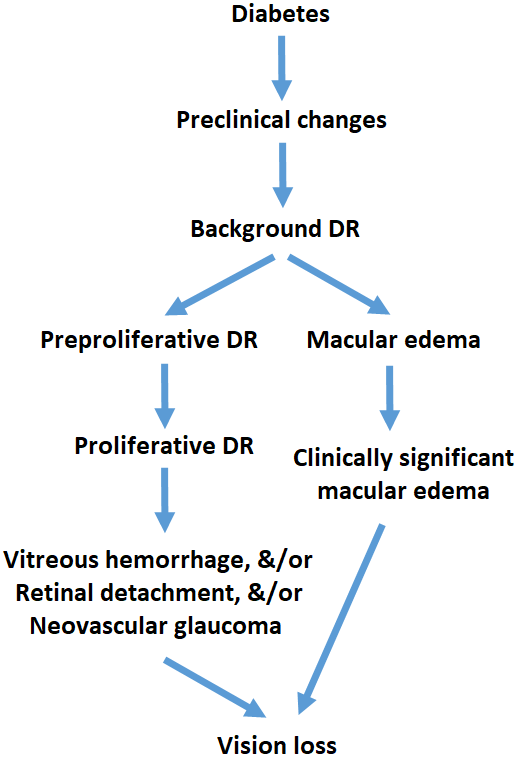


**• microaneurysm (MA)** – tiny red dots, saccular outpouching of capillaries, these leak

**• hemorrhages** – evidence of leakage

**- dot** – tiny red dot difficult to tell from MA in inner retinal layers

**- blot** – larger red splotch in inner retina

**- flame** – elongated hemorrhage in nerve fiber layer

3) Exudative retinopathy – leakage of plasma and lipid from MA

**• hard exudates** – bright yellow dots with distinct borders, lipid deposits

**• retinal thickening** – accumulation of fluid in retinal layers

When these occur in the central retinal area or macula this is known as **macular edema**. When exudate or thickening occur close to the fovea the chance of visual loss is high and is called **clinically significant macular edema (CSDME)**

Figure Author Owned

1. **Preproliferative retinopathy (PPDR)**

•cotton wool spots - retinal nerve fiber layer infarcts •venous dilation and tortuosity

High risk for progression to proliferative retinopathy

5) **Proliferative retinopathy (PDR)** - as a response to ischemia, new blood vessels grow.

These blood vessels are fragile and grow into vitreous. Vitreous traction on these vessels can result in bleeding (vitreous hemorrhage) or detachment of the retina. Approximately 50% of patients with PDR progress to blindness in five years

Table adapted from the American Academy of Ophthalmology 2019 Diabetic Retinopathy Preferred Practice Pattern.

Flaxel CJ, Adelman RA, Bailey ST, et al. Diabetic Retinopathy Preferred Practice Pattern® *Ophthalmology*. 2020;127(1):P66-P145. doi:10.1016/j.ophtha.2019.09.025

| **Recommended Screening and Treatment for Patients with Diabetes** | | | |
| --- | --- | --- | --- |
| **Severity** | **Follow up (months)** | **Laser** | **Anti-VEGF Therapy** |
| No Diabetic Retinopathy (DR) | 12 | No | No |
| Mild Nonproliferative DR | 12 | No | No |
| Moderate Nonproliferative DR | 6 - 12 | No | No |
| Severe Nonproliferative DR | 3 - 4 | Consider PRP | Sometimes |
| Proliferative DR | 2 - 4 | PRP | Sometimes |
| Macular Edema (non-center involved) | 3 - 6 | Consider Focal | Sometimes |
| Macular Edema (center involved) | 1 | Focal | Yes |

*All diabetic patients > 30 years old or < 30 years old with diabetes for 5 years or more require screening.

*Diabetic women should be screened prior to or during the first trimester of pregnancy. Screening not arequired for gestational diabetics.

*follow-ups are dilated retinal exams with ophthalmologist *focal - focal laser in macular area to seal microaneurysms

*PRP - panretinal photocoagulation to decrease angiogenic drive and cause regression of aneovascularization

**Other manifestations of DM in the eye**

1. Rapid changes in eyeglass prescription with blood sugar fluctuation causing acute blurry vision
2. Cornea - dry eye, poor wound healing, contact lens intolerance
3. Iris - poorly reactive pupils, light-near dissociation
4. Lens - diabetics get cataracts earlier
5. Glaucoma - slightly more likely to get open-angle glaucoma, in severe DM neovascularization of the iris and angle can occur causing neovascular glaucoma
6. EOM deficits - can have spontaneous paresis of CN III, IV or VI due to microangiopathy
