# Supplementary material for: A Required Ophthalmology Rotation: Providing Medical Students with a Foundation in Eye-Related Diagnoses and Management
Source: MedEdPORTAL. 2021 Feb 12;17:11100. doi: 10.15766/mep_2374-8265.11100 (PMC7880261; doi:10.15766/mep_2374-8265.11100)
Supplement: Supplementary file 1 — Ophthalmology Slides Instructors Guide.docxOphthalmology Handout.docxOphthalmology Slides.pptxOphthalmology Sessions.docxOphthalmology Sessions Answer Key.docxOphthalmology Sessions Student Handouts.docxOphthalmology Final Examination.docxStudent Postrotation Feedback Form.docx [file mep_2374-8265.11100-s001.zip › G. Ophthalmology Final Examination.docx]

**NAME ______________________________________ DATE _______________**

Specialty Care Clerkship Final Examination – Ophthalmology Portion

**Please answer questions 1-9 with short answers -- a few words or a sentence. Long explanations are not required.**

**1)** An 18-year-old woman presents to the emergency room with a red, uncomfortable right eye of one day’s duration. She notes mildly decreased vision in this eye that clears briefly with blinking. On exam her vision is 20/25 at near OD and 20/20 OS. The right eye is shown in the figure.


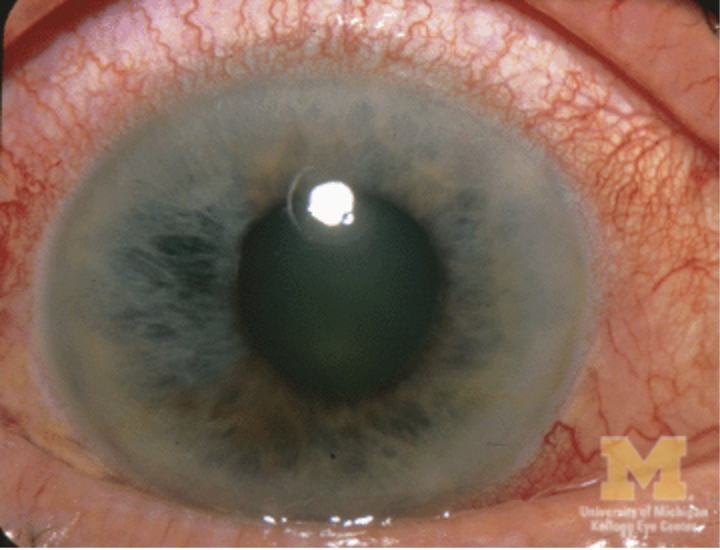


**a)** Name the single most likely diagnosis.

Viral conjunctivitis

**b)** What additional historical information could you obtain to

support this diagnosis?

Timing, upper respiratory symptoms, sick contacts,

Image by University of Michigan Kellogg Eye Center, retrieved from: <http://kellogg.umich.edu/theeyeshaveit/redeye/angleclosure_glaucoma.html> on 5/29/2020. Creative Commons License Associated: CC BY 3.0

changes and nature of changes in vision, contact lens use,

previous eye history

**c)** Briefly, outline initial management for this condition.

Cold artificial tears, discussion of red flag symptoms

**2)** List three ocular signs that distinguish benign causes of red eye from more serious causes.

a. Decreased visual acuity

b. Real pain

c. Corneal cloudiness

*Points given for other reasonable answers as well, although these are the three stressed in lectures


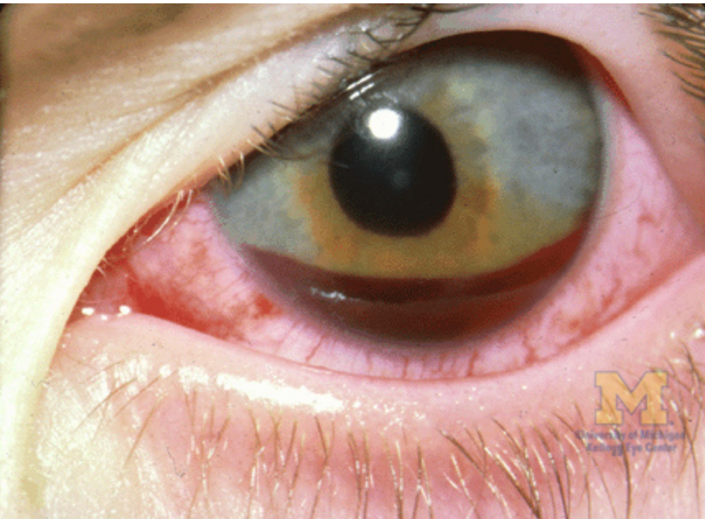


**3)** A 28-year-old carpenter complains of a painful, red left eye with decreased vision since she felt something fly into it earlier today. She was hammering nails at the time of the accident. On exam the vision is 20/400 in the left eye and 20/20 in the right. Topical anesthetic decreases but not eliminate the discomfort. The affected eye is shown in the figure.

A

**a)** Name the finding demonstrated by *arrow A*.

Image by University of Michigan Kellogg Eye Center, retrieved from: <http://kellogg.umich.edu/theeyeshaveit/trauma/hyphema.html> on 4/25/2020. Creative Commons License associated: CC BY 3.0

B

Subconjunctival hemorrhage

**b)** Name the finding demonstrated by *arrow B*.

Hyphema

**c)** The finding demonstrated by *arrow B* suggests the need for a careful eye exam to rule out what condition?

Ruptured globe. Some points for sickle cell trait/disease

**4)** Name the most important measure of the health of the visual system and the first thing that should be tested during an eye examination.

Visual acuity

**5)** A 65-year-old woman notes sudden painless loss of vision in her left eye earlier this afternoon. The right eye is unaffected. Her past ocular history is significant for bilateral cataract surgery. She has hypertension and high cholesterol. On review she complains of recent tiredness and headache. On exam her vision is 20/30 OD and count fingers only at 3 feet OS. Fundoscopic exam is shown in the figure.


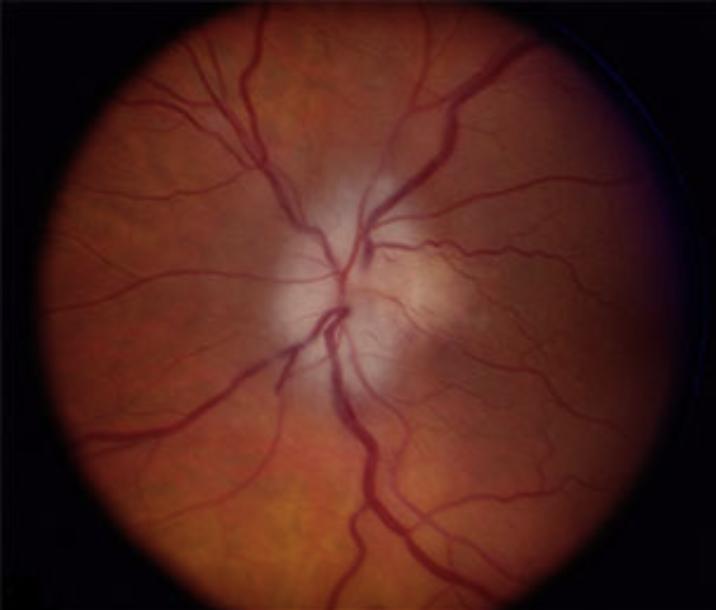


**a)** Name the most likely diagnosis.

Ischemic optic neuropathy

Image by University of Michigan Kellogg Eye Center, retrieved from: <http://kellogg.umich.edu/theeyeshaveit/system/giant_cell_arteritis.html> on 4/25/2020. Creative Commons License associated: CC BY 3.0

**b)** What systemic disorder should be considered?

Temporal arteritis

**c)** Circle the pupil exam that is most consistent with this patient’s history and findings.

Images Author Owned

Right image

**6)** A 72-year-old man is seen for an annual eye examination. He has no complaints related to his vision. His best corrected vision after refraction is 20/50 OD and 20/40 OS. The slit-lamp exam is shown.


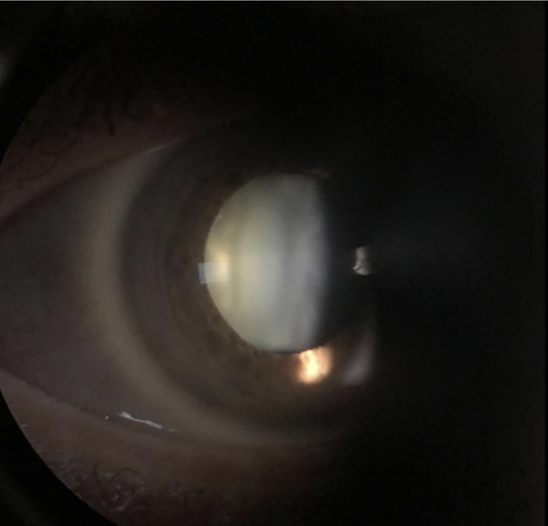


**a)** Name the most likely diagnosis.

Cataract

**b)** What is the appropriate recommendation regarding a surgical

intervention at this time?

Image Author Owned

Something along the lines of “Cataract surgery should be offered when the patient experiences functional limitations due to vision”

**7)** Name at least one optic nerve finding commonly associated with glaucoma.

Cupping, asymmetry of cupping

**8)** Name the leading cause of blindness in African Americans.

Primary open angle glaucoma

**9)** A 48-year-old man with a 10-year history of diabetes is seen for an eye exam. He notes that his vision decreased suddenly two days ago in the left eye. Vision in the right eye has decreased slowly over the past year. There is no red reflex and no view to the fundus of the left eye. Photos of the retina and optic nerve of the right eye are shown.

B


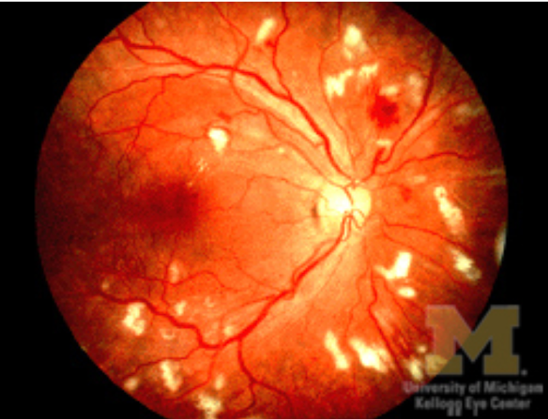


**a)** What is the most likely complication of diabetes in the left eye?

A

Vitreous hemorrhage

Image by University of Michigan Kellogg Eye Center, retrieved from: <http://kellogg.umich.edu/theeyeshaveit/opticfundus/cotton_wool.html> on 5/30/2020. Creative Commons License associated: CC BY 3.0

**b)** Name the finding demonstrated in the right eye by *arrow A.*

Exudate, full points for cotton wool spot

**c)** Name the finding demonstrated in the right


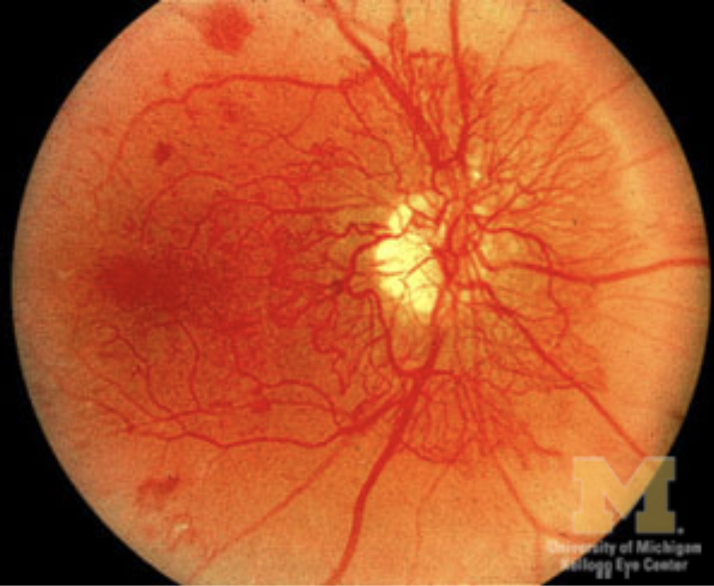


eye by *arrow B.*

Intraretinal hemorrhage

**d)** Name the vascular finding demonstrated

on and around the optic nerve in the photo directly

to the right.

Image by University of Michigan Kellogg Eye Center, retrieved from: <http://kellogg.umich.edu/theeyeshaveit/opticfundus/retinal_neovascularization.html> on 5/30/2020. Creative Commons License associated: CC BY 3.0

Neovascularization

**e)** Which of the three findings above (b-d) is responsible for the decreased vision and loss of red reflex in the right eye?

Neovascularization

**10)** Which of the following patients is at highest risk for primary open-angle glaucoma?

A. Positive Family Hx; African‑American; Non‑diabetic; hyperopia (far‑sighted)

B. Positive Family Hx; Caucasian; Diabetic; 20/20 vision without glasses;

C. No family Hx; African‑American; Diabetic; myopia (near‑sighted)

D. No family Hx; Caucasian; Non‑diabetic; 20/20 vision without glasses
