# Supplementary material for: A Required Ophthalmology Rotation: Providing Medical Students with a Foundation in Eye-Related Diagnoses and Management
Source: MedEdPORTAL. 2021 Feb 12;17:11100. doi: 10.15766/mep_2374-8265.11100 (PMC7880261; doi:10.15766/mep_2374-8265.11100)
Supplement: Supplementary file 1 — Ophthalmology Slides Instructors Guide.docxOphthalmology Handout.docxOphthalmology Slides.pptxOphthalmology Sessions.docxOphthalmology Sessions Answer Key.docxOphthalmology Sessions Student Handouts.docxOphthalmology Final Examination.docxStudent Postrotation Feedback Form.docx [file mep_2374-8265.11100-s001.zip › H. Student Postrotation Feedback Form.docx]

**Ophthalmology Post-Clerkship Evaluation Form**

Please indicate how you agree with the following statement: I believe the overall teaching in the ophthalmology clinical settings was good quality.

5 – Strongly Agree 4 – Agree 3 – Neutral 2 – Disagree 1 – Strongly Disagree

The overall quality of ophthalmology lectures was:

5 - Outstanding 4 – Good 3 – Satisfactory 2 – Fair 1 – Poor

Describe any aspects of this clerkship that had a particularly positive impact on your learning experience:

Describe any aspects of this clerkship that had a particularly negative impact on your learning experience:

Feedback about how this clerkship might be improved:
